# Cohort study investigating the relationship between cholesterol, cardiovascular risk score and the prescribing of statins in UK primary care: study protocol

**DOI:** 10.1136/bmjopen-2016-013120

**Published:** 2016-11-17

**Authors:** Samuel Finnikin, Ronan Ryan, Tom Marshall

**Affiliations:** Institute of Applied Health Research, University of Birmingham, Birmingham, UK

**Keywords:** PREVENTIVE MEDICINE, PRIMARY CARE, THERAPEUTICS

## Abstract

**Introduction:**

Risk scoring is an integral part of the prevention of cardiovascular disease (CVD) and should form the basis for the decision to offer medication to reduce cholesterol (statins). However, there is a suggestion in the literature that many patients are still initiated on statins based on raised cholesterol rather than a raised CVD risk. It is important, therefore, to investigate the role that lipid levels and CVD risks have in the decision to prescribe. This research will establish how cholesterol levels and CVD risk independently influence the prescribing of statins for the primary prevention of CVD in primary care.

**Methods and analysis:**

The Health Improvement Network (THIN) is a database of coded primary care electronic patient records from over 500 UK general practices. From this resource, a historical cohort will be created of patients without a diagnosis of CVD, not currently receiving a prescription for statins and who had a lipid profile measured. A post hoc QRISK2 score will be calculated for these patients and they will be followed up for 60 days to establish whether they were subsequently prescribed a statin. Primary analysis will consist of predictive modelling using multivariate logistic regression with potential predictors including cholesterol level, calculated QRISK2 score, sociodemographic characteristic and comorbidities. Descriptive statistics will be used to identify trends in prescribing and further secondary analysis will explore what other factors may have influenced the prescribing of statins and the degree of interprescriber variability.

**Ethics and dissemination:**

The THIN Data Collection Scheme was approved by the South-East Multicentre Research Ethics Committee in 2003. Individual studies using THIN require Scientific Review Committee approval. The original protocol for this study and a subsequent amendment have been approved (16THIN009A1). The results will be published in a peer review journal and presented at national and international conferences.

**Strengths and limitations of this study**

- The use of a large, validated and generalisable set of general practices gives an unparalleled insight into current real-life medical practice.
- The use of a large cohort will allow the impact of lipid profiles and cardiovascular disease risk estimation to be modelled in an insightful and novel way.
- Insight into the decision to prescribe statins is limited to information that is coded in the electronic patient record and therefore much of the decision-making process will remain unknown.
- Recording of data may be incomplete and therefore some approximations will have to be made.

## Introduction

Predicted risk of cardiovascular disease (CVD) is determined from a combination of age, sex and cardiovascular risk factors.^1^ ^2^ Risk of CVD is the best predictor of the benefits of statins because statins have the same proportional effect on people at low and high risk.^3^ Because of this, assessment of predicted risk of CVD has been a fundamental part of national clinical guidance on CVD prevention in primary care since 1998.^4^ In the UK, an absolute risk of CVD greater than a threshold was, and remains, a criterion for offering statin treatment and, in combination with raised blood pressure, a criterion for antihypertensive treatment.^5–7^ Risk assessment is also embedded in clinical guidelines around the world including New Zealand, Europe and America.^8–10^ A variety of different risk calculators are available and while QRISK2^11^ is the calculator of choice in England and Wales, elsewhere, others such as Framingham,^1^ Score,^12^ ASSIGN^13^ or Procam^14^ are recommended.

Statins are the only class of lipid-lowering medications recommended for use in UK primary care^5^ and are the most widely prescribed medication in England.^15^ A small proportion of patients in England have to pay a prescription charge for their medications but the majority receive their medication free.^16^ Statins are used to lower lipids as part of a CVD primary prevention strategy. As estimated CVD risk is the best predictor of likely benefits of treatment this information is necessary for effective shared decision-making when considering the use of statins.^17^ Evidence suggests that usage of risk scoring improves accuracy of perceived CVD risk and medical prescribing without causing harms.^18–20^

In the context of primary prevention, there is evidence both of undertreatment of high-risk patients (above the treatment threshold) and inappropriate treatment of low-risk patients (below the treatment threshold).^21–24^ Moreover, patients with the highest absolute CVD risk are at greatest risk of being undertreated.^25^ One reason for this is that risk scores may not be consistently used by clinicians to guide decision-making.^26^ Reasons for not using risk score calculators include a belief that risk scoring oversimplifies the decision and may lead to overprescribing^27^ and confusion over how best to use risk score calculators.^28^

Additionally, one fundamental barrier to using risk scores is a focus on individual risk factors over absolute risk score.^22^ ^29^ It may appear logical that decisions about initiating cholesterol-lowering medication should be based on the patient's cholesterol level, and indeed this was the preferred approach in the pre-CVD risk era.^30^ ^31^ However, this approach would not necessarily result in the greatest benefits and is not justifiable based on the available evidence. Despite this, there is evidence that this approach prevails in some areas. After adjusting for other CVD risk factors, lipid profile has been found to predict statin initiation in a US study^32^ and a randomised theoretical experiment in Australia highlighted clinicians’ preferences for managing individual risk factors over absolute risk.^33^ To further compound the problem, patients find it difficult to make decisions based on future risks and tend to preferentially focus on cholesterol levels when considering taking statins.^34^ ^35^

In 2014, guidelines for England and Wales on lipid modification reduced the predicted risk threshold at which patients should be offered statins from a 20% 10-year risk to 10%.^5^ ^7^ This recommendation was met with considerable concern from the medical community that it would lead to widespread overprescribing of statins with little clinical benefit but considerable potential for harm.^36^ ^37^ This widely publicised criticism may have increased concerns that risk scoring leads to oversimplification of clinical decisions and overprescribing and may have, perversely, decreased the use of risk scoring and reinforced the use of lipid levels to guide decision-making. There is some evidence to suggest there was a decrease in statin initiations around this time due to the negative media attention.^38^

This research will investigate the relationship between lipid profile, QRISK2 score and subsequent initiation of statins by primary care clinicians. This will give an indication of the patient factors that are influencing the decisions clinicians make in routine practice when considering the use of statins and how this has changed in response to external factors.

The consistent use of risk scoring can result in more targeted prescribing and fewer patients being prescribed medication overall^39^ which maximises the efficiency and cost-effectiveness of treatment at a population level.

## Aims and objectives

*Aim*: To establish whether UK primary care patients are initiated on statins based more on their lipid levels or on their predicted CVD risk and if this has changed over time.

*Objectives*:

For primary prevention of CVD

1. To evaluate how lipid levels and CVD risk independently predict subsequent prescribing of statins.
2. To determine if the relationship between lipid levels, QRISK2 score and statin prescribing has changed following recent guideline changes.
3. To investigate the contribution that calculating a QRISK2 score has on the probability of initiating statin therapy.
4. To investigate interprescriber variation when initiating statins.

## Methods and analysis

### Study design

A historical open cohort study of patients who were eligible for statin therapy for the primary prevention of CVD.

### Data source

Data will be extracted from anonymised primary care records from practices in England and Wales who contribute to The Health Improvement Network (THIN) database. The THIN database contains routine patient data from over 500 UK general practices which are generalisable to the UK population.^40^ THIN has been used in previous studies to validate QRISK2 and it was shown that the discrimination statistics in THIN are as good as those for the original QRISK2 cohort.^41^ ^42^ Practices that contribute to THIN use the Vision (In Practice Systems) electronic patient records system. Clinical data are coded using Read code clinical classification V.2^43^ and drug codes are used which correspond to the British National Formulary (BNF).^44^ The clinical codes used are available in online supplementary appendix I and the drug codes are available in online supplementary appendix II.

10.1136/bmjopen-2016-013120.supp1 supplementary appendix I

10.1136/bmjopen-2016-013120.supp2 supplementary appendix II

All data are anonymised and will be stored on secure severs at the University of Birmingham and accessed only through password-protected systems.

### Population

Patients will be eligible for inclusion if they are aged between 40 and 84 years inclusive (the age range recommended by the National Institute for Health and Care Excellence (NICE) for risk assessment^5^) with no previous diagnosis of CVD and at least one lipid result coded in the study period. The date of the first coded lipid result in the study period will be considered the index date. Patients must not have been prescribed a statin prior to the index date.

In order to maintain a stable cohort of practices, only THIN practices in which there are data available for the whole study period will be included. The study period will run from the beginning of 2012 to the end of 2016. Patients will be eligible for inclusion from the earliest of the following dates: study start date, acceptable mortality reporting date (which ensures that patient deaths and deregistrations are being recorded consistently^45^), Vision date plus 1 year, registration date plus 1 year (to ensure time for baseline data to be recorded), and age 40; until the earliest of the following dates: age 85, study end date, CVD diagnosis, other excluding diagnosis (as below), statin initiation or recording of a contraindication for the prescribing of statins. Patients will also be excluded if they left the database within 60 days of the index date.

Patients will be excluded if they had pre-existing CVD, were pregnant, had a contraindication to statin therapy (including allergies and drug interactions), were already being prescribed a statin (or other lipid-lowering therapy) or had ever been prescribed a statin in the past. Patients will be considered to have pre-existing CVD if they had a diagnosis of myocardial infarction, angina, peripheral vascular disease or stroke/transient ischaemic attack. Patients with type 1 diabetes mellitus, a diagnosis of chronic kidney disease stages 3–5 and patients diagnosed with or who were at risk of familial hypercholesterolaemia will also be excluded as statin use in these patients should not be based on CVD risk assessment. Patients at risk of familial hypercholesterolaemia will be defined as those with a total cholesterol of ≥9.0 mmol/L as this is the threshold for seeking specialist assessment defined in the NICE guidance.^5^

## Study variables

### Exposure variables

The exposure of interest is the undertaking of lipid testing. Once a patient has their lipids recorded, this signifies that a clinician has made a decision about the significance of the lipid levels and will act on this. Individual patients may have multiple lipid results recorded over the study period. Assuming the exclusion criteria are not met, each lipid result will be considered as a new exposure so long as it is not within 60 days of the last recorded lipid level. The number of previous lipid results will be included in predictive modelling.

### Outcome variables

The outcome will be the prescription of a statin within 60 days of a set of lipid results.

### Predictor variables

Sociodemographic details will be obtained for each patient: age, sex, ethnicity, Townsend quintile and rurality indicator (see online supplementary appendix III). General practice and clinician ID will also be recorded.

10.1136/bmjopen-2016-013120.supp3 supplementary appendix III

Patient variables will include the cardiovascular risk factors required to calculate QRISK2; age, sex, ethnicity, postcode, smoking status, diabetes status, family history of CVD, CKD (stage 4 or 5), atrial fibrillation, hypertension treatment, rheumatoid arthritis, lipids, blood pressure and body mass index (BMI). Other clinical data that may affect the decision to prescribe statins will also be collected comprising of; liver transaminase levels, systemic inflammatory conditions, severe mental health conditions and HIV.

Data on medication prescriptions will be extracted. Patients who received a prescription for antihypertensive medication up to 180 days prior to the index date will be considered to have been on treatment for hypertension. Prescriptions of antipsychotics, corticosteroids and immunosuppressant medication will be recorded as these may change the threshold for prescribing lipid-lowering medication.

All included patients will have a QRISK2 score calculated post hoc from the available data. In addition, any coded QRISK2 score prior to and including the follow-up period will be extracted.

The date when predictor variable data are collected will vary depending on whether or not the patient has a QRISK2 score coded during the follow-up period. For patients with a coded QRISK2 score on the index date or in the follow-up period, the date of QRISK2 recording will be used as the date for collecting data on predictor variables and the most recent data (including the date of QRISK2 coding) will be recorded. If more than one QRISK2 score is coded in the follow-up period, the latest date will be used for data collection to account for any variables that were updated during the follow-up period. For patients who do not have a coded QRISK2 score, the included data will be the most recent recorded before and including the end of the 60-day follow-up period.

### Missing data and extreme values

Missing data are anticipated in some variables; notably BMI, blood pressure and smoking status. If comorbidities are missing they will be assumed to be absent. Missing values arise twice: first when calculating the QRISK2 score post hoc, and second when constructing the multivariable model. These citations will be accounted for differently.

Where data are missing from variables used in QRISK2 calculations, the imputed values used by the QRISK2 calculator will be used.^46^ In the multivariable model, all variables will be categorical and a ‘missing’ category will be used to account for the possibility that missing data are associated with the initiation of statins. Biologically implausible values for blood pressure, lipids, liver transaminases, height and weight will be identified and excluded.

## Analysis

Continuous variables will be categorised. Calculated and coded QRISK2 scores will be categorised according to risk bands (<10%, 10–14.9%, 15–19.9% and ≥20%). Age will be categorised into four groups (40–49, 50–59, 60–69 and ≥70). Total cholesterol (TC) will be categorised into three bands (<5.0, 5.0–6.9 and ≥7.0 mmol/L), and high-density lipoprotein (HDL) cholesterol into four bands (≤1.2, 1.3–1.4, 1.5–1.7 and ≥1.8 mmol/L) as these cut-offs have been used previously in the literature.^22^ Systolic blood pressure will be split into three groups corresponding to normotensive, stage 1 hypertension and stage 2 hypertension (<140, 140–159 and ≥160 mm Hg).^6^

The cohort will be described and prescribing rates and QRISK2 score trends will be established.

The primary analysis will consist of predictive modelling using multivariate logistic regression (variables summarised in table 1). Clustering of responses within individual general practitioner (GP) practices will be included in the model. Variation throughout the year due to the seasonality imposed by the Quality and Outcomes Framework (QOF) targets will be accounted for by adding the year quarter as a variable in the model. Assuming a minimum sample size for analysis of 10 per degree of freedom,^47^ and 57 degrees of freedom in the variables, a minimum sample of 570 patients with a lipid profile and subsequent statin initiation should easily be obtained.

**Table 1:** Variables used in predictive modelling

Variable	Categories
Total cholesterol	<5.0, 5.0–6.9, ≥7.0
TC/HDL ratio	≤1.2, 1.3–1.4, 1.5–1.7, ≥1.8
Prior cholesterol measurements	0, 1–2, 3–4, ≥5
Calculated QRISK2	<10%, 10–14.9%, 15–19.9%, ≥20%
Coded QRISK2	<10%, 10–14.9%, 15–19.9%, ≥20%, missing
Systolic BP	<140, 140–159, ≥160, missing
Age	40–49, 50–59, 60–69, ≥70
Sex	Male, female
Ethnicity	White, Asian, black, other, missing
Townsend deprivation quintile	1st, 2nd, 3rd, 4th, 5th, missing
Urban/rural score	Urban, rural, missing
BMI	<20, 20–24.9, 25–29.9, 30–34.9, 35+, missing
Smoking status	Current smoker, ex-smoker, non-smoker, missing
Liver transaminases	Normal, abnormal, missing
Diabetes mellitus	Yes, no
Family history of CVD	Yes, no
Atrial fibrillation	Yes, no
Rheumatoid arthritis	Yes, no
Hypertension	Yes, no
Chronic kidney disease	Yes, no
HIV	Yes, no
Severe enduring mental illness*	Yes, no
Inflammatory conditions†	Yes, no
Antipsychotic medication	Yes, no
Long-term corticosteroids	Yes, no
Immunosuppressant medication	Yes, no
Year quartile	1st, 2nd, 3rd, 4th

*Schizophrenia, bipolar affective disorder, mania or psychosis (unspecified), psychotic depression.

†Systemic lupus erythematosus, scleroderma, Bechet's syndrome, other inflammatory arthritis.

BMI, body mass index; BP, blood pressure; CVD, cardiovascular disease; HDL, high-density lipoprotein.

The potential cohort size can be estimated from the literature. Feasibility data from the THIN database identified over 360 000 patients over 2 years who were eligible for statin treatment for the primary prevention of CVD and had a lipid result and blood pressure coded on their medical record.^22^ Over a 5-year period we should expect to identify over double this number. Another cohort identified just over two million patients in the THIN database in the age range where NHS health checks are offered.^48^ Given that every patient who attends an NHS health check should have a lipid test undertaken and 20% of 40–75 years old are invited to have a health check each year, with ∼50% taking up this offer.^49^ This would generate around 200 000 coded lipid results in the target population annually.

The accuracy of the post hoc QRISK2 calculations will be tested through comparisons with corresponding coded QRISK2 scores in patients where these are available.

Additional modelling will also be undertaken to consider whether having a QRISK2 score coded on the medical record affects the probability of having a statin prescribed after adjustment for the level of CVD risk. Secondary analysis will also establish whether the threshold for initiating statins has changed over the course of the study period. The mean lipid levels and QRISK2 scores (both coded and calculated post hoc) of those initiated on statins and those not initiated on statins will be calculated each year to reveal any trends in the thresholds for initiation.

Finally, prescriber-level data will be analysed to establish whether individuals tend to be influenced more by lipid levels or risk score when initiating stains and whether this behaviour is consistent over time. A score that illustrates the propensity to prescribe based on lipid levels will be calculated for individual clinicians. This will be generated by comparing the number of statin initiations to patients with a total cholesterol of ≥6.5 mmol/L with the number of statin initiations to patients with a total cholesterol of <6.5 mmol/L. Only clinicians who initiated 10 or more statin prescriptions over the study period will be analysed as scores for clinicians with fewer initiations may not be representative of their behaviour. The distribution of these scores will be described. For clinicians who initiated statins in every year of the study, their score will be calculated for the 2-year periods 2012–2013 and 2015–2016. The scores for each period will be split into quintiles. The tendency for clinicians to stay in the same quintile, or move quintile, will then be described.

## Potential limitations

The use of electronic patient records, while excellent for allowing access to large quantities of real-life data, does not allow complete understanding of the decision-making process. We will not be able to explore why a patient with a particular lipid profile does or does not receive a prescription for a statin. We cannot gain insight into what discussion took place as to the risk and benefits of treatment or even whether the clinician discussed the lipid result and CVD risk with the patient. It is also difficult to know to what extent clinicians are using QRISK2 scores in their decision-making process because, as only coded data are available, QRISK2 score may be calculated but not recorded in GP records. The Vision patient record system automatically displays a CVD risk score for patients who may benefit from CVD risk reduction^46^ and the clinician could act on this information without it being coded. This would result in patients being prescribed a statin based on their CVD risk without this being apparent through the coded information.

It is also possible that some patients will have their lipid levels checked outside of the primary care system (either in secondary care or the private sector) and may be started on statins by their GP without the lipid levels being recorded in primary care. These patients would not be included in this cohort. However, it is unlikely that this will represent a significant proportion of patients initiated on statins. Similarly, a small proportion of patients may be initiated on statins by secondary care or private clinicians and the prescription continued by their GP. In this scenario, the GP may not be involved in the decision-making process but this would not be identified from the data. Again, this is likely to be a very small proportion of the total statin initiations in the UK and is not of central interest as the decision to prescribe was made outside the primary care setting and is, therefore, unlikely to affect the validity of our conclusions.

In conclusion, while the methodology will not facilitate complete understanding of the individual decision-making consultations, it will provide new and valuable insight into the way that lipid levels and cardiovascular risk are currently being used in primary care to inform the decision to prescribe or offer statins. We will be able to establish whether the use of risk scoring is resulting in more evidence-based prescribing and improve understanding of some of the factors affecting decision-making which may inform future policy and clinical guidance.

## Ethics

The THIN Data Collection Scheme was approved by the South-East Multicentre Research Ethics Committee (SE-MREC) in 2003.^50^ Use of the data for research purposes does not require further ethical review but must be approved by the independent Scientific Review Committee (SCR). The original protocol for this study and a subsequent amendment have been approved (16THIN009A1).

## Dissemination

The findings of this study will be published in a peer-reviewed journal and presented at appropriate national and international conferences. The results will also be disseminated through social media and through specialist interest groups.
