# Supplementary material for: Cohort study investigating the relationship between cholesterol, cardiovascular risk score and the prescribing of statins in UK primary care: study protocol
Source: BMJ Open. 2016 Nov 17;6(11):e013120. doi: 10.1136/bmjopen-2016-013120 (PMC5128938; doi:10.1136/bmjopen-2016-013120)
Supplement: supplementary appendix I [file bmjopen-2016-013120supp_appendixI.pdf]

Ischaemic Heart Disease

medcode	description
G3...00	Ischaemic heart disease
G3...11	Arteriosclerotic heart disease
G3...12	Atherosclerotic heart disease
G3...13	IHD - Ischaemic heart disease
G30..00	Acute myocardial infarction
G30..11	Attack - heart
G30..12	Coronary thrombosis
G30..13	Cardiac rupture following myocardial infarction (MI)
G30..14	Heart attack
G30..15	MI - acute myocardial infarction
G30..16	Thrombosis - coronary
G30..17	Silent myocardial infarction
G300.00	Acute anterolateral infarction
G301.00	Other specified anterior myocardial infarction
G301000	Acute anteroapical infarction
G301100	Acute anteroseptal infarction
G301z00	Anterior myocardial infarction NOS
G302.00	Acute inferolateral infarction
G303.00	Acute inferoposterior infarction
G304.00	Posterior myocardial infarction NOS
G305.00	Lateral myocardial infarction NOS
G306.00	True posterior myocardial infarction
G307.00	Acute subendocardial infarction
G307000	Acute non-Q wave infarction
G307100	Acute non-ST segment elevation myocardial infarction
G308.00	Inferior myocardial infarction NOS
G309.00	Acute Q-wave infarct
G30B.00	Acute posterolateral myocardial infarction
G30X.00	Acute transmural myocardial infarction of unspecif site
G30X000	Acute ST segment elevation myocardial infarction
G30y.00	Other acute myocardial infarction
G30y000	Acute atrial infarction
G30y100	Acute papillary muscle infarction
G30y200	Acute septal infarction
G30yz00	Other acute myocardial infarction NOS
G30z.00	Acute myocardial infarction NOS
G31..00	Other acute and subacute ischaemic heart disease
G311.00	Preinfarction syndrome
G311.11	Crescendo angina
G311.12	Impending infarction
G311.13	Unstable angina
G311.14	Angina at rest
G311000	Myocardial infarction aborted
G311011	MI - myocardial infarction aborted
G311100	Unstable angina
G311200	Angina at rest
G311300	Refractory angina
G311400	Worsening angina

G311500	Acute coronary syndrome
G311z00	Preinfarction syndrome NOS
G312.00	Coronary thrombosis not resulting in myocardial infarction
G31y.00	Other acute and subacute ischaemic heart disease
G31y000	Acute coronary insufficiency
G31y100	Microinfarction of heart
G31y200	Subendocardial ischaemia
G31y300	Transient myocardial ischaemia
G31yz00	Other acute and subacute ischaemic heart disease NOS
G32..00	Old myocardial infarction
G32..11	Healed myocardial infarction
G32..12	Personal history of myocardial infarction
G33..00	Angina pectoris
G330.00	Angina decubitus
G330000	Nocturnal angina
G330z00	Angina decubitus NOS
G33z.00	Angina pectoris NOS
G33z000	Status anginosus
G33z100	Stenocardia
G33z200	Syncope anginosa
G33z300	Angina on effort
G33z400	Ischaemic chest pain
G33z500	Post infarct angina
G33z600	New onset angina
G33z700	Stable angina
G33zz00	Angina pectoris NOS
G34..00	Other chronic ischaemic heart disease
G340.00	Coronary atherosclerosis
G340.11	Triple vessel disease of the heart
G340.12	Coronary artery disease
G340000	Single coronary vessel disease
G340100	Double coronary vessel disease
G342.00	Atherosclerotic cardiovascular disease
G343.00	Ischaemic cardiomyopathy
G344.00	Silent myocardial ischaemia
G34y.00	Other specified chronic ischaemic heart disease
G34y000	Chronic coronary insufficiency
G34y100	Chronic myocardial ischaemia
G34yz00	Other specified chronic ischaemic heart disease NOS
G34z.00	Other chronic ischaemic heart disease NOS
G34z000	Asymptomatic coronary heart disease
G35..00	Subsequent myocardial infarction
G350.00	Subsequent myocardial infarction of anterior wall
G351.00	Subsequent myocardial infarction of inferior wall
G353.00	Subsequent myocardial infarction of other sites
G35X.00	Subsequent myocardial infarction of unspecified site
G38..00	Postoperative myocardial infarction
G380.00	Postoperative transmural myocardial infarction anterior wall
G381.00	Postoperative transmural myocardial infarction inferior wall
G382.00	Postoperative transmural myocardial infarction other sites

G383.00	Postoperative transmural myocardial infarction unspec site
G384.00	Postoperative subendocardial myocardial infarction
G38z.00	Postoperative myocardial infarction, unspecified
G39..00	Coronary microvascular disease
G3y..00	Other specified ischaemic heart disease
G3z..00	Ischaemic heart disease NOS
Gyu3.00	[X]Ischaemic heart diseases
Gyu3000	[X]Other forms of angina pectoris
Gyu3200	[X]Other forms of acute ischaemic heart disease
Gyu3300	[X]Other forms of chronic ischaemic heart disease
Gyu3400	[X]Acute transmural myocardial infarction of unspecif site
Gyu3500	[X]Subsequent myocardial infarction of other sites
Gyu3600	[X]Subsequent myocardial infarction of unspecified site

Stroke

medcode	description
14A7.00	H/O: CVA/stroke
14A7.12	H/O: stroke
14AF.00	H/O sub-arachnoid haemorrhage
14AK.00	H/O: Stroke in last year
1M4..00	Central post-stroke pain
661M700	Stroke self-management plan agreed
661N700	Stroke self-management plan review
662e.00	Stroke/CVA annual review
662e.11	Stroke annual review
662M.00	Stroke monitoring
662M100	Stroke 6 month review
662M200	Stroke initial post discharge review
662o.00	Haemorrhagic stroke monitoring
7P24200	Delivery of rehabilitation for stroke
8Hd6.00	Admission to stroke unit
8HHM.00	Ref to multidisciplinary stroke function improvement service
8HTQ.00	Referral to stroke clinic
8IEC.00	Ref multidisciplinary stroke function improvement declined
9Om..00	Stroke/transient ischaemic attack monitoring administration
9Om0.00	Stroke/transient ischaemic attack monitoring first letter
9Om1.00	Stroke/transient ischaemic attack monitoring second letter
9Om2.00	Stroke/transient ischaemic attack monitoring third letter
9Om3.00	Stroke/transient ischaemic attack monitoring verbal invitati
9Om4.00	Stroke/transient ischaemic attack monitoring telephone invte
G680.00	Sequelae of subarachnoid haemorrhage
G681.00	Sequelae of intracerebral haemorrhage
G682.00	Sequelae of other nontraumatic intracranial haemorrhage
G683.00	Sequelae of cerebral infarction
G68W.00	Sequelae/other + unspecified cerebrovascular diseases
G68X.00	Sequelae of stroke,not specfd as h'morrhage or infarction
Gyu6B00	[X]Sequelae of other nontraumatic intracranial haemorrhage
Gyu6C00	[X]Sequelae of stroke,not specfd as h'morrhage or infarction
Gyu6D00	[X]Sequelae/other + unspecified cerebrovascular diseases
ZLEP.00	Discharge from stroke serv
ZV12511	[V]Personal history of stroke
G61..00	Intracerebral haemorrhage
G61..11	CVA - cerebrovascular accid due to intracerebral haemorrhage
G61..12	Stroke due to intracerebral haemorrhage
G610.00	Cortical haemorrhage
G611.00	Internal capsule haemorrhage
G612.00	Basal nucleus haemorrhage
G613.00	Cerebellar haemorrhage
G614.00	Pontine haemorrhage
G615.00	Bulbar haemorrhage
G616.00	External capsule haemorrhage
G618.00	Intracerebral haemorrhage, multiple localized
G619.00	Lobar cerebral haemorrhage
G61X.00	Intracerebral haemorrhage in hemisphere, unspecified

G61X000	Left sided intracerebral haemorrhage, unspecified
G61X100	Right sided intracerebral haemorrhage, unspecified
G61z.00	Intracerebral haemorrhage NOS
G63y000	Cerebral infarct due to thrombosis of precerebral arteries
G63y100	Cerebral infarction due to embolism of precerebral arteries
G64..00	Cerebral arterial occlusion
G64..11	CVA - cerebral artery occlusion
G64..12	Infarction - cerebral
G64..13	Stroke due to cerebral arterial occlusion
G640.00	Cerebral thrombosis
G640000	Cerebral infarction due to thrombosis of cerebral arteries
G641.00	Cerebral embolism
G641.11	Cerebral embolus
G641000	Cerebral infarction due to embolism of cerebral arteries
G64z.00	Cerebral infarction NOS
G64z.11	Brainstem infarction NOS
G64z.12	Cerebellar infarction
G64z000	Brainstem infarction
G64z100	Wallenberg syndrome
G64z111	Lateral medullary syndrome
G64z200	Left sided cerebral infarction
G64z300	Right sided cerebral infarction
G64z400	Infarction of basal ganglia
G66..00	Stroke and cerebrovascular accident unspecified
G66..11	CVA unspecified
G66..12	Stroke unspecified
G66..13	CVA - Cerebrovascular accident unspecified
G660.00	Middle cerebral artery syndrome
G661.00	Anterior cerebral artery syndrome
G662.00	Posterior cerebral artery syndrome
G663.00	Brain stem stroke syndrome
G664.00	Cerebellar stroke syndrome
G665.00	Pure motor lacunar syndrome
G666.00	Pure sensory lacunar syndrome
G667.00	Left sided CVA
G668.00	Right sided CVA
G676000	Cereb infarct due cerebral venous thrombosis, nonpyogenic
G6W..00	Cereb infarct due unsp occlus/stenos precerebr arteries
G6X..00	Cerebrl infarctn due/unspcf occlusn or sten/cerebrl artrs
G6y..00	Other specified cerebrovascular disease
G6z..00	Cerebrovascular disease NOS
Gyu6200	[X]Other intracerebral haemorrhage
Gyu6300	[X]Cerebrl infarctn due/unspcf occlusn or sten/cerebrl artrs
Gyu6400	[X]Other cerebral infarction
Gyu6500	[X]Occlusion and stenosis of other precerebral arteries
Gyu6600	[X]Occlusion and stenosis of other cerebral arteries
Gyu6F00	[X]Intracerebral haemorrhage in hemisphere, unspecified
Gyu6G00	[X]Cereb infarct due unsp occlus/stenos precerebr arteries
G619.00	Lobar cerebral haemorrhage

TIA

medcode	description
Fyu5500	[X]Other transnt cerebral ischaemic attacks+related syndroms
G65..00	Transient cerebral ischaemia
G65..11	Drop attack
G65..12	Transient ischaemic attack
G65..13	Vertebro-basilar insufficiency
G650.00	Basilar artery syndrome
G650.11	Insufficiency - basilar artery
G651.00	Vertebral artery syndrome
G651000	Vertebro-basilar artery syndrome
G652.00	Subclavian steal syndrome
G653.00	Carotid artery syndrome hemispheric
G654.00	Multiple and bilateral precerebral artery syndromes
G656.00	Vertebrobasilar insufficiency
G657.00	Carotid territory transient ischaemic attack
G65y.00	Other transient cerebral ischaemia
G65z.00	Transient cerebral ischaemia NOS
G65z000	Impending cerebral ischaemia
G65z100	Intermittent cerebral ischaemia
G65zz00	Transient cerebral ischaemia NOS
ZV12D00	[V]Personal history of transient ischaemic attack

Peripheral Vascular Disease

medcode	description
G73..00	Other peripheral vascular diseas
G73..11	Peripheral ischaemic vascular disease
G73..12	Ischaemia of legs
G73..13	Peripheral ischaemia
G73z.00	Peripheral vascular disease NOS
G73z000	Intermittent claudication
G73z011	Claudication
G73z012	Vascular claudication
G73zz00	Peripheral vascular disease NOS
Gyu7400	[X]Other specified peripheral vascular diseases
G734.00	Peripheral arterial disease
G73y.00	Other specified peripheral vascular disease

Hypertension

medcode	description
G2...00	Hypertensive disease
G2...11	BP - hypertensive disease
G2...11	BP - hypertensive disease
G20..00	Essential hypertension
G20..11	High blood pressure
G20..12	Primary hypertension
G200.00	Malignant essential hypertension
G201.00	Benign essential hypertension
G202.00	Systolic hypertension
G203.00	Diastolic hypertension
G20z.00	Essential hypertension NOS
G20z.11	Hypertension NOS
G21..00	Hypertensive heart disease
G210.00	Malignant hypertensive heart disease
G210000	Malignant hypertensive heart disease without CCF
G210100	Malignant hypertensive heart disease with CCF
G210z00	Malignant hypertensive heart disease NOS
G211.00	Benign hypertensive heart disease
G211000	Benign hypertensive heart disease without CCF
G211100	Benign hypertensive heart disease with CCF
G21z.00	Hypertensive heart disease NOS
G21z000	Hypertensive heart disease NOS without CCF
G21z011	Cardiomegaly - hypertensive
G21z100	Hypertensive heart disease NOS with CCF
G21zz00	Hypertensive heart disease NOS
G22..00	Hypertensive renal disease
G220.00	Malignant hypertensive renal disease
G221.00	Benign hypertensive renal disease
G222.00	Hypertensive renal disease with renal failure
G22z.00	Hypertensive renal disease NOS
G22z.11	Renal hypertension
G24..00	Secondary hypertension
G240.00	Secondary malignant hypertension
G240000	Secondary malignant renovascular hypertension
G240z00	Secondary malignant hypertension NOS
G241.00	Secondary benign hypertension
G241000	Secondary benign renovascular hypertension
G241z00	Secondary benign hypertension NOS
G244.00	Hypertension secondary to endocrine disorders
G24z.00	Secondary hypertension NOS
G24z000	Secondary renovascular hypertension NOS
G24zz00	Secondary hypertension NOS
G25..00	Stage 1 hypertension (NICE - Nat Ins for Hth Clin Excl 2011)
G25..11	Stage 1 hypertension
G250.00	Stage 1 hyperten (NICE 2011) without evidnce end organ damge
G251.00	Stage 1 hyperten (NICE 2011) with evidnce end organ damge
G26..00	Severe hypertension (Nat Inst for Health Clinical Ex 2011)
G26..11	Severe hypertension

G28..00	Stage 2 hypertension (NICE - Nat Ins for Hth Clin Excl 2011)
G2y..00	Other specified hypertensive disease
G2z..00	Hypertensive disease NOS
Gyu2.00	[X]Hypertensive diseases
Gyu2000	[X]Other secondary hypertension
Gyu2100	[X]Hypertension secondary to other renal disorders

CKD 3-5

medcode	description
1Z12.00	Chronic kidney disease stage 3
1Z13.00	Chronic kidney disease stage 4
1Z14.00	Chronic kidney disease stage 5
1Z15.00	Chronic kidney disease stage 3A
1Z16.00	Chronic kidney disease stage 3B
1Z1B.00	Chronic kidney disease stage 3 with proteinuria
1Z1B.11	CKD stage 3 with proteinuria
1Z1C.00	Chronic kidney disease stage 3 without proteinuria
1Z1C.11	CKD stage 3 without proteinuria
1Z1D.00	Chronic kidney disease stage 3A with proteinuria
1Z1D.11	CKD stage 3A with proteinuria
1Z1E.00	Chronic kidney disease stage 3A without proteinuria
1Z1E.11	CKD stage 3A without proteinuria
1Z1F.00	Chronic kidney disease stage 3B with proteinuria
1Z1F.11	CKD stage 3B with proteinuria
1Z1G.00	Chronic kidney disease stage 3B without proteinuria
1Z1G.11	CKD stage 3B without proteinuria
1Z1H.00	Chronic kidney disease stage 4 with proteinuria
1Z1H.11	CKD stage 4 with proteinuria
1Z1J.00	Chronic kidney disease stage 4 without proteinuria
1Z1J.11	CKD stage 4 without proteinuria
1Z1K.00	Chronic kidney disease stage 5 with proteinuria
1Z1K.11	CKD stage 5 with proteinuria
1Z1L.00	Chronic kidney disease stage 5 without proteinuria
1Z1L.11	CKD stage 5 without proteinuria
K053.00	Chronic kidney disease stage 3
K054.00	Chronic kidney disease stage 4
K055.00	Chronic kidney disease stage 5
K05..00	Chronic renal failure
K050.00	End stage renal failure
K05..12	End stage renal failure
K0D..00	End-stage renal disease

Atrial Fibrillation

medcode	description
14AN.00	h/o: atrial fibrillation
662S.00	atrial fibrillation monitoring
6A9..00	atrial fibrillation annual review
7936A00	implant intravenous pacemaker for atrial fibrillation
8CMW200	atrial fibrillation care pathway
9Os..00	atrial fibrillation monitoring administration
9Os0.00	atrial fibrillation monitoring first letter
9Os1.00	atrial fibrillation monitoring second letter
9Os2.00	atrial fibrillation monitoring third letter
9Os3.00	atrial fibrillation monitoring verbal invite
9Os4.00	atrial fibrillation monitoring telephone invite
9hF..00	exception reporting: atrial fibrillation quality indicators
9hF1.00	excepted from atrial fibrillation qual indic: inform dissent
G573.00	Atrial fibrillation and flutter
G573000	Atrial fibrillation
G573200	Paroxysmal atrial fibrillation
G573300	Non-rheumatic atrial fibrillation
G573400	Permanent atrial fibrillation
G573500	Persistent atrial fibrillation
G573z00	Atrial fibrillation and flutter NOS

Familial Hypercholesterolaemia

medcode	description
1W2..00	probable familial hypercholesterolaemia
C320.11	familial hypercholesterolaemia
C320000	familial hypercholesterolaemia
C320100	hyperbetalipoproteinaemia
C320400	fredrickson's hyperlipoproteinaemia, type IIa
C320500	fam defect apolipoprot B-100

Liver disease

medcode	description
C310400	Glycogenosis with hepatic cirrhosis
C350012	Pigmentary cirrhosis of liver
C370800	Cystic fibrosis related cirrhosis
G852200	Oesophageal varices in cirrhosis of the liver
G852300	Oesophageal varices in alcoholic cirrhosis of the liver
J61..00	Cirrhosis and chronic liver disease
J612.00	Alcoholic cirrhosis of liver
J612.11	Florid cirrhosis
J612.12	Laennec's cirrhosis
J612000	Alcoholic fibrosis and sclerosis of liver
J615.11	Portal cirrhosis
J615000	Unilobular portal cirrhosis
J615100	Multilobular portal cirrhosis
J615111	Postnecrotic cirrhosis of liver
J615200	Mixed portal cirrhosis
J615300	Diffuse nodular cirrhosis
J615400	Fatty portal cirrhosis
J615500	Hypertrophic portal cirrhosis
J615600	Capsular portal cirrhosis
J615700	Cardiac portal cirrhosis
J615711	Congestive cirrhosis
J615800	Juvenile portal cirrhosis
J615811	Childhood function cirrhosis
J615812	Indian childhood cirrhosis
J615900	Pigmentary portal cirrhosis
J615A00	Pipe-stem portal cirrhosis
J615B00	Toxic portal cirrhosis
J615C00	Xanthomatous portal cirrhosis
J615D00	Bacterial portal cirrhosis
J615E00	Cardituberculous cirrhosis
J615F00	Syphilitic portal cirrhosis
J615G00	Zooparasitic portal cirrhosis
J615H00	Infectious cirrhosis NOS
J615y00	Portal cirrhosis unspecified
J615z00	Non-alcoholic cirrhosis NOS
J615z11	Macronodular cirrhosis of liver
J615z12	Cryptogenic cirrhosis of liver
J615z13	Cirrhosis of liver NOS
J615z14	Laennec's cirrhosis, non-alcoholic
J615z15	Hepatic fibrosis
J616.00	Biliary cirrhosis
J616000	Primary biliary cirrhosis
J616100	Secondary biliary cirrhosis
J616200	Biliary cirrhosis of children
J616z00	Biliary cirrhosis NOS
J635600	Toxic liver disease with fibrosis and cirrhosis of liver
Jyu7100	[X]Other and unspecified cirrhosis of liver

T1DM

medcode	description
C10E.00	Type 1 diabetes mellitus
C10E.11	Type I diabetes mellitus
C10E.12	Insulin dependent diabetes mellitus
C10E000	Type 1 diabetes mellitus with renal complications
C10E011	Type I diabetes mellitus with renal complications
C10E012	Insulin-dependent diabetes mellitus with renal complications
C10E100	Type 1 diabetes mellitus with ophthalmic complications
C10E111	Type I diabetes mellitus with ophthalmic complications
C10E112	Insulin-dependent diabetes mellitus with ophthalmic comps
C10E200	Type 1 diabetes mellitus with neurological complications
C10E211	Type I diabetes mellitus with neurological complications
C10E212	Insulin-dependent diabetes mellitus with neurological comps
C10E300	Type 1 diabetes mellitus with multiple complications
C10E311	Type I diabetes mellitus with multiple complications
C10E312	Insulin dependent diabetes mellitus with multiple complicat
C10E400	Unstable type 1 diabetes mellitus
C10E411	Unstable type I diabetes mellitus
C10E412	Unstable insulin dependent diabetes mellitus
C10E500	Type 1 diabetes mellitus with ulcer
C10E511	Type I diabetes mellitus with ulcer
C10E512	Insulin dependent diabetes mellitus with ulcer
C10E600	Type 1 diabetes mellitus with gangrene
C10E611	Type I diabetes mellitus with gangrene
C10E612	Insulin dependent diabetes mellitus with gangrene
C10E700	Type 1 diabetes mellitus with retinopathy
C10E711	Type I diabetes mellitus with retinopathy
C10E712	Insulin dependent diabetes mellitus with retinopathy
C10E800	Type 1 diabetes mellitus - poor control
C10E811	Type I diabetes mellitus - poor control
C10E812	Insulin dependent diabetes mellitus - poor control
C10E900	Type 1 diabetes mellitus maturity onset
C10E911	Type I diabetes mellitus maturity onset
C10E912	Insulin dependent diabetes maturity onset
C10EA00	Type 1 diabetes mellitus without complication
C10EA11	Type I diabetes mellitus without complication
C10EA12	Insulin-dependent diabetes without complication
C10EB00	Type 1 diabetes mellitus with mononeuropathy
C10EB11	Type I diabetes mellitus with mononeuropathy
C10EB12	Insulin dependent diabetes mellitus with mononeuropathy
C10EC00	Type 1 diabetes mellitus with polyneuropathy
C10EC11	Type I diabetes mellitus with polyneuropathy
C10EC12	Insulin dependent diabetes mellitus with polyneuropathy
C10ED00	Type 1 diabetes mellitus with nephropathy
C10ED11	Type I diabetes mellitus with nephropathy
C10ED12	Insulin dependent diabetes mellitus with nephropathy
C10EE00	Type 1 diabetes mellitus with hypoglycaemic coma
C10EE11	Type I diabetes mellitus with hypoglycaemic coma
C10EE12	Insulin dependent diabetes mellitus with hypoglycaemic coma

C10EF00	Type 1 diabetes mellitus with diabetic cataract
C10EF11	Type I diabetes mellitus with diabetic cataract
C10EF12	Insulin dependent diabetes mellitus with diabetic cataract
C10EG00	Type 1 diabetes mellitus with peripheral angiopathy
C10EG11	Type I diabetes mellitus with peripheral angiopathy
C10EG12	Insulin dependent diab mell with peripheral angiopathy
C10EH00	Type 1 diabetes mellitus with arthropathy
C10EH11	Type I diabetes mellitus with arthropathy
C10EH12	Insulin dependent diabetes mellitus with arthropathy
C10EJ00	Type 1 diabetes mellitus with neuropathic arthropathy
C10EJ11	Type I diabetes mellitus with neuropathic arthropathy
C10EJ12	Insulin dependent diab mell with neuropathic arthropathy
C10EK00	Type 1 diabetes mellitus with persistent proteinuria
C10EK11	Type I diabetes mellitus with persistent proteinuria
C10EL00	Type 1 diabetes mellitus with persistent microalbuminuria
C10EL11	Type I diabetes mellitus with persistent microalbuminuria
C10EM00	Type 1 diabetes mellitus with ketoacidosis
C10EM11	Type I diabetes mellitus with ketoacidosis
C10EN00	Type 1 diabetes mellitus with ketoacidotic coma
C10EN11	Type I diabetes mellitus with ketoacidotic coma
C10EP00	Type 1 diabetes mellitus with exudative maculopathy
C10EP11	Type I diabetes mellitus with exudative maculopathy
C10EQ00	Type 1 diabetes mellitus with gastroparesis
C10EQ11	Type I diabetes mellitus with gastroparesis
C10ER00	Latent autoimmune diabetes mellitus in adult

T2DM

medcode	description
C10F.00	Type 2 diabetes mellitus
C10F.11	Type II diabetes mellitus
C10F000	Type 2 diabetes mellitus with renal complications
C10F011	Type II diabetes mellitus with renal complications
C10F100	Type 2 diabetes mellitus with ophthalmic complications
C10F111	Type II diabetes mellitus with ophthalmic complications
C10F200	Type 2 diabetes mellitus with neurological complications
C10F211	Type II diabetes mellitus with neurological complications
C10F300	Type 2 diabetes mellitus with multiple complications
C10F311	Type II diabetes mellitus with multiple complications
C10F400	Type 2 diabetes mellitus with ulcer
C10F411	Type II diabetes mellitus with ulcer
C10F500	Type 2 diabetes mellitus with gangrene
C10F511	Type II diabetes mellitus with gangrene
C10F600	Type 2 diabetes mellitus with retinopathy
C10F611	Type II diabetes mellitus with retinopathy
C10F700	Type 2 diabetes mellitus - poor control
C10F711	Type II diabetes mellitus - poor control
C10F811	Metabolic syndrome X
C10F900	Type 2 diabetes mellitus without complication
C10F911	Type II diabetes mellitus without complication
C10FA00	Type 2 diabetes mellitus with mononeuropathy
C10FA11	Type II diabetes mellitus with mononeuropathy
C10FB00	Type 2 diabetes mellitus with polyneuropathy
C10FB11	Type II diabetes mellitus with polyneuropathy
C10FC00	Type 2 diabetes mellitus with nephropathy
C10FC11	Type II diabetes mellitus with nephropathy
C10FD00	Type 2 diabetes mellitus with hypoglycaemic coma
C10FD11	Type II diabetes mellitus with hypoglycaemic coma
C10FE00	Type 2 diabetes mellitus with diabetic cataract
C10FE11	Type II diabetes mellitus with diabetic cataract
C10FF00	Type 2 diabetes mellitus with peripheral angiopathy
C10FF11	Type II diabetes mellitus with peripheral angiopathy
C10FG00	Type 2 diabetes mellitus with arthropathy
C10FG11	Type II diabetes mellitus with arthropathy
C10FH00	Type 2 diabetes mellitus with neuropathic arthropathy
C10FH11	Type II diabetes mellitus with neuropathic arthropathy
C10FJ00	Insulin treated Type 2 diabetes mellitus
C10FJ11	Insulin treated Type II diabetes mellitus
C10FK00	Hyperosmolar non-ketotic state in type 2 diabetes mellitus
C10FK11	Hyperosmolar non-ketotic state in type II diabetes mellitus
C10FL00	Type 2 diabetes mellitus with persistent proteinuria
C10FL11	Type II diabetes mellitus with persistent proteinuria
C10FM00	Type 2 diabetes mellitus with persistent microalbuminuria
C10FM11	Type II diabetes mellitus with persistent microalbuminuria
C10FN00	Type 2 diabetes mellitus with ketoacidosis
C10FN11	Type II diabetes mellitus with ketoacidosis
C10FP00	Type 2 diabetes mellitus with ketoacidotic coma

C10FP11	Type II diabetes mellitus with ketoacidotic coma
C10FQ00	Type 2 diabetes mellitus with exudative maculopathy
C10FQ11	Type II diabetes mellitus with exudative maculopathy
C10FR00	Type 2 diabetes mellitus with gastroparesis
C10FR11	Type II diabetes mellitus with gastroparesis
C10FS00	Maternally inherited diabetes mellitus

FH CVD

medcode	description
12C2.00	FH: Ischaemic heart dis. <60
12C2.11	FH: Myocardial infarction < 60
12C2.12	FH: MI- Myocardial infarct <60
12C2.13	FH: Angina < 60yrs
12CM.00	FH: Angina in 1st degree male relative <55 years
12CP.00	FH: Myocardial infarct in 1st degree male relative <55 years

Severe enduring mental illness

medcode	description
E10..00	Schizophrenic disorders
E100.00	Simple schizophrenia
E100.11	Schizophrenia simplex
E100000	Unspecified schizophrenia
E100100	Subchronic schizophrenia
E100200	Chronic schizophrenic
E100300	Acute exacerbation of subchronic schizophrenia
E100400	Acute exacerbation of chronic schizophrenia
E100500	Schizophrenia in remission
E100z00	Simple schizophrenia NOS
E101.00	Hebephrenic schizophrenia
E101000	Unspecified hebephrenic schizophrenia
E101100	Subchronic hebephrenic schizophrenia
E101200	Chronic hebephrenic schizophrenia
E101300	Acute exacerbation of subchronic hebephrenic schizophrenia
E101400	Acute exacerbation of chronic hebephrenic schizophrenia
E101500	Hebephrenic schizophrenia in remission
E101z00	Hebephrenic schizophrenia NOS
E102.00	Catatonic schizophrenia
E102000	Unspecified catatonic schizophrenia
E102100	Subchronic catatonic schizophrenia
E102200	Chronic catatonic schizophrenia
E102300	Acute exacerbation of subchronic catatonic schizophrenia
E102400	Acute exacerbation of chronic catatonic schizophrenia
E102500	Catatonic schizophrenia in remission
E102z00	Catatonic schizophrenia NOS
E103.00	Paranoid schizophrenia
E103000	Unspecified paranoid schizophrenia
E103100	Subchronic paranoid schizophrenia
E103200	Chronic paranoid schizophrenia
E103300	Acute exacerbation of subchronic paranoid schizophrenia
E103400	Acute exacerbation of chronic paranoid schizophrenia
E103500	Paranoid schizophrenia in remission
E103z00	Paranoid schizophrenia NOS
E104.00	Acute schizophrenic episode
E104.11	Oneirophrenia
E105.00	Latent schizophrenia
E105000	Unspecified latent schizophrenia
E105100	Subchronic latent schizophrenia
E105200	Chronic latent schizophrenia
E105300	Acute exacerbation of subchronic latent schizophrenia
E105400	Acute exacerbation of chronic latent schizophrenia
E105500	Latent schizophrenia in remission
E105z00	Latent schizophrenia NOS
E106.00	Residual schizophrenia
E106.11	Restzustand - schizophrenia
E107.00	Schizo-affective schizophrenia
E107.11	Cyclic schizophrenia

E107000	Unspecified schizo-affective schizophrenia
E107100	Subchronic schizo-affective schizophrenia
E107200	Chronic schizo-affective schizophrenia
E107300	Acute exacerbation subchronic schizo-affective schizophrenia
E107400	Acute exacerbation of chronic schizo-affective schizophrenia
E107500	Schizo-affective schizophrenia in remission
E107z00	Schizo-affective schizophrenia NOS
E10y.00	Other schizophrenia
E10y.11	Cenesthopathic schizophrenia
E10y000	Atypical schizophrenia
E10y100	Coenesthopathic schizophrenia
E10yz00	Other schizophrenia NOS
E10z.00	Schizophrenia NOS
E110.00	Manic disorder, single episode
E110.11	Hypomanic psychoses
E110000	Single manic episode, unspecified
E110100	Single manic episode, mild
E110200	Single manic episode, moderate
E110300	Single manic episode, severe without mention of psychosis
E110400	Single manic episode, severe, with psychosis
E110500	Single manic episode in partial or unspecified remission
E110600	Single manic episode in full remission
E110z00	Manic disorder, single episode NOS
E111.00	Recurrent manic episodes
E111000	Recurrent manic episodes, unspecified
E111100	Recurrent manic episodes, mild
E111200	Recurrent manic episodes, moderate
E111300	Recurrent manic episodes, severe without mention psychosis
E111400	Recurrent manic episodes, severe, with psychosis
E111500	Recurrent manic episodes, partial or unspecified remission
E111600	Recurrent manic episodes, in full remission
E111z00	Recurrent manic episode NOS
E112400	Single major depressive episode, severe, with psychosis
E113400	Recurrent major depressive episodes, severe, with psychosis
E114.00	Bipolar affective disorder, currently manic
E114.11	Manic-depressive - now manic
E114000	Bipolar affective disorder, currently manic, unspecified
E114100	Bipolar affective disorder, currently manic, mild
E114200	Bipolar affective disorder, currently manic, moderate
E114300	Bipolar affect disord, currently manic, severe, no psychosis
E114400	Bipolar affect disord, currently manic,severe with psychosis
E114500	Bipolar affect disord,currently manic, part/unspec remission
E114600	Bipolar affective disorder, currently manic, full remission
E114z00	Bipolar affective disorder, currently manic, NOS
E115.00	Bipolar affective disorder, currently depressed
E115.11	Manic-depressive - now depressed
E115000	Bipolar affective disorder, currently depressed, unspecified
E115100	Bipolar affective disorder, currently depressed, mild
E115200	Bipolar affective disorder, currently depressed, moderate
E115300	Bipolar affect disord, now depressed, severe, no psychosis

E115400	Bipolar affect disord, now depressed, severe with psychosis
E115500	Bipolar affect disord, now depressed, part/unspec remission
E115600	Bipolar affective disorder, now depressed, in full remission
E115z00	Bipolar affective disorder, currently depressed, NOS
E116.00	Mixed bipolar affective disorder
E116000	Mixed bipolar affective disorder, unspecified
E116100	Mixed bipolar affective disorder, mild
E116200	Mixed bipolar affective disorder, moderate
E116300	Mixed bipolar affective disorder, severe, without psychosis
E116400	Mixed bipolar affective disorder, severe, with psychosis
E116500	Mixed bipolar affective disorder, partial/unspec remission
E116600	Mixed bipolar affective disorder, in full remission
E116z00	Mixed bipolar affective disorder, NOS
E117.00	Unspecified bipolar affective disorder
E117000	Unspecified bipolar affective disorder, unspecified
E117100	Unspecified bipolar affective disorder, mild
E117200	Unspecified bipolar affective disorder, moderate
E117300	Unspecified bipolar affective disorder, severe, no psychosis
E117400	Unspecified bipolar affective disorder,severe with psychosis
E117500	Unspecified bipolar affect disord, partial/unspec remission
E117600	Unspecified bipolar affective disorder, in full remission
E117z00	Unspecified bipolar affective disorder, NOS
E11y.00	Other and unspecified manic-depressive psychoses
E11y000	Unspecified manic-depressive psychoses
E11y100	Atypical manic disorder
E11y300	Other mixed manic-depressive psychoses
E11yz00	Other and unspecified manic-depressive psychoses NOS
E11z.00	Other and unspecified affective psychoses
E11z000	Unspecified affective psychoses NOS
E11zz00	Other affective psychosis NOS
E12..00	Paranoid states
E120.00	Simple paranoid state
E121.00	Chronic paranoid psychosis
E121.11	Sander's disease
E122.00	Paraphrenia
E123.00	Shared paranoid disorder
E123.11	Folie a deux
E12y.00	Other paranoid states
E12y000	Paranoia querulans
E12yz00	Other paranoid states NOS
E12z.00	Paranoid psychosis NOS
E13..00	Other nonorganic psychoses
E13..11	Reactive psychoses
E130.00	Reactive depressive psychosis
E130.11	Psychotic reactive depression
E131.00	Acute hysterical psychosis
E132.00	Reactive confusion
E133.00	Acute paranoid reaction
E133.11	Bouffee delirante
E134.00	Psychogenic paranoid psychosis

E13y.00	Other reactive psychoses
E13y000	Psychogenic stupor
E13y100	Brief reactive psychosis
E13yz00	Other reactive psychoses NOS
E13z.00	Nonorganic psychosis NOS
E13z.11	Psychotic episode NOS
E212200	Schizotypal personality
Eu2..00	[X]Schizophrenia, schizotypal and delusional disorders
Eu20.00	[X]Schizophrenia
Eu20000	[X]Paranoid schizophrenia
Eu20011	[X]Paraphrenic schizophrenia
Eu20100	[X]Hebephrenic schizophrenia
Eu20111	[X]Disorganised schizophrenia
Eu20200	[X]Catatonic schizophrenia
Eu20211	[X]Catatonic stupor
Eu20212	[X]Schizophrenic catalepsy
Eu20213	[X]Schizophrenic catatonia
Eu20214	[X]Schizophrenic flexibilatis cerea
Eu20300	[X]Undifferentiated schizophrenia
Eu20311	[X]Atypical schizophrenia
Eu20400	[X]Post-schizophrenic depression
Eu20500	[X]Residual schizophrenia
Eu20511	[X]Chronic undifferentiated schizophrenia
Eu20512	[X]Restzustand schizophrenic
Eu20600	[X]Simple schizophrenia
Eu20y00	[X]Other schizophrenia
Eu20y11	[X]Cenesthopathic schizophrenia
Eu20y12	[X]Schizophreniform disord NOS
Eu20y13	[X]Schizophrenifrm psychos NOS
Eu20z00	[X]Schizophrenia, unspecified
Eu21.00	[X]Schizotypal disorder
Eu21.11	[X]Latent schizophrenic reaction
Eu21.12	[X]Borderline schizophrenia
Eu21.13	[X]Latent schizophrenia
Eu21.14	[X]Prepsychotic schizophrenia
Eu21.15	[X]Prodromal schizophrenia
Eu21.16	[X]Pseudoneurotic schizophrenia
Eu21.17	[X]Pseudopsychopathic schizophrenia
Eu21.18	[X]Schizotypal personality disorder
Eu22.00	[X]Persistent delusional disorders
Eu22000	[X]Delusional disorder
Eu22011	[X]Paranoid psychosis
Eu22012	[X]Paranoid state
Eu22013	[X]Paraphrenia - late
Eu22014	[X]Sensitiver Beziehungswahn
Eu22015	[X]Paranoia
Eu22100	[X]Delusional misidentification syndrome
Eu22111	[X]Capgras syndrome
Eu22200	[X]Cotard syndrome
Eu22300	[X]Paranoid state in remission

Eu22y00	[X]Other persistent delusional disorders
Eu22y11	[X]Delusional dysmorphophobia
Eu22y12	[X]Involutional paranoid state
Eu22y13	[X]Paranoia querulans
Eu22z00	[X]Persistent delusional disorder, unspecified
Eu23.00	[X]Acute and transient psychotic disorders
Eu23000	[X]Acute polymorphic psychot disord without symp of schizoph
Eu23011	[X]Bouffee delirante
Eu23012	[X]Cycloid psychosis
Eu23100	[X]Acute polymorphic psychot disord with symp of schizophren
Eu23111	[X]Bouffee delirante with symptoms of schizophrenia
Eu23112	[X]Cycloid psychosis with symptoms of schizophrenia
Eu23200	[X]Acute schizophrenia-like psychotic disorder
Eu23211	[X]Brief schizophreniform disorder
Eu23212	[X]Brief schizophrenifrm psych
Eu23213	[X]Oneirophrenia
Eu23214	[X]Schizophrenic reaction
Eu23300	[X]Other acute predominantly delusional psychotic disorders
Eu23312	[X]Psychogenic paranoid psychosis
Eu23y00	[X]Other acute and transient psychotic disorders
Eu23z00	[X]Acute and transient psychotic disorder, unspecified
Eu23z11	[X]Brief reactive psychosis NOS
Eu23z12	[X]Reactive psychosis
Eu24.00	[X]Induced delusional disorder
Eu24.11	[X]Folie a deux
Eu24.12	[X]Induced paranoid disorder
Eu24.13	[X]Induced psychotic disorder
Eu25.00	[X]Schizoaffective disorders
Eu25000	[X]Schizoaffective disorder, manic type
Eu25011	[X]Schizoaffective psychosis, manic type
Eu25012	[X]Schizophreniform psychosis, manic type
Eu25100	[X]Schizoaffective disorder, depressive type
Eu25111	[X]Schizoaffective psychosis, depressive type
Eu25112	[X]Schizophreniform psychosis, depressive type
Eu25200	[X]Schizoaffective disorder, mixed type
Eu25211	[X]Cyclic schizophrenia
Eu25212	[X]Mixed schizophrenic and affective psychosis
Eu25y00	[X]Other schizoaffective disorders
Eu25z00	[X]Schizoaffective disorder, unspecified
Eu25z11	[X]Schizoaffective psychosis NOS
Eu26.00	[X]Nonorganic psychosis in remission
Eu2y.00	[X]Other nonorganic psychotic disorders
Eu2y.11	[X]Chronic hallucinatory psychosis
Eu2z.00	[X]Unspecified nonorganic psychosis
Eu2z.11	[X]Psychosis NOS
Eu30.00	[X]Manic episode
Eu30.11	[X]Bipolar disorder, single manic episode
Eu30000	[X]Hypomania
Eu30100	[X]Mania without psychotic symptoms
Eu30200	[X]Mania with psychotic symptoms

Eu30211	[X]Mania with mood-congruent psychotic symptoms
Eu30212	[X]Mania with mood-incongruent psychotic symptoms
Eu30213	[X]Manic stupor
Eu30y00	[X]Other manic episodes
Eu30z00	[X]Manic episode, unspecified
Eu30z11	[X]Mania NOS
Eu31.00	[X]Bipolar affective disorder
Eu31.11	[X]Manic-depressive illness
Eu31.12	[X]Manic-depressive psychosis
Eu31.13	[X]Manic-depressive reaction
Eu31000	[X]Bipolar affective disorder, current episode hypomanic
Eu31100	[X]Bipolar affect disorder cur epi manic wout psychotic symp
Eu31200	[X]Bipolar affect disorder cur epi manic with psychotic symp
Eu31300	[X]Bipolar affect disorder cur epi mild or moderate depressn
Eu31400	[X]Bipol aff disord, curr epis sev depress, no psychot symp
Eu31500	[X]Bipolar affect dis cur epi severe depres with psyc symp
Eu31600	[X]Bipolar affective disorder, current episode mixed
Eu31700	[X]Bipolar affective disorder, currently in remission
Eu31800	[X]Bipolar affective disorder type I
Eu31900	[X]Bipolar affective disorder type II
Eu31911	[X]Bipolar II disorder
Eu31y00	[X]Other bipolar affective disorders
Eu31y11	[X]Bipolar II disorder
Eu31y12	[X]Recurrent manic episodes
Eu31z00	[X]Bipolar affective disorder, unspecified
Eu32300	[X]Severe depressive episode with psychotic symptoms
Eu32311	[X]Single episode of major depression and psychotic symptoms
Eu32312	[X]Single episode of psychogenic depressive psychosis
Eu32313	[X]Single episode of psychotic depression
Eu32314	[X]Single episode of reactive depressive psychosis
Eu32800	[X]Major depression, severe with psychotic symptoms
Eu33300	[X]Recurrent depress disorder cur epi severe with psyc symp
Eu33311	[X]Endogenous depression with psychotic symptoms
Eu33312	[X]Manic-depress psychosis,depressed type+psychotic symptoms
Eu33313	[X]Recurr severe episodes/major depression+psychotic symptom
Eu33314	[X]Recurr severe episodes/psychogenic depressive psychosis
Eu33315	[X]Recurrent severe episodes of psychotic depression
Eu33316	[X]Recurrent severe episodes/reactive depressive psychosis
Eu32A00	[X]Recurr major depr ep, severe with psych, psych in remiss
Eu32900	[X]Single major depr ep, severe with psych, psych in remiss

HIV

medcode	medname
43C3.00	HTLV-3 antibody positive
43C3.11	HIV positive
65QA.00	AIDS carrier
65VE.00	Notification of AIDS
66j..00	Human immunodeficiency virus monitoring
66j0.00	Human immunodeficiency virus annual review
9kl..00	HIV pos gen health check serv declind - enhanc service admin
9kl..11	HIV positive general health check service declined
A788.00	Acquired immune deficiency syndrome
A788.11	Human immunodeficiency virus infection
A788000	Acute human immunodeficiency virus infection
A788100	Asymptomatic human immunodeficiency virus infection
A788200	HIV infection with persistent generalised lymphadenopathy
A788300	Human immunodeficiency virus with constitutional disease
A788400	Human immunodeficiency virus with neurological disease
A788500	Human immunodeficiency virus with secondary infection
A788600	Human immunodeficiency virus with secondary cancers
A788U00	HIV disease result/haematological+immunologic abnorms,NEC
A788V00	HIV disease resulting in multiple diseases CE
A788W00	HIV disease resulting in unspecified malignant neoplasm
A788X00	HIV disease resulting/unspcf infectious+parasitic disease
A788y00	Human immunodeficiency virus with other clinical findings
A788z00	Acquired human immunodeficiency virus infection syndrome NOS
A789.00	Human immunodef virus resulting in other disease
A789000	HIV disease resulting in mycobacterial infection
A789100	HIV disease resulting in cytomegaloviral disease
A789200	HIV disease resulting in candidiasis
A789300	HIV disease resulting in Pneumocystis carinii pneumonia
A789400	HIV disease resulting in multiple infections
A789500	HIV disease resulting in Kaposi's sarcoma
A789600	HIV disease resulting in Burkitt's lymphoma
A789700	HIV dis resulting oth types of non-Hodgkin's lymphoma
A789800	HIV disease resulting in multiple malignant neoplasms
A789900	HIV disease resulting in lymphoid interstitial pneumonitis
A789A00	HIV disease resulting in wasting syndrome
A789X00	HIV dis reslt/oth mal neopl/lymph,h'matopoetc+reltd tissu
A798.00	Retrovirus infection
A7y0100	Retrovirus as cause of diseases classified to other chapters
AyuC.00	[X]Human immunodeficiency virus disease
AyuC000	[X]HIV disease resulting in other bacterial infections
AyuC100	[X]HIV disease resulting in other viral infections
AyuC200	[X]HIV disease resulting in other mycoses
AyuC300	[X]HIV disease resulting in multiple infections
AyuC400	[X]HIV disease resulting/other infectious+parasitic diseases
AyuC500	[X]HIV disease resulting/unspcf infectious+parasitic disease
AyuC600	[X]HIV disease resulting in other non-Hodgkin's lymphoma
AyuC700	[X]HIV dis reslt/oth mal neopl/lymph,h'matopoetc+reltd tissu
AyuC800	[X]HIV disease resulting in other malignant neoplasms

AyuC900	[X]HIV disease resulting in unspecified malignant neoplasm
AyuCA00	[X]HIV disease resulting in multiple diseases CE
AyuCB00	[X]HIV disease result/haematological+immunologic abnorms,NEC
AyuCC00	[X]HIV disease resulting in other specified conditions
AyuCD00	[X]Unspecified human immunodeficiency virus [HIV] disease
AyuD800	[X]Retrovirus infections, not elsewhere classified
AyuKM00	[X]Retrovirus/cause of diseases classified to other chapters
Eu02400	[X]Dementia in human immunodef virus [HIV] disease
R109.00	[D]Laboratory evidence of human immunodefiency virus [HIV]

Rheumatoid arthiritis

medcode	description
G5y8.00	Rheumatoid myocarditis
G5yA.00	Rheumatoid carditis
N040.00	Rheumatoid arthritis
N040000	Rheumatoid arthritis of cervical spine
N040100	Other rheumatoid arthritis of spine
N040200	Rheumatoid arthritis of shoulder
N040300	Rheumatoid arthritis of sternoclavicular joint
N040400	Rheumatoid arthritis of acromioclavicular joint
N040500	Rheumatoid arthritis of elbow
N040600	Rheumatoid arthritis of distal radio-ulnar joint
N040700	Rheumatoid arthritis of wrist
N040800	Rheumatoid arthritis of MCP joint
N040900	Rheumatoid arthritis of PIP joint of finger
N040A00	Rheumatoid arthritis of DIP joint of finger
N040B00	Rheumatoid arthritis of hip
N040C00	Rheumatoid arthritis of sacro-iliac joint
N040D00	Rheumatoid arthritis of knee
N040E00	Rheumatoid arthritis of tibio-fibular joint
N040F00	Rheumatoid arthritis of ankle
N040G00	Rheumatoid arthritis of subtalar joint
N040H00	Rheumatoid arthritis of talonavicular joint
N040J00	Rheumatoid arthritis of other tarsal joint
N040K00	Rheumatoid arthritis of 1st MTP joint
N040L00	Rheumatoid arthritis of lesser MTP joint
N040M00	Rheumatoid arthritis of IP joint of toe
N040N00	Rheumatoid vasculitis
N040P00	Seronegative rheumatoid arthritis
N040Q00	Rheumatoid bursitis
N040R00	Rheumatoid nodule
N040S00	Rheumatoid arthritis - multiple joint
N040T00	Flare of rheumatoid arthritis
N041.00	Felty's syndrome
N042.00	Other rheumatoid arthropathy + visceral/systemic involvement
N042100	Rheumatoid lung disease
N042200	Rheumatoid nodule
N042z00	Rheumatoid arthropathy + visceral/systemic involvement NOS
N047.00	Seropositive errosive rheumatoid arthritis
N04X.00	Seropositive rheumatoid arthritis, unspecified
N04y000	Rheumatoid lung
N04z.	Inflammatory polyarthropathy NOS
Nyu1000	[X]Rheumatoid arthritis+involvement/other organs or systems
Nyu1100	[X]Other seropositive rheumatoid arthritis
Nyu1200	[X]Other specified rheumatoid arthritis
Nyu1G00	[X]Seropositive rheumatoid arthritis, unspecified

Inflammatory conditions

medcode	description
AD61.	Behcet's syndrome
K4252	Ulceration of vulva in Behcet's disease
N000.00	Systemic lupus erythematosus
N0000	Disseminated lupus erythematosus
N0001	Libman-Sacks disease
N0002	Drug-induced systemic lupus erythematosus
N0003	Systemic lupus erythematosus with organ or system involvement
N0004	Systemic lupus erythematosus with pericarditis
N0006	Cerebral lupus
N000z	Systemic lupus erythematosus NOS
N001.	Scleroderma
N0010	Progressive systemic sclerosis
N0011	CREST syndrome
N0012	Systemic sclerosis induced by drugs and chemicals
N002.	Sicca (Sjogren's) syndrome
N005.	Adult Still's Disease
N006.	Antiphospholipid syndrome
N0120	Behcet's syndrome arthropathy
N04y200	Adult-onset Still's disease

Ethnicity

medcode	description	ethnic_group
9S1..00	White	White
9S10.00	White British	White
9S11.00	White Irish	White
9S12.00	Other white ethnic group	White
9S13.00	White Scottish	White
9S14.00	Other white British ethnic group	White
9S2..00	Black Caribbean	Black
9S3..00	Black African	Black
9S4..00	Black, other, non-mixed origin	Black
9S41.00	Black British	Black
9S42.00	Black Caribbean/W.I./Guyana	Black
9S42.11	Black Caribbean	Black
9S42.12	Black West Indian	Black
9S42.13	Black Guyana	Black
9S43.00	Black N African/Arab/Iranian	Black
9S43.11	Black North African	Black
9S43.12	Black Arab	Black
9S43.13	Black Iranian	Black
9S44.00	Black - other African country	Black
9S45.00	Black E Afric Asia/Indo-Caribb	Black
9S45.11	Black East African Asian	Black
9S45.12	Black Indo-Caribbean	Black
9S46.00	Black Indian sub-continent	Black
9S47.00	Black - other Asian	Black
9S48.00	Black Black - other	Black
9S5..00	Black - other, mixed	Mixed
9S51.00	Other Black - Black/White orig	Mixed
9S52.00	Other Black - Black/Asian orig	Mixed
9S6..00	Indian	Asian
9S7..00	Pakistani	Asian
9S8..00	Bangladeshi	Asian
9S9..00	Chinese	Chinese
9SA..00	Other ethnic non-mixed (NMO)	Other
9SA1.00	Brit. ethnic minor. spec.(NMO)	Other
9SA2.00	Brit. ethnic minor. unsp (NMO)	Other
9SA3.00	Caribbean I./W.I./Guyana (NMO)	Black
9SA3.11	Caribbean Island (NMO)	Black
9SA3.12	West Indian (NMO)	Black
9SA3.13	Guyana (NMO)	Black
9SA4.00	N African Arab/Iranian (NMO)	Other
9SA4.11	North African Arab (NMO)	Other
9SA4.12	Iranian (NMO)	Other
9SA5.00	Other African countries (NMO)	Black
9SA6.00	E Afric Asian/Indo-Carib (NMO)	Asian
9SA6.11	East African Asian (NMO)	Asian
9SA6.12	Indo-Caribbean (NMO)	Asian
9SA7.00	Indian sub-continent (NMO)	Asian
9SA8.00	Other Asian (NMO)	Asian

9SA9.00	Irish (NMO)	White
9SAA.00	Greek/Greek Cypriot (NMO)	White
9SAA.11	Greek (NMO)	White
9SAA.12	Greek Cypriot (NMO)	White
9SAB.00	Turkish/Turkish Cypriot (NMO)	White
9SAB.11	Turkish (NMO)	White
9SAB.12	Turkish Cypriot (NMO)	White
9SAC.00	Other European (NMO)	White
9SAD.00	Other ethnic NEC (NMO)	White
9SB..00	Other ethnic, mixed origin	Mixed
9SB1.00	Other ethnic, Black/White orig	Mixed
9SB2.00	Other ethnic, Asian/White orig	Mixed
9SB3.00	Other ethnic, mixed white orig	Mixed
9SB4.00	Other ethnic, other mixed orig	Mixed
9SB5.00	Black Caribbean and White	Mixed
9SB6.00	Black African and White	Mixed
9SC..00	Vietnamese	Other
9SG..00	Other black ethnic group	Black
9SH..00	Other Asian ethnic group	Asian
9SI..00	Irish traveller	White
9SJ..00	Other ethnic group	Other
9T1..00	New Zealand ethnic groups	Other
9T11.00	New Zealand European	Other
9T11.11	Pakeha	Other
9T12.00	Other European in New Zealand	Other
9T13.00	New Zealand Maori	Other
9T14.00	Samoan	Other
9T15.00	Cook Island Maori	Other
9T16.00	Tongan	Other
9T17.00	Niuean	Other
9T18.00	Tokelauan	Other
9T19.00	Fijian	Other
9T1A.00	Other Pacific ethnic group	Other
9T1B.00	South East Asian	Asian
9T1C.00	Chinese	Chinese
9T1D.00	Indian	Asian
9T1E.00	Other Asian	Asian
9T1Y.00	Other New Zealand ethnic group	Other
9T1Z.00	New Zealand ethnic group NOS	Other
9T2..00	Traveller - gypsy	White
9T3..00	Yemeni	Other
9T4..00	Romanian	Other
9T5..00	Bulgarian	Other
9T6..00	Czech	Other
9T7..00	Slovak	Other
9T8..00	Portuguese	Other
9T9..00	Nepali	Other
9i0..00	British or mixed British - ethnic category 2001	White
9i00.00	White British - ethnic category 2001 census	White
9i1..00	Irish - ethnic category 2001 census	White

9i10.00	White Irish - ethnic category 2001 census	White
9i2..00	Other White background - ethnic category 20	White
9i20.00	English - ethnic category 2001 census	White
9i21.00	Scottish - ethnic category 2001 census	White
9i22.00	Welsh - ethnic category 2001 census	White
9i23.00	Cornish - ethnic category 2001 census	White
9i24.00	Northern Irish - ethnic category 2001 census	White
9i25.00	Ulster Scots - ethnic category 2001 census	White
9i26.00	Cypriot (part not stated) - ethnic category 20(	White
9i27.00	Greek - ethnic category 2001 census	White
9i28.00	Greek Cypriot - ethnic category 2001 census	White
9i29.00	Turkish - ethnic category 2001 census	White
9i2A.00	Turkish Cypriot - ethnic category 2001 census	White
9i2B.00	Italian - ethnic category 2001 census	White
9i2C.00	Irish Traveller - ethnic category 2001 census	White
9i2D.00	Traveller - ethnic category 2001 census	White
9i2E.00	Gypsy/Romany - ethnic category 2001 census	White
9i2F.00	Polish - ethnic category 2001 census	White
9i2G.00	Baltic Estonian/Latvian/Lithuanian - ethn cate	White
9i2H.00	Commonwealth (Russian) Indep States - ethn	White
9i2J.00	Kosovan - ethnic category 2001 census	White
9i2K.00	Albanian - ethnic category 2001 census	White
9i2L.00	Bosnian - ethnic category 2001 census	White
9i2M.00	Croatian - ethnic category 2001 census	White
9i2N.00	Serbian - ethnic category 2001 census	White
9i2P.00	Other republics former Yugoslavia - ethnic cat	White
9i2Q.00	Mixed Irish and other White - ethnic category	White
9i2R.00	Oth White European/European unsp/Mixed E	White
9i2S.00	Other mixed White - ethnic category 2001 cer	White
9i2T.00	Other White or White unspecified ethnic cate	White
9i3..00	White and Black Caribbean - ethnic category 2	Mixed
9i4..00	White and Black African - ethnic category 200	Mixed
9i5..00	White and Asian - ethnic category 2001 censu	Mixed
9i6..00	Other Mixed background - ethnic category 20	Mixed
9i60.00	Black and Asian - ethnic category 2001 census	Mixed
9i61.00	Black and Chinese - ethnic category 2001 cens	Mixed
9i62.00	Black and White - ethnic category 2001 censu	Mixed
9i63.00	Chinese and White - ethnic category 2001 cer	Mixed
9i64.00	Asian and Chinese - ethnic category 2001 cen	Mixed
9i65.00	Other Mixed or Mixed unspecified ethnic cate	Mixed
9i7..00	Indian or British Indian - ethnic category 2001	Asian
9i8..00	Pakistani or British Pakistani - ethnic category	Asian
9i9..00	Bangladeshi or British Bangladeshi - ethn cate	Asian
9iA..00	Other Asian background - ethnic category 20(	Asian
9iA1.00	Punjabi - ethnic category 2001 census	Asian
9iA2.00	Kashmiri - ethnic category 2001 census	Asian
9iA3.00	East African Asian - ethnic category 2001 cens	Asian
9iA4.00	Sri Lankan - ethnic category 2001 census	Asian
9iA5.00	Tamil - ethnic category 2001 census	Asian
9iA6.00	Sinhalese - ethnic category 2001 census	Asian

9iA7.00	Caribbean Asian - ethnic category 2001 censu	Asian
9iA8.00	British Asian - ethnic category 2001 census	Asian
9iA9.00	Mixed Asian - ethnic category 2001 census	Asian
9iAA.00	Other Asian or Asian unspecified ethnic categ	Asian
9iB..00	Caribbean - ethnic category 2001 census	Black
9iC..00	African - ethnic category 2001 census	Black
9iD..00	Other Black background - ethnic category 200	Black
9iD0.00	Somali - ethnic category 2001 census	Black
9iD1.00	Nigerian - ethnic category 2001 census	Black
9iD2.00	Black British - ethnic category 2001 census	Black
9iD3.00	Mixed Black - ethnic category 2001 census	Black
9iD4.00	Other Black or Black unspecified ethnic catego	Black
9iE..00	Chinese - ethnic category 2001 census	Chinese
9iF..00	Other - ethnic category 2001 census	Other
9iF0.00	Vietnamese - ethnic category 2001 census	Other
9iF1.00	Japanese - ethnic category 2001 census	Other
9iF2.00	Filipino - ethnic category 2001 census	Other
9iF3.00	Malaysian - ethnic category 2001 census	Other
9iF9.00	Arab - ethnic category 2001 census	Other
9iFA.00	North African - ethnic category 2001 census	Other
9iFB.00	Mid East (excl Israeli, Iranian & Arab) - eth cat	Other
9iFC.00	Israeli - ethnic category 2001 census	Other
9iFD.00	Iranian - ethnic category 2001 census	Other
9iFE.00	Kurdish - ethnic category 2001 census	Other
9iFF.00	Moroccan - ethnic category 2001 census	Other
9iFG.00	Latin American - ethnic category 2001 census	Other
9iFH.00	South and Central American - ethnic category	Other
9iFJ.00	Mauritian/Seychellois/Maldivian/St Helena et	Other
9iFK.00	Any other group - ethnic category 2001 censu	Other

Smoking

medcode	description	group
137..00	tobacco consumption	current smoker
137..11	smoker - amount smoked	current smoker
1371	never smoked tobacco	non-smoker
1371.11	non-smoker	non-smoker
1372	trivial smoker - < 1 cig/day	current smoker
1372.11	occasional smoker	current smoker
1373	light smoker - 1-9 cigs/day	current smoker
1374	moderate smoker - 10-19 cigs/d	current smoker
1375	heavy smoker - 20-39 cigs/day	current smoker
1376	very heavy smoker - 40+cigs/d	current smoker
1377	ex-trivial smoker (<1/day)	ex-smoker
1378	ex-light smoker (1-9/day)	ex-smoker
1379	ex-moderate smoker (10-19/day)	ex-smoker
137A.00	ex-heavy smoker (20-39/day)	ex-smoker
137B.00	ex-very heavy smoker (40+/day)	ex-smoker
137C.00	keeps trying to stop smoking	current smoker
137F.00	ex-smoker - amount unknown	ex-smoker
137G.00	trying to give up smoking	current smoker
137H.00	pipe smoker	current smoker
137J.00	cigar smoker	current smoker
137K.00	stopped smoking	ex-smoker
137K000	recently stopped smoking	ex-smoker
137L.00	current non-smoker	ex-smoker
137M.00	rolls own cigarettes	current smoker
137N.00	ex pipe smoker	ex-smoker
137O.00	ex cigar smoker	ex-smoker
137P.00	cigarette smoker	current smoker
137P.11	smoker	current smoker
137Q.00	smoking started	current smoker
137Q.11	smoking restarted	current smoker
137R.00	current smoker	current smoker
137S.00	ex smoker	ex-smoker
137T.00	date ceased smoking	ex-smoker
137V.00	smoking reduced	current smoker
137W.00	chews tobacco	current smoker
137X.00	cigarette consumption	current smoker
137Y.00	cigar consumption	current smoker
137Z.00	tobacco consumption nos	current smoker
137a.00	pipe tobacco consumption	current smoker
137b.00	ready to stop smoking	current smoker
137c.00	thinking about stopping smoking	current smoker
137d.00	not interested in stopping smoking	current smoker
137e.00	smoking restarted	current smoker
137f.00	reason for restarting smoking	current smoker
137g.00	cigarette pack-years	current smoker
137h.00	minutes from waking to first tobacco c	current smoker
137i.00	ex-tobacco chewer	ex-smoker
137j.00	ex-cigarette smoker	ex-smoker

137l.00	ex roll-up cigarette smoker	ex-smoker
137m.00	failed attempt to stop smoking	current smoker
137n.00	total time smoked	current smoker
137o.00	waterpipe tobacco consumption	current smoker
13p..00	smoking cessation milestones	ex-smoker
13p4.00	smoking free weeks	ex-smoker
13p5.00	smoking cessation programme start da	ex-smoker
13p5000	practice based smoking cessation prog	ex-smoker
13p8.00	lost to smoking cessation follow-up	ex-smoker
6791	health ed. - smoking	current smoker
67A3.00	pregnancy smoking advice	current smoker
67H1.00	lifestyle advice regarding smoking	current smoker
67H6.00	brief intervention for smoking cessatic	ex-smoker
745H.00	smoking cessation therapy	ex-smoker
745H400	smoking cessation drug therapy	ex-smoker
745Hy00	other specified smoking cessation ther	ex-smoker
745Hz00	smoking cessation therapy nos	ex-smoker
8CAL.00	smoking cessation advice	current smoker
8CAg.00	smoking cessation advice provided by	current smoker
8HBM.00	stop smoking face to face follow-up	ex-smoker
8HBP.00	smoking cessation 12 week follow-up	ex-smoker
8IAj.00	smoking cessation advice declined	current smoker
8IEK.00	smoking cessation programme decline	current smoker
8IEM.00	smoking cessation drug therapy declin	current smoker
8IEo.00	referral to smoking cessation service d	current smoker
9OO2.00	refuses stop smoking monitor	current smoker
9km..00	ex-smoker annual review - enhanced s	ex-smoker
9km..11	ex-smoker annual review	ex-smoker
9kn..00	non-smoker annual review - enhanced	non-smoker
9kn..11	non-smoker annual review	non-smoker
9ko..00	current smoker annual review - enhan	current smoker
9ko..11	current smoker annual review	current smoker
E251.00	tobacco dependence	current smoker
E251000	tobacco dependence, unspecified	current smoker
E251100	tobacco dependence, continuous	current smoker
E251200	tobacco dependence, episodic	current smoker
E251z00	tobacco dependence nos	current smoker
ZG23300	advice on smoking	current smoker
ZV4K000	[v]tobacco use	current smoker
ZV6D800	[v]tobacco abuse counselling	current smoker

Statin Contraindicated

medcode	description
8I27.00	Statins contraindicated
8I27000	Simvastatin contraindicated
8I2C.00	Lipid lowering therapy contraindicated
8I3C.00	Statin declined
8I3J.00	Lipid lowering therapy declined
8I76.00	Statin not tolerated
TJC2.00	Adverse reaction to antilipaemic/anti-arteriosclerotic drugs
TJC2400	Adverse reaction to simvastatin
TJC2500	Adverse reaction to pravastatin
TJC2z00	Adverse reaction to antilipaemic/antiarterioscler drugs NOS
U60C600	[X]Antihyperlipidaem/antiarterioscl drg caus adv ef ther use
U60C611	[X] Adverse react to antilipaemic & anti-arteriosclerot drug
U60C615	[X] Adverse reaction to simvastatin
U60C616	[X] Adverse reaction to pravastatin
U60C617	[X] Adverse react to antilipaemic/antiarterioscler drugs NOS
U60C900	[X]Lipid-lowering drug adverse reaction
U60CA00	[X]Statin causing adverse effect in therapeutic use
