# Supplementary material for: Cohort study investigating the relationship between cholesterol, cardiovascular risk score and the prescribing of statins in UK primary care: study protocol
Source: BMJ Open. 2016 Nov 17;6(11):e013120. doi: 10.1136/bmjopen-2016-013120 (PMC5128938; doi:10.1136/bmjopen-2016-013120)
Supplement: supplementary appendix II [file bmjopen-2016-013120supp_appendixII.pdf]

Lipid lowering therapy

drugcode	genericname
57832979	Atorvastatin 20mg tablets
57833979	Atorvastatin 10mg tablets
57838979	Atorvastatin 40mg tablets
58654979	Atorvastatin 20mg tablets
58661979	Atorvastatin 10mg tablets
58669979	Atorvastatin 40mg tablets
58671979	Atorvastatin 40mg tablets
61212979	ATORVASTATIN 20mg chew tabs
61213979	Atorvastatin 20mg chewable tablets sugar free
61215979	Atorvastatin 10mg chewable tablets sugar free
61489979	Simvastatin 20mg tablets
62570979	Simvastatin 40mg/5ml oral suspension sugar free
62597979	Simvastatin 20mg/5ml oral suspension sugar free
64839979	Simvastatin 40mg/5ml oral suspension
79254979	Simvastatin 20mg/5ml oral suspension
81048998	ATORVASTATIN 20mg chew tabs
81049998	Atorvastatin 20mg chewable tablets sugar free
81050998	ATORVASTATIN 10mg chew tabs
81051998	Atorvastatin 10mg chewable tablets sugar free
82655998	NICOTINC/LAROP 1g/20mg m/r tab
83030998	SIMVASTATIN 80mg tablets
83099998	Simvastatin 40mg/5ml oral solution sugar free
83187998	BEZAFIBRATE 400mg m/r tablets
83188998	BEZAFIBRATE 200mg tablets
83594998	Nicotinic acid 1g / Laropiprant 20mg modified-release tablets
84267998	COLESEVELAM 625mg tablets
84268998	Colesevelam 625mg tablets
86020998	Simvastatin 20mg/5ml oral solution sugar free
86467998	ROSUVASTATIN 5mg tablets
86468998	Rosuvastatin 5mg tablets
86787998	Simvastatin 80mg with ezetimibe 10mg tablet
86788998	Simvastatin 40mg with ezetimibe 10mg tablet
86789998	Simvastatin 20mg with ezetimibe 10mg tablet
86791998	SIMVAST 80mg/EZETIMIB 10mg tab
86794998	Simvastatin 80mg / Ezetimibe 10mg tablets
86795998	SIMVAST 40mg/EZETIMIB 10mg tab
86796998	Simvastatin 40mg / Ezetimibe 10mg tablets
86797998	SIMVAST 20mg/EZETIMIB 10mg tab
86798998	Simvastatin 20mg / Ezetimibe 10mg tablets
87025998	BEZAFIBRATE 400mg m/r tablets
87373998	SIMVASTATIN 10mg tablets
87418998	SIMVASTATIN 10mg tablets
87760998	COLESTIPOL orang 5g/sach grans
87848998	NICOTINIC ACID starter pack
87849998	Nicotinic acid 375mg + 500mg + 750mg Modified-release tablet
87850998	NICOTINIC ACID 1000mg m/r tab
87851998	NICOTINIC ACID 750mg m/r tabs
87852998	NICOTINIC ACID 500mg m/r tabs

87853998 Nicotinic acid 1g modified-release tablets
87854998 Nicotinic acid 750mg modified-release tablets
87855998 Nicotinic acid 500mg modified-release tablets
87916998 SIMVASTATIN 40mg tablets
87917998 SIMVASTATIN 20mg tablets
87918998 SIMVASTATIN 10mg tablets
88297996 FENOFIBRATE 267mg capsules
88297997 FENOFIBRATE 200mg capsules
88297998 FENOFIBRATE 67mg capsules
88298996 Fenofibrate micronised 200mg capsules
88298997 Fenofibrate micronised 267mg capsules
88298998 Fenofibrate micronised 67mg capsules
88534998 Rosuvastatin 10mg tablets
89089998 BEZAFIBRATE 400mg m/r tablets
89153996 CERIVASTATIN Na 300mcg tabs
89153997 CERIVASTATIN Na 200mcg tabs
89153998 CERIVASTATIN Na 100mcgs tabs
89154996 Cerivastatin 300microgram tablets
89154997 Cerivastatin 200microgram tablets
89154998 Cerivastatin 100microgram tablets
89306996 ATORVASTATIN 40mg tablets
89306997 ATORVASTATIN 20mg tablets
89306998 ATORVASTATIN 10mg tablets
89311996 Atorvastatin 40mg tablets
89311997 Atorvastatin 20mg tablets
89311998 Atorvastatin 10mg tablets
89401998 BEZAFIBRATE 400mg m/r tablets
89617998 ISPAG HSK 3.5g ornge s/f gran
90309998 ATORVASTATIN 80mg tablets
90310998 Atorvastatin 80mg tablets
90649998 FENOFIBRATE 200mg capsules
90653998 Colestyramine with aspartame 4g sugar free powder
90973998 Rosuvastatin 20mg tablets
91194998 FLUVASTATIN 80mg m/r tablets
91316998 COLESTYRAMINE powder 4g
92154990 Simvastatin 20mg/5ml oral suspension sugar free
92220998 Simvastatin 80mg tablets
92292998 Ezetimibe 10mg tablets
92293998 EZETIMIBE 10mg tablets
92408998 ROSUVASTATIN 20mg tablets
92409998 ROSUVASTATIN 10mg tablets
92410998 Rosuvastatin 40mg tablets
92447998 CERIVASTATIN Na 400mcg tabs
92448997 Cerivastatin 800microgram tablets
92448998 Cerivastatin 400microgram tablets
92460998 Fenofibrate micronised 160mg tablets
92471998 SIMVASTATIN 80mg tablets
92539998 ROSUVASTATIN 40mg tablets
92549990 Fenofibrate micronised 200mg capsules
92804996 Fluvastatin 80mg modified-release tablets

92804997 Fluvastatin 40mg capsules
92804998 Fluvastatin 20mg capsules
92805997 FLUVASTATIN 40mg capsules
92805998 FLUVASTATIN 20mg capsules
93243996 PRAVASTATIN SODIUM 40mg tabs
93243997 PRAVASTATIN 20mg tablets
93243998 PRAVASTATIN SODIUM 10mg tabs
93244996 Pravastatin 40mg tablets
93244997 Pravastatin 20mg tablets
93244998 Pravastatin 10mg tablets
93541998 Colestyramine 4g oral powder sachets sugar free
93542998 Colestyramine sugar free pdr
93619996 Simvastatin 40mg tablets
93619997 Simvastatin 20mg tablets
93619998 Simvastatin 10mg tablets
93620996 SIMVASTATIN 40mg tablets
93620997 SIMVASTATIN 20mg tablets
93620998 SIMVASTATIN 10mg tablets
93838990 Bezafibrate 200mg tablets
93851992 COLESTIPOL HCl GRA
93871990 Simvastatin 40mg tablets
94112992 CHOLESTYRAMINE 325 MG CAP
94188997 Fenofibrate 200mg capsules
94188998 Fenofibrate 100mg Capsule
94189997 FENOFIBRATE 200mg capsules
94189998 FENOFIBRATE 100mg capsules
94407990 Simvastatin 20mg tablets
94605998 Colestipol with aspartame granules
94661997 COLESTIPOL orang 5g/sach grans
94661998 COLESTIPOL granules 5g sachet
94662998 Colestipol 5g granules sachets sugar free
94782990 Pravastatin 20mg tablets
94789990 Pravastatin 10mg tablets
94799998 FENOFIBRATE 160mg m/r tablets
94827992 CHOLESTYRAMINE POW
94830990 Pravastatin 20mg tablets
94831990 Pravastatin 10mg tablets
94849990 Pravastatin 40mg tablets
94850990 Pravastatin 20mg tablets
94851990 Pravastatin 10mg tablets
94882998 ACIPIMOX 250mg capsules
94883998 Acipimox 250mg capsules
94927990 Simvastatin 80mg tablets
95098992 HEXOPAL 200 MG TAB
95185990 Simvastatin 80mg tablets
95277990 Simvastatin 40mg tablets
95278990 Simvastatin 20mg tablets
95279990 Simvastatin 10mg tablets
95401998 Probucol 250mg tablet
95405990 Simvastatin 40mg tablets

95406990 Simvastatin 20mg tablets
95442990 Simvastatin 80mg tablets
95443990 Simvastatin 40mg tablets
95445990 Simvastatin 10mg tablets
95448990 Simvastatin 80mg tablets
95449990 Simvastatin 40mg tablets
95450990 Simvastatin 20mg tablets
95451990 Simvastatin 10mg tablets
95471990 Simvastatin 40mg tablets
95472990 Simvastatin 20mg tablets
95474990 Simvastatin 40mg tablets
95475990 Simvastatin 20mg tablets
95478990 Simvastatin 40mg tablets
95479990 Simvastatin 20mg tablets
95480990 Simvastatin 10mg tablets
95481990 Simvastatin 40mg tablets
95482990 Simvastatin 20mg tablets
95483990 Simvastatin 10mg tablets
95486990 Simvastatin 40mg tablets
95487990 Simvastatin 20mg tablets
95493990 Simvastatin 40mg tablets
95494990 Simvastatin 20mg tablets
95495990 Simvastatin 10mg tablets
95500990 Simvastatin 80mg tablets
95501990 Simvastatin 40mg tablets
95502990 Simvastatin 20mg tablets
95508990 Simvastatin 10mg tablets
95549990 Simvastatin 40mg tablets
95550990 Simvastatin 20mg tablets
95551990 Simvastatin 10mg tablets
95728998 Nicofuranose 250mg Tablet
95805998 BEZAFIBRATE 400mg m/r tablets
95847990 Colestyramine 4g oral powder sachets sugar free
95952997 Bezafibrate 400mg modified-release tablets
95952998 Bezafibrate 200mg tablets
96021990 Fenofibrate micronised 200mg capsules
96134990 Colestyramine 4g oral powder sachets sugar free
96295997 Gemfibrozil 600mg tablets
96295998 Gemfibrozil 300mg capsules
96642998 Clofibrate 500mg capsules
96656990 Bezafibrate 200mg tablets
96678998 Colestyramine 4g oral powder sachets
96685989 Bezafibrate 200mg tablets
96685990 Bezafibrate 400mg modified-release tablets
97078996 FISH OIL(CONC) oral emulsion
97078997 FISH OIL(CONC) oral liquid
97078998 FISH OIL(CONC) 1g capsules
97247997 GEMFIBROZIL 600mg tablets
97247998 GEMFIBROZIL 300mg capsules
97377979 CERIVASTATIN Na 300mcg tabs

97433979 CIPROFIBRATE 100mg tablets
97455979 Pravastatin 10mg tablets
97476979 Simvastatin 40mg tablets
97494979 Simvastatin 20mg tablets
97508979 SIMVASTATIN 10mg tablets
97595979 Bezafibrate 400mg modified-release tablets
97655990 Colestyramine 4g oral powder sachets sugar free
97705979 CERIVASTATIN Na 100mcgs tabs
97723998 Ciprofibrate 100mg tablets
97751998 CIPROFIBRATE 100mg tablets
97800992 NICOTINIC ACID 500 MG TAB
97808989 Gemfibrozil 600mg tablets
97895997 BEZAFIBRATE 400mg m/r tablets
97895998 BEZAFIBRATE 200mg tablets
98066996 Nicotinic acid 100mg Tablet
98066997 Nicotinic acid 50mg tablets
98066998 Nicotinic acid 25mg Tablet
98108998 NICOFURANOSE 250mg tablets
98455998 CLOFIBRATE 500mg capsules
99014998 BEZAFIBRATE 400mg m/r tablets
99197998 COLESTYRAMINE powder 4g/sachet
99456998 PROBUCOL 250mg tablets
99725990 Nicotinic acid 50mg tablets
99957979 ATORVASTATIN 80mg tablets

Statins

drugcode	genericname
57832979	Atorvastatin 20mg tablets
57833979	Atorvastatin 10mg tablets
57838979	Atorvastatin 40mg tablets
58151979	Atorvastatin 60mg tablets
58153979	Atorvastatin 30mg tablets
58650979	Atorvastatin 20mg tablets
58653979	Atorvastatin 20mg tablets
58654979	Atorvastatin 20mg tablets
58658979	Atorvastatin 10mg tablets
58661979	Atorvastatin 10mg tablets
58668979	Atorvastatin 40mg tablets
58669979	Atorvastatin 40mg tablets
58671979	Atorvastatin 40mg tablets
58711979	Atorvastatin 80mg tablets
59480979	Fluvastatin 20mg capsules
61212979	Atorvastatin 20mg chewable tablets sugar free
61213979	Atorvastatin 20mg chewable tablets sugar free
61215979	Atorvastatin 10mg chewable tablets sugar free
61489979	Simvastatin 20mg tablets
62570979	Simvastatin 40mg/5ml oral suspension sugar free
62597979	Simvastatin 20mg/5ml oral suspension sugar free
64582979	Atorvastatin 40mg/5ml oral suspension
64584979	Atorvastatin 40mg/5ml oral solution
64586979	Atorvastatin 10mg/5ml oral suspension
64588979	Atorvastatin 10mg/5ml oral solution
64634979	Atorvastatin 20mg/5ml oral suspension
64636979	Atorvastatin 20mg/5ml oral solution
64839979	Simvastatin 40mg/5ml oral suspension sugar free
79254979	Simvastatin 20mg/5ml oral suspension sugar free
79256979	Simvastatin 10mg/5ml oral suspension
81048998	Atorvastatin 20mg chewable tablets sugar free
81049998	Atorvastatin 20mg chewable tablets sugar free
81050998	Atorvastatin 10mg chewable tablets sugar free
81051998	Atorvastatin 10mg chewable tablets sugar free
83030998	Simvastatin 80mg tablets
83099998	Simvastatin 40mg/5ml oral suspension sugar free
86020998	Simvastatin 20mg/5ml oral solution sugar free
86467998	Rosuvastatin 5mg tablets
86468998	Rosuvastatin 5mg tablets
86787998	Simvastatin 80mg / Ezetimibe 10mg tablets
86788998	Simvastatin 40mg / Ezetimibe 10mg tablets
86789998	Simvastatin 20mg / Ezetimibe 10mg tablets
86791998	Simvastatin 80mg / Ezetimibe 10mg tablets
86794998	Simvastatin 80mg / Ezetimibe 10mg tablets
86795998	Simvastatin 40mg / Ezetimibe 10mg tablets
86796998	Simvastatin 40mg / Ezetimibe 10mg tablets
86797998	Simvastatin 20mg / Ezetimibe 10mg tablets
86798998	Simvastatin 20mg / Ezetimibe 10mg tablets

87373998 Simvastatin 10mg tablets
87418998 Simvastatin 10mg tablets
87916998 Simvastatin 40mg tablets
87917998 Simvastatin 20mg tablets
87918998 Simvastatin 10mg tablets
88534998 Rosuvastatin 10mg tablets
89153996 Cerivastatin sodium 300mcg tablets
89153997 Cerivastatin sodium 200mcg tablets
89153998 Cerivastatin sodium 100mcg tablets
89154996 Cerivastatin 300microgram tablets
89154997 Cerivastatin 200microgram tablets
89154998 Cerivastatin 100microgram tablets
89306996 Atorvastatin 40mg tablets
89306997 Atorvastatin 20mg tablets
89306998 Atorvastatin 10mg tablets
89311996 Atorvastatin 40mg tablets
89311997 Atorvastatin 20mg tablets
89311998 Atorvastatin 10mg tablets
90309998 Atorvastatin 80mg tablets
90310998 Atorvastatin 80mg tablets
90973998 Rosuvastatin 20mg tablets
91194998 Fluvastatin 80mg modified-release tablets
92154990 Simvastatin 20mg/5ml oral suspension sugar free
92220998 Simvastatin 80mg tablets
92408998 Rosuvastatin 20mg tablets
92409998 Rosuvastatin 10mg tablets
92410998 Rosuvastatin 40mg tablets
92447998 Cerivastatin sodium 400mcg tablets
92448997 Cerivastatin 800microgram tablets
92448998 Cerivastatin 400microgram tablets
92471998 Simvastatin 80mg tablets
92539998 Rosuvastatin 40mg tablets
92804996 Fluvastatin 80mg modified-release tablets
92804997 Fluvastatin 40mg capsules
92804998 Fluvastatin 20mg capsules
92805997 Fluvastatin 40mg capsules
92805998 Fluvastatin 20mg capsules
93243996 Pravastatin 40mg tablets
93243997 Pravastatin 20mg tablets
93243998 Pravastatin 10mg tablets
93244996 Pravastatin 40mg tablets
93244997 Pravastatin 20mg tablets
93244998 Pravastatin 10mg tablets
93619996 Simvastatin 40mg tablets
93619997 Simvastatin 20mg tablets
93619998 Simvastatin 10mg tablets
93620996 Simvastatin 40mg tablets
93620997 Simvastatin 20mg tablets
93620998 Simvastatin 10mg tablets
93871990 Simvastatin 40mg tablets

94407990 Simvastatin 20mg tablets
94782990 Pravastatin 20mg tablets
94789990 Pravastatin 10mg tablets
94830990 Pravastatin 20mg tablets
94831990 Pravastatin 10mg tablets
94849990 Pravastatin 40mg tablets
94850990 Pravastatin 20mg tablets
94851990 Pravastatin 10mg tablets
94920990 Simvastatin 20mg tablets
94927990 Simvastatin 80mg tablets
95185990 Simvastatin 80mg tablets
95277990 Simvastatin 40mg tablets
95278990 Simvastatin 20mg tablets
95279990 Simvastatin 10mg tablets
95372990 Simvastatin 40mg tablets
95405990 Simvastatin 40mg tablets
95406990 Simvastatin 20mg tablets
95442990 Simvastatin 80mg tablets
95443990 Simvastatin 40mg tablets
95445990 Simvastatin 10mg tablets
95448990 Simvastatin 80mg tablets
95449990 Simvastatin 40mg tablets
95450990 Simvastatin 20mg tablets
95451990 Simvastatin 10mg tablets
95471990 Simvastatin 40mg tablets
95472990 Simvastatin 20mg tablets
95474990 Simvastatin 40mg tablets
95475990 Simvastatin 20mg tablets
95478990 Simvastatin 40mg tablets
95479990 Simvastatin 20mg tablets
95480990 Simvastatin 10mg tablets
95481990 Simvastatin 40mg tablets
95482990 Simvastatin 20mg tablets
95483990 Simvastatin 10mg tablets
95486990 Simvastatin 40mg tablets
95487990 Simvastatin 20mg tablets
95493990 Simvastatin 40mg tablets
95494990 Simvastatin 20mg tablets
95495990 Simvastatin 10mg tablets
95500990 Simvastatin 80mg tablets
95501990 Simvastatin 40mg tablets
95502990 Simvastatin 20mg tablets
95508990 Simvastatin 10mg tablets
95549990 Simvastatin 40mg tablets
95550990 Simvastatin 20mg tablets
95551990 Simvastatin 10mg tablets
97377979 Cerivastatin sodium 300mcg tablets
97403979 Fluvastatin 80mg modified-release tablets
97424979 Fluvastatin 20mg capsules
97430979 Fluvastatin 20mg capsules

97454979	Pravastatin 20mg tablets
97455979	Pravastatin 10mg tablets
97476979	Simvastatin 40mg tablets
97482979	Simvastatin 40mg tablets
97494979	Simvastatin 20mg tablets
97495979	Simvastatin 20mg tablets
97508979	Simvastatin 10mg tablets
97518979	Simvastatin 10mg tablets
97612979	Cerivastatin sodium 200mcg tablets
97705979	Cerivastatin sodium 100mcg tablets
97756979	Atorvastatin 40mg tablets
99957979	Atorvastatin 80mg tablets

Antipsychotics

drugcode	genericname
52732979	Lithium citrate 509mg/5ml oral solution
52736979	Quetiapine 200mg tablets
52738979	Quetiapine 25mg tablets
52748979	Risperidone 2mg tablets
53079979	Quetiapine 150mg modified-release tablets
53671979	Levomepromazine 25mg/1ml solution for injection ampoules
54533979	Trifluoperazine 5mg tablets
54534979	Trifluoperazine 1mg tablets
54953979	Paliperidone 150mg/1.5ml suspension for injection pre-filled syringes and paliperidone 100mg/1ml suspension for injectio
55083979	Quetiapine 50mg modified-release tablets
55254978	Quetiapine 150mg modified-release tablets
55266978	Quetiapine 50mg modified-release tablets
55267978	Quetiapine 50mg modified-release tablets
55523979	Risperidone 25mg powder and solvent for suspension for injection vials
55701978	Quetiapine 400mg modified-release tablets
55704978	Quetiapine 200mg modified-release tablets
55706978	Quetiapine 300mg modified-release tablets
58638979	Quetiapine 25mg tablets
58799979	Quetiapine 50mg modified-release tablets
59369979	Quetiapine 200mg modified-release tablets
59370979	Quetiapine 300mg modified-release tablets
59467979	Quetiapine 25mg tablets
59468979	Quetiapine 25mg tablets
59469979	Quetiapine 25mg tablets
59474979	Trifluoperazine 1mg/5ml oral solution sugar free
61131979	Olanzapine 15mg oral lyophilisates sugar free
61145979	Olanzapine 20mg oral lyophilisates sugar free
61165979	Olanzapine 10mg oral lyophilisates sugar free
61166979	Olanzapine 5mg oral lyophilisates sugar free
61579979	Olanzapine 5mg oral lyophilisates sugar free
61581979	Olanzapine 20mg oral lyophilisates sugar free
61583979	Olanzapine 15mg oral lyophilisates sugar free
61585979	Olanzapine 10mg oral lyophilisates sugar free
61602979	Olanzapine 5mg oral lyophilisates sugar free
61610979	Olanzapine 15mg oral lyophilisates sugar free
64621979	Quetiapine 50mg modified-release tablets
64622979	Quetiapine 50mg modified-release tablets
64779979	Chlorpromazine 100mg/5ml oral suspension
65890979	Promazine 50mg/5ml oral suspension
67935979	Haloperidol 1.5mg/5ml oral suspension
68593978	Quetiapine 400mg modified-release tablets
68751978	Lurasidone 74mg tablets
68752978	Lurasidone 37mg tablets
68753978	Lurasidone 37mg tablets
68755978	Lurasidone 18.5mg tablets
69237979	Droperidol 5mg/5ml oral solution
70478978	Quetiapine 50mg modified-release tablets

72638978 Quetiapine 400mg modified-release tablets
72639978 Quetiapine 200mg modified-release tablets
72640978 Quetiapine 300mg modified-release tablets
78405978 Aripiprazole 400mg powder and solvent for suspension for injection vials
78406978 Aripiprazole 400mg powder and solvent for suspension for injection vials
79816978 Risperidone 2mg tablets
79934979 Haloperidol 2mg/5ml oral solution
80969998 Olanzapine 20mg oral lyophilisates sugar free
80970998 Olanzapine 20mg oral lyophilisates sugar free
80971998 Olanzapine 20mg oral lyophilisates sugar free
80972998 Olanzapine 15mg oral lyophilisates sugar free
80973998 Olanzapine 15mg oral lyophilisates sugar free
80974998 Olanzapine 15mg oral lyophilisates sugar free
80976998 Olanzapine 10mg oral lyophilisates sugar free
80977998 Olanzapine 10mg oral lyophilisates sugar free
80978998 Olanzapine 10mg oral lyophilisates sugar free
80979998 Olanzapine 5mg oral lyophilisates sugar free
80980998 Olanzapine 5mg oral lyophilisates sugar free
80981998 Olanzapine 5mg oral lyophilisates sugar free
81040998 Olanzapine 10mg orodispersible tablets sugar free
81041998 Olanzapine 5mg orodispersible tablets sugar free
81081998 Haloperidol 1mg/5ml oral solution
81170998 Asenapine 10mg sublingual tablets sugar free
81172998 Asenapine 5mg sublingual tablets sugar free
81418998 Paliperidone 150mg/1.5ml suspension for injection pre-filled syringes
81419998 Paliperidone 100mg/1ml suspension for injection pre-filled syringes
81420998 Paliperidone 75mg/0.75ml suspension for injection pre-filled syringes
81421998 Paliperidone 50mg/0.5ml suspension for injection pre-filled syringes
81422998 Paliperidone 150mg/1.5ml suspension for injection pre-filled syringes
81423998 Paliperidone 100mg/1ml suspension for injection pre-filled syringes
81424998 Paliperidone 75mg/0.75ml suspension for injection pre-filled syringes
81425998 Paliperidone 50mg/0.5ml suspension for injection pre-filled syringes
81467998 Haloperidol 10mg/5ml oral solution sugar free
81468998 Haloperidol 5mg/5ml oral solution sugar free
81923998 Quetiapine 150mg modified-release tablets
81924998 Quetiapine 150mg modified-release tablets

82198998 Olanzapine embonate 405mg powder and solvent for suspension for injection vials

82199998 Olanzapine embonate 300mg powder and solvent for suspension for injection vials

82201998 Olanzapine embonate 300mg powder and solvent for suspension for injection vials

82202998 Olanzapine embonate 210mg powder and solvent for suspension for injection vials
82225998 Benperidol 250microgram tablets
82798998 Clozapine 50mg/ml oral suspension sugar free
82799998 Clozapine 50mg/ml oral suspension sugar free
82800998 Clozapine 200mg tablets
82801998 Clozapine 200mg tablets
82802998 Clozapine 50mg tablets

82803998 Clozapine 50mg tablets
82892998 Chlorpromazine 100mg tablets
83019998 Pericyazine 10mg tablets
83020998 Pericyazine 2.5mg tablets
83490998 Quetiapine 400mg modified-release tablets
83491998 Quetiapine 300mg modified-release tablets
83492998 Quetiapine 200mg modified-release tablets
83493998 Quetiapine 50mg modified-release tablets
83786998 Haloperidol 5mg/1ml solution for injection ampoules
83787998 Haloperidol 5mg/1ml solution for injection ampoules
83903998 Aripiprazole 9.75mg/1.3ml solution for injection vials
83993998 Quetiapine 400mg modified-release tablets
83994998 Quetiapine 300mg modified-release tablets
83995998 Quetiapine 200mg modified-release tablets
83996998 Quetiapine 50mg modified-release tablets
84523998 Paliperidone 6mg modified-release tablets
84524998 Paliperidone 3mg modified-release tablets
84525998 Paliperidone 9mg modified-release tablets
84526998 Paliperidone 6mg modified-release tablets
84527998 Paliperidone 3mg modified-release tablets
85038998 Risperidone 4mg orodispersible tablets sugar free
85039998 Risperidone 3mg orodispersible tablets sugar free
85040998 Risperidone 4mg orodispersible tablets sugar free
85042998 Risperidone 3mg orodispersible tablets sugar free
85294998 Fluphenazine decanoate 100mg/1ml solution for injection ampoules
85295998 Fluphenazine decanoate 50mg/0.5ml solution for injection ampoules
85296998 Fluphenazine decanoate 100mg/1ml solution for injection ampoules
85297998 Fluphenazine decanoate 50mg/0.5ml solution for injection ampoules
85298998 Fluphenazine decanoate 50mg/2ml solution for injection ampoules
85299998 Fluphenazine decanoate 25mg/1ml solution for injection ampoules
85300998 Fluphenazine decanoate 12.5mg/0.5ml solution for injection ampoules
85301998 Fluphenazine decanoate 50mg/2ml solution for injection ampoules
85302998 Fluphenazine decanoate 25mg/1ml solution for injection ampoules
85303998 Fluphenazine decanoate 12.5mg/0.5ml solution for injection ampoules
85376998 Olanzapine 20mg tablets
85377998 Olanzapine 20mg oral lyophilisates sugar free
85409998 Pipotiazine palmitate 100mg/2ml oily injection
85410998 Pipotiazine 50mg/1ml solution for injection ampoules
85411998 Pipotiazine 100mg/2ml solution for injection ampoules
85413998 Pipotiazine 50mg/1ml solution for injection ampoules
85607998 Zuclopenthixol decanoate 200mg/1ml solution for injection ampoules
85609998 Zuclopenthixol decanoate 200mg/1ml solution for injection ampoules
85613998 Flupentixol 20mg/1ml solution for injection ampoules
85614998 Flupentixol 20mg/1ml solution for injection ampoules
85702998 Chlorpromazine 50mg/2ml solution for injection ampoules
85704998 Chlorpromazine 25mg/1ml solution for injection ampoules
85832998 Aripiprazole 1mg/ml oral solution
85833998 Aripiprazole 15mg orodispersible tablets sugar free
85834998 Aripiprazole 10mg orodispersible tablets sugar free
85835998 Aripiprazole 1mg/ml oral solution

85836998 Aripiprazole 15mg orodispersible tablets sugar free
85837998 Aripiprazole 10mg orodispersible tablets sugar free
86324998 Olanzapine 20mg oral lyophilisates sugar free
86325998 Olanzapine 20mg oral lyophilisates sugar free
86332998 Zuclopenthixol acetate 100mg/2ml oily injection
86333998 Zuclopenthixol acetate 50mg/1ml solution for injection ampoules
86334998 Zuclopenthixol acetate 100mg/2ml solution for injection ampoules
86335998 Zuclopenthixol acetate 50mg/1ml solution for injection ampoules
86420998 Flupentixol 100mg/1ml solution for injection ampoules
86421998 Flupentixol 50mg/0.5ml solution for injection ampoules
86422998 Flupentixol 100mg/1ml solution for injection ampoules
86423998 Flupentixol 50mg/0.5ml solution for injection ampoules
86433998 Amisulpride 25mg/5ml oral suspension
86536998 Flupentixol 100mg/1ml solution for injection ampoules
86537998 Flupentixol 50mg/0.5ml solution for injection ampoules
86539998 Flupentixol 20mg/1ml solution for injection ampoules
86983998 Risperidone 500microgram orodispersible tablets sugar free
86984998 Risperidone 500microgram orodispersible tablets sugar free
87019998 Clozapine 100mg tablets
87020998 Clozapine 25mg tablets
87089998 Aripiprazole 5mg tablets
87090998 Aripiprazole 5mg tablets
87190998 Haloperidol 250micrograms/5ml oral suspension
87340998 Clozapine 100mg tablets
87341998 Clozapine 25mg tablets
87435998 Trifluoperazine 5mg/5ml sugar free suspension
87448998 Aripiprazole 30mg tablets
87449998 Aripiprazole 15mg tablets
87450998 Aripiprazole 10mg tablets
87451998 Aripiprazole 30mg tablets
87452998 Aripiprazole 15mg tablets
87453998 Aripiprazole 10mg tablets
87646998 Olanzapine 10mg injection (powder for reconstitution)
87647998 Olanzapine 10mg powder for solution for injection vials
87907998 Quetiapine 300mg tablets
87908998 Quetiapine 300mg tablets
88163998 Risperidone 37.5mg powder and solvent for suspension for injection vials
88164998 Risperidone 25mg powder and solvent for suspension for injection vials
88383996 Amisulpride 400mg tablets
88383997 Amisulpride 200mg tablets
88383998 Amisulpride 50mg tablets
88387996 Amisulpride 400mg tablets
88387997 Amisulpride 200mg tablets
88387998 Amisulpride 50mg tablets
88733997 Quetiapine 150mg tablets
88733998 Quetiapine 200mg tablets
88734996 Quetiapine 100mg tablets
88734997 Quetiapine 25mg tablets
88734998 Quetiapine 25mg+100mg+150mg tablets starter pack
88736997 Quetiapine 150mg tablets

88736998 Quetiapine 200mg tablets
88737996 Quetiapine 100mg tablets
88737997 Quetiapine 25mg tablets
88737998 Quetiapine starter pack
88885998 Benperidol 250mcg tablets
88924979 Quetiapine 400mg modified-release tablets
88938979 Quetiapine 300mg modified-release tablets
89532979 Aripiprazole 5mg tablets
89567996 Olanzapine 10mg tablets
89567997 Olanzapine 7.5mg tablets
89567998 Olanzapine 5mg tablets
89569996 Olanzapine 10mg tablets
89569997 Olanzapine 7.5mg tablets
89569998 Olanzapine 5mg tablets
89808998 Sertindole 20mg tablets
89809996 Sertindole 16mg tablets
89809997 Sertindole 12mg tablets
89809998 Sertindole 4mg tablets
89811998 Sertindole 20mg tablets
89812996 Sertindole 16mg tablets
89812997 Sertindole 12mg tablets
89812998 Sertindole 4mg tablets
89908998 Risperidone 50mg powder and solvent for suspension for injection vials
90158998 Sulpiride 200mg/5ml oral solution sugar free
90209998 Amisulpride 100mg/ml oral solution sugar free
90395998 Risperidone 1mg orodispersible tablets sugar free
90396998 Risperidone 2mg orodispersible tablets sugar free
90659996 Olanzapine 10mg oral lyophilisates sugar free
90659997 Olanzapine 5mg oral lyophilisates sugar free
90659998 Olanzapine 2.5mg tablets
90664998 Olanzapine 2.5mg tablets
90805998 Sulpiride 200mg tablets
91077998 Amisulpride 100mg tablets
91083998 Amisulpride 100mg tablets
91364998 Olanzapine 15mg oral lyophilisates sugar free
91374998 Risperidone 1mg orodispersible tablets sugar free
91425998 Amisulpride 100mg/ml oral solution sugar free
91618997 Olanzapine 10mg oral lyophilisates sugar free
91618998 Olanzapine 5mg oral lyophilisates sugar free
91676998 Risperidone 25mg powder and solvent for suspension for injection vials
91785990 Amisulpride 50mg tablets
91828990 Olanzapine 20mg oral lyophilisates sugar free
91867990 Olanzapine 5mg oral lyophilisates sugar free
91869990 Olanzapine 15mg tablets
91870990 Olanzapine 10mg tablets
91871990 Olanzapine 7.5mg tablets
91921998 Haloperidol 1mg/ml sugar free Oral solution
91932990 Haloperidol 5mg/5ml oral solution sugar free
91968998 Risperidone 500microgram tablets
92023998 Risperidone 500microgram tablets

92089998 Risperidone 37.5mg powder and solvent for suspension for injection vials
92107998 Risperidone 2mg orodispersible tablets sugar free
92491990 Risperidone 500microgram orodispersible tablets sugar free
92542998 Lithium carbonate 400mg modified release tablets
92623996 Trifluoperazine 2mg modified-release capsules
92623997 Trifluoperazine 1mg/5ml oral solution sugar free
92623998 Trifluoperazine 1mg tablets
92815996 Haloperidol 10mg/5ml oral solution sugar free
92815997 Haloperidol 2mg/5ml oral solution
92815998 Haloperidol 1.5mg/5ml sugar free oral solution
92821997 Thioridazine 100mg/5ml sugar free oral solution
92821998 Thioridazine 50mg/5ml oral solution
92908990 Risperidone 1mg/ml oral solution sugar free
92953990 Risperidone 4mg tablets
92954990 Risperidone 3mg tablets
92955990 Risperidone 2mg tablets
92956990 Risperidone 1mg tablets
92957990 Risperidone 500microgram tablets
93032992 Fluphenazine decanoate 12.5mg/0.5ml solution for injection ampoules
93240996 Risperidone 6mg tablets
93240997 Risperidone 1mg/ml oral solution sugar free
93240998 Risperidone 4mg tablets
93242998 Chlorpromazine hydrochloride 25mg/5ml syrup
93328990 Promazine 25mg tablets
93335997 Remoxipride hydrochloride 300mg modified release capsules
93335998 Remoxipride hydrochloride 150mg modified release capsules
93344997 Remoxipride 300mg capsule
93344998 Remoxipride 150mg capsule
93412998 Lithium carbonate 520mg/5ml sugar free liquid
93476998 Promazine 100mg tablet
93477997 Promazine 50mg tablets
93477998 Promazine 25mg tablets
93519998 Zuclopenthixol acetate 100mg/2ml oily injection
93520998 Zuclopenthixol acetate 50mg/ml oily injection
93587998 Chlorpromazine 100mg suppositories
93590998 Chlorpromazine 25mg/ml injection
93593997 Chlorpromazine 100mg/5ml oral solution
93593998 Chlorpromazine 100mg/5ml oral suspension
93595997 Clozapine 100mg tablets
93595998 Clozapine 25mg tablets
93596997 Clozapine 100mg tablets
93596998 Clozapine 25mg tablets
93674998 Droperidol 1mg/1ml oral liquid
93675998 Droperidol 5mg/ml injection
93695997 Haloperidol 20mg/2ml solution for injection ampoules
93695998 Haloperidol 5mg/ml injection
93699998 Oxypertine 40mg tablets
93708997 Promazine 50mg/ml injection
93708998 Promazine 50mg/ml injection
94006996 Loxapine 50mg capsules

94006997 Loxapine 25mg capsules
94006998 Loxapine 10mg capsules
94007996 Loxapine 50mg capsules
94007997 Loxapine 25mg capsules
94007998 Loxapine 10mg capsules
94107992 Chlorpromazine 50mg tablets
94111992 Chlorpromazine 100mg tablets
94164992 Perphenazine 8mg tablets
94544990 Amisulpride 200mg tablets
94545990 Amisulpride 100mg tablets
94546990 Amisulpride 50mg tablets
94761997 Chlorpromazine 50mg tablets
94761998 Chlorpromazine 25mg tablets
94821992 Chlorpromazine 25mg tablets
94844990 Amisulpride 200mg tablets
94845990 Amisulpride 100mg tablets
94846990 Amisulpride 50mg tablets
94879998 Flupentixol 40mg/2ml solution for injection ampoules
94891992 Dartalan 5 mg tab
95071998 Zuclopenthixol decanoate 500mg/1ml solution for injection ampoules
95086992 Haloperidol 1.5mg tablets
95116997 Trifluperidol 1mg tablets
95116998 Trifluperidol 500mcg tablets
95117997 Trifluperidol 1mg tablet
95117998 Trifluperidol 0.5mg tablet
95118996 Trifluoperazine 5mg/5ml oral solution sugar free
95118997 Trifluoperazine 1mg/ml injection
95118998 Trifluoperazine 15mg modified-release capsules
95119996 Trifluoperazine 10mg modified-release capsules
95119997 Trifluoperazine 5mg tablets
95119998 Trifluoperazine 10mg/ml concentrate
95173996 Thioridazine 25mg/5ml oral solution
95173997 Thioridazine 10mg/5ml oral solution
95173998 Thioridazine 25mg/5ml oral solution
95174996 Thioridazine 100mg/5ml oral suspension
95174997 Thioridazine 25mg/5ml oral suspension
95174998 Thioridazine 100mg tablets
95175996 Thioridazine 50mg tablets
95175997 Thioridazine 25mg tablets
95175998 Thioridazine 10mg tablets
95200992 Chlorpromazine hydrochloride 100mg/5ml sugar free suspension
95226996 Sulpiride 400mg tablets
95226997 Sulpiride 200mg/5ml oral solution sugar free
95226998 Sulpiride 200mg tablets
95242990 Haloperidol 500microgram tablets
95364990 Chlorpromazine 50mg tablets
95365990 Chlorpromazine 25mg tablets
95385997 Promazine 50mg/5ml oral solution
95385998 Promazine 50mg/5ml oral solution sugar free
95386996 Promazine 12.5mg/5ml oral solution

95386997 Promazine 25mg/5ml oral solution
95386998 Promazine 50mg/5ml oral solution
95503998 Pipotiazine palmitate 50mg/ml depot injection
95516996 Pimozide 10mg tablet
95516997 Pimozide 4mg tablets
95516998 Pimozide 2mg tablets
95519998 Risperidone 50mg powder and solvent for suspension for injection vials
95524990 Sulpiride 400mg tablets
95575996 Perphenazine 4mg tablets
95575997 Perphenazine 2mg tablets
95575998 Perphenazine 5mg/ml injection
95576998 Pericyazine 10mg/5ml oral solution
95577996 Pericyazine 25mg tablet
95577997 Pericyazine 10mg tablets
95577998 Pericyazine 2.5mg tablets
95607992 Trifluoperazine 1mg tablets
95649998 Oxypertine 40mg tablets
95650998 Oxypertine 10mg capsules
95687990 Chlorpromazine 25mg/5ml oral solution
95918998 Levomepromazine maleate 25mg tablets
95919997 Levomepromazine 25mg tablets
95919998 Levomepromazine 25mg/1ml solution for injection ampoules
95979998 Benperidol 250microgram tablets
95980998 Benperidol 250microgram tablets
96000996 Lithium citrate 509mg/5ml oral solution
96000997 Lithium citrate 520mg/5ml oral solution sugar free
96000998 Lithium citrate 564mg modified-release tablets
96001998 Lithium carbonate 300mg modified release tablets
96002998 Lithium carbonate 450mg modified-release tablets
96003998 Lithium carbonate 400mg modified-release tablets
96004996 Lithium carbonate 300mg modified-release tablet
96004997 Lithium carbonate 250mg tablets
96004998 Lithium carbonate 200mg modified-release tablets
96102992 Chlorpromazine 50mg/2ml solution for injection ampoules
96115990 Haloperidol 1.5mg tablets
96242997 Haloperidol 10mg/5ml oral liquid
96242998 Haloperidol 5mg/5ml oral solution sugar free
96244998 Haloperidol 500mcg tablets
96245997 Haloperidol decanoate 100mg/1ml solution for injection ampoules
96245998 Haloperidol decanoate 50mg/1ml solution for injection ampoules
96246998 Haloperidol 500microgram capsules
96247996 Haloperidol 1mg/5ml oral solution
96247997 Haloperidol 10mg/ml oral solution
96247998 Haloperidol 10mg/5ml oral solution sugar free
96248996 Haloperidol 5mg/5ml oral solution sugar free
96248997 Haloperidol 20mg tablets
96248998 Haloperidol 10mg tablets
96249996 Haloperidol 5mg tablets
96249997 Haloperidol 1.5mg tablets
96249998 Haloperidol 500microgram tablets

96265992 Haloperidol 20mg tablets
96286990 Fluphenazine decanoate 25mg/1ml solution for injection ampoules
96303997 Droperidol 1mg/ml liquid
96303998 Droperidol 10mg tablets
96307992 Haloperidol decanoate 100mg/1ml solution for injection ampoules
96335979 Lithium carbonate 400mg modified-release tablets
96342992 Fluphenazine decanoate 12.5mg/0.5ml solution for injection ampoules
96395979 Quetiapine 100mg tablets
96402979 Quetiapine 25mg tablets
96421979 Olanzapine 2.5mg tablets
96492992 Thioproperazine mesylate 10 mg tab
96494998 Fluspirilene 2mg/ml injection
96498997 Fluphenazine decanoate 50mg/0.5ml solution for injection ampoules
96498998 Fluphenazine decanoate 25mg/1ml solution for injection ampoules
96500998 Fluphenazine enanthate 25mg/ml injection
96501996 Fluphenazine 5mg tablets
96501997 Fluphenazine 2.5mg tablets
96501998 Fluphenazine 1mg tablets
96502996 Flupentixol 200mg/1ml solution for injection ampoules
96502997 Flupentixol decanoate 100mg/ml injection
96502998 Flupentixol 20mg/1ml solution for injection ampoules
96503998 Flupentixol 3mg tablets
96554979 Risperidone 1mg tablets
96570992 Thioridazine s/f 50 mg/5ml syr
96580979 Trifluoperazine 5mg tablets
96586979 Trifluoperazine 1mg tablets
96614992 Chlorpromazine 100mg/5ml oral suspension
96628997 Zuclopenthixol decanoate 500mg/1ml solution for injection ampoules
96628998 Zuclopenthixol decanoate 200mg/ml oily injection
96629996 Zuclopenthixol 25mg tablets
96629997 Zuclopenthixol 10mg tablets
96629998 Zuclopenthixol 2mg tablets
96634979 Levomepromazine 25mg/1ml solution for injection ampoules
96673979 Chlorpromazine hydrochloride 25mg/5ml syrup
96674979 Chlorpromazine 25mg/5ml oral solution
96686997 Chlorprothixene 50mg tablets
96687979 Chlorpromazine 50mg tablets
96689979 Chlorpromazine 50mg tablets
96689996 Chlorpromazine 100mg tablets
96689997 Chlorpromazine 50mg tablets
96689998 Chlorpromazine 25mg tablets
96690996 Chlorpromazine 25mg/5ml oral solution
96690997 Chlorpromazine 50mg/5ml oral suspension
96690998 Chlorpromazine 100mg/5ml oral solution
96691996 Chlorpromazine 25mg/5ml oral solution
96691997 Chlorpromazine 100mg tablets
96691998 Chlorpromazine 10mg tablets
96701979 Chlorpromazine hydrochloride 25mg tablets
96702979 Chlorpromazine hydrochloride 10mg tablets
96742990 Fluphenazine decanoate 25mg/1ml solution for injection ampoules

96750992 Promazine 25mg tablets
96758992 Haloperidol 20mg/2ml injection
96865992 Lithium citrate 1.018g/5ml oral solution
96889988 Haloperidol 500microgram tablets
96889990 Haloperidol 5mg tablets
96914992 Risperidone 3mg tablets
96919989 Chlorpromazine 100mg tablets
96919990 Chlorpromazine 50mg tablets
96950989 Promazine 50mg/5ml oral solution
96950990 Promazine 25mg/5ml oral solution
97111998 Olanzapine 15mg tablets
97129992 Chlorpromazine hcl 10 mg inj
97131992 Chlorpromazine 25mg suppositories
97132992 Chlorpromazine 50mg/2ml solution for injection ampoules
97134992 Chlorpromazine 200 mg tab
97135989 Haloperidol 1.5mg tablets
97135990 Haloperidol 500microgram tablets
97163989 Sulpiride 400mg tablets
97163990 Sulpiride 200mg tablets
97176998 Sulpiride 200mg tablets
97236988 Chlorpromazine 100mg tablets
97236989 Chlorpromazine 50mg tablets
97236990 Chlorpromazine 25mg tablets
97334992 Droperidol 5 mg/5ml eli
97342996 Pimozide 10mg tablets
97342997 Pimozide 4mg tablets
97342998 Pimozide 2mg tablets
97343997 Droperidol 10mg/2ml injection
97343998 Droperidol 10mg tablets
97344997 Haloperidol decanoate 100mg/1ml solution for injection ampoules
97344998 Haloperidol decanoate 50mg/1ml solution for injection ampoules
97345997 Haloperidol 10mg/2ml injection
97345998 Haloperidol 10mg/ml oral solution
97346996 Haloperidol 10mg/5ml oral solution sugar free
97346997 Haloperidol 10mg tablets
97346998 Haloperidol 5mg tablets
97406989 Promazine 50mg/ml injection
97433998 Olanzapine 15mg tablets
97466992 Fluphenazine hcl eli
97516998 Flupentixol 200mg/1ml solution for injection ampoules
97534998 Lithium citrate 564mg modified release tablets
97568992 Haloperidol 5mg/5ml oral solution sugar free
97673992 Lithium chloride 400 mg sol
97674992 Lithium 250 mg cap
97705997 Lithium citrate 1.018g/5ml oral solution
97705998 Lithium citrate 509mg/5ml oral solution
97715989 Thioridazine 50mg tablets
97715990 Thioridazine 25mg tablets
97716998 Lithium citrate 1.018g/5ml oral solution
97786997 Perphenazine 4mg/5ml oral solution sugar free

97786998 Perphenazine 2mg/5ml oral solution sugar free
97858990 Sulpiride 200mg tablets
97871998 Chlorpromazine hydrochloride 100mg suppositories
97877992 Perphenazine 8 mg tab
97877998 Chlorpromazine hydrochloride 100mg/5ml sugar free suspension
97878992 Pericyazine 2.5 mg eli
97879998 Chlorpromazine 100mg tablets
97880996 Chlorpromazine 50mg tablets
97880997 Chlorpromazine hydrochloride 25mg tablets
97880998 Chlorpromazine hydrochloride 10mg tablets
97944997 Haloperidol 20mg/2ml injection
97944998 Haloperidol 5mg/1ml injection
97945996 Haloperidol 10mg/5ml oral solution sugar free
97945997 Haloperidol 20mg tablets
97945998 Haloperidol 10mg tablets
97946996 Haloperidol 5mg tablets
97946997 Haloperidol 1.5mg tablets
97946998 Haloperidol 500microgram capsules
97951992 Priadel 800 mg tab
97966990 Sulpiride 200mg tablets
97979990 Lithium carbonate 400mg modified-release tablets
97995998 Olanzapine 15mg oral lyophilisates sugar free
98003988 Thioridazine 50mg tablets
98003989 Thioridazine 25mg/5ml oral solution
98003990 Thioridazine 25mg tablets
98052989 Trifluoperazine 1mg tablets
98052990 Trifluoperazine 5mg tablets
98062989 Chlorpromazine 100mg/5ml oral solution
98062990 Chlorpromazine 25mg/5ml oral solution
98063989 Promazine 50mg/5ml oral solution
98063990 Promazine 25mg/5ml oral solution
98080990 Haloperidol 1.5mg/5ml sugar free oral solution
98131989 Haloperidol 5mg/5ml oral solution sugar free
98131990 Haloperidol 1.5mg tablets
98149992 Sulpiride 500 mg tab
98154990 Haloperidol 5mg tablets
98155990 Haloperidol 5mg/1ml solution for injection ampoules
98173992 Thioproperazine mesylate 25 mg tab
98174992 Thiopropazate hcl 10 mg tab
98175992 Thiopropazate hcl 5 mg tab
98186990 Chlorpromazine 50mg/2ml solution for injection ampoules
98189990 Chlorpromazine 25mg tablets
98190996 Zotepine 100mg tablets
98190997 Zotepine 50mg tablets
98190998 Zotepine 25mg tablets
98192988 Chlorpromazine 25mg tablets
98192989 Chlorpromazine 100mg tablets
98192990 Chlorpromazine 50mg tablets
98203992 Trifluoperazine 5mg/5ml oral solution sugar free
98204992 Trifluperidol 2 mg tab

98206992 Trifluoperazine tab
98360988 Haloperidol 1.5mg tablets
98400990 Trifluoperazine 1mg tablets
98403989 Thioridazine 10mg/5ml oral solution
98403990 Thioridazine 25mg/5ml oral solution
98404988 Thioridazine 100mg tablets
98404989 Thioridazine 50mg tablets
98404990 Thioridazine 25mg tablets
98544988 Haloperidol 5mg tablets
98544990 Haloperidol 1.5mg tablets
98585996 Risperidone 3mg tablets
98585997 Risperidone 2mg tablets
98585998 Risperidone 1mg tablets
98587998 Perphenazine 5mg/1ml injection
98622998 Pipotiazine palmitate 100mg/2ml oily injection
98625988 Haloperidol 1mg/5ml oral solution
98625989 Haloperidol 5mg/5ml oral solution sugar free
98625990 Haloperidol 10mg/5ml oral solution sugar free
98668990 Fluphenazine decanoate 100mg/1ml solution for injection ampoules
98759998 Fluphenazine decanoate 100mg/1ml solution for injection ampoules
98766997 Flupentixol 40mg/2ml solution for injection ampoules
98766998 Flupentixol decanoate 20mg/1ml prefilled syringes
98767998 Zuclopenthixol decanoate 200mg/ml oily injection
98783998 Promazine hydrochloride 50mg/5ml suspension
98786996 Promazine 100mg tablet
98786997 Promazine 50mg tablets
98786998 Promazine 25mg tablets
98796997 Sulpiride 400mg tablets
98796998 Sulpiride 200mg tablets
98814998 Lithium carbonate 400mg modified-release tablets
98853997 Levomepromazine 25mg tablets
98853998 Levomepromazine 25mg/1ml solution for injection ampoules
98865998 Pericyazine 10mg/5ml oral solution
98899996 Thioridazine 25mg/5ml syrup
98899997 Thioridazine 100mg/5ml oral suspension
98899998 Thioridazine 25mg/5ml oral suspension
99007990 Chlorpromazine 25mg/5ml oral solution
99010990 Chlorpromazine 50mg tablets
99093988 Promazine 50mg tablets
99093990 Promazine 25mg tablets
99107997 Trifluoperazine 1mg/1ml injection
99107998 Trifluoperazine 15mg modified release capsules
99108996 Trifluoperazine 10mg modified release capsules
99108997 Trifluoperazine 2mg modified release capsules
99108998 Trifluoperazine 10mg/ml concentrate
99109996 Trifluoperazine 1mg/5ml oral solution sugar free
99109997 Trifluoperazine 5mg tablets
99109998 Trifluoperazine 1mg tablets
99117998 Promazine hydrochloride 100mg/2ml injection
99189998 Fluspirilene 12mg/6ml injection

99217997 Lithium carbonate 400mg modified-release tablets
99217998 Lithium carbonate 200mg modified-release tablets
99337996 Zotepine 100mg tablets
99337997 Zotepine 50mg tablets
99337998 Zotepine 25mg tablets
99362996 Pericyazine 25mg tablets
99362997 Pericyazine 10mg tablets
99362998 Pericyazine 2.5mg tablets
99408998 Fluphenazine enantate 25mg/1ml injection
99411996 Fluphenazine hydrochloride 5mg tablets
99411997 Fluphenazine hydrochloride 2.5mg tablets
99411998 Fluphenazine 1mg tablets
99414998 Fluphenazine decanoate 25mg/1ml solution for injection ampoules
99436998 Thioridazine hydrochloride 100mg tablets
99437996 Thioridazine hydrochloride 50mg tablets
99437997 Thioridazine hydrochloride 25mg tablets
99437998 Thioridazine hydrochloride 10mg tablets
99465998 Lithium carbonate 450mg modified-release tablets
99530998 Oxypertine 10mg capsules
99637996 Risperidone 6mg tablets
99637997 Risperidone 1mg/ml oral solution sugar free
99637998 Risperidone 4mg tablets
99649996 Risperidone 3mg tablets
99649997 Risperidone 2mg tablets
99649998 Risperidone 1mg tablets
99651997 Perphenazine 4mg tablets
99651998 Perphenazine 2mg tablets
99775998 Flupentixol decanoate 500mg/5ml oily injection
99776998 Flupentixol 3mg tablets
99821996 Zuclopenthixol 25mg tablets
99821997 Zuclopenthixol 10mg tablets
99821998 Zuclopenthixol 2mg tablets
99858998 Lithium carbonate 250mg tablets

Anti-hypertensives

drugcode	genericname
52835979	Diltiazem 90mg modified-release tablets
52839979	Candesartan 2mg tablets
52855979	Ramipril 2.5mg capsules
53018979	Sildenafil 10mg/ml oral suspension sugar free
53673979	Candesartan 2mg tablets
54852979	MOXONIDINE 200micrograms tabs
54853979	Bisoprolol 2.5mg tablets
56188979	Bisoprolol 1.25mg tablets
58058979	Candesartan 32mg tablets
58096979	Candesartan 8mg tablets
58319979	AZILSARTAN MEDOXOMIL 20mg tabs
58543979	Azilsartan medoxomil 80mg tablets
58544979	AZILSARTAN MEDOXOMIL 40mg tabs
58545979	Azilsartan medoxomil 40mg tablets
58688979	Candesartan 4mg tablets
58692979	BISOPROLOL FUMARATE 2.5mg tabs
58693979	BISOPROLOL FUMARAT 1.25mg tabs
59493979	Bisoprolol 3.75mg tablets
59494979	Bisoprolol 2.5mg tablets
59496979	Bisoprolol 1.25mg tablets
60706979	DILTIAZEM HCL 180mg m/r caps
60707979	DILTIAZEM HCL 90mg m/r caps
61229979	PERINDOPRIL ARGININE 5mg tabs
67971979	Chlorothiazide 250mg/5ml oral solution
78352978	Captopril 5mg/5ml oral solution sugar free
78354978	Captopril 25mg/5ml oral solution sugar free
79827978	Felodipine 2.5mg modified-release tablets
81099998	Ramipril 2.5mg/5ml oral solution sugar free
81128998	Captopril 5mg/5ml oral solution
81242998	METOPROLOL 100mg tablets
81243998	METOPROLOL 50mg tablets
81390998	SILDENAFIL 10mg/12.5mL inj
81391998	Sildenafil 10mg/12.5ml solution for injection vials
81450998	Generic Sevikar HCT 40mg/10mg/25mg tablets
81451998	Generic Sevikar HCT 40mg/5mg/25mg tablets
81453998	Generic Sevikar HCT 40mg/10mg/12.5mg tablets
81454998	Generic Sevikar HCT 40mg/5mg/12.5mg tablets
81456998	Generic Sevikar HCT 20mg/5mg/12.5mg tablets
81563998	BENDROFLUMETHIAZIDE 2.5mg tabs
81611998	Valsartan 3mg/1ml oral solution
81617998	Sildenafil 40mg/50ml solution for injection vials
81728998	Nifedipine 60mg modified-release tablets
81729998	Nifedipine 30mg modified-release tablets
81965998	NIFEDIPINE 60mg m/r tablets
81966998	NIFEDIPINE 30mg m/r tablets
82000998	METHYLDOPA 500mg tablets
82001998	METHYLDOPA 250mg tablets
82102998	Chlorothiazide 250mg tablets

82196998 OLMESAR+HYDROCH 40/12.5mg tabs

82197998 Olmesartan medoxomil 40mg / Hydrochlorothiazide 12.5mg tablets
82426998 Verapamil 240mg modified-release tablets
82427998 VERAPAMIL 120mg m/r tablets
82680998 NIFEDIPINE 20mg m/r tablets
82687998 Nebivolol 2.5mg tablets
82733998 DILTIAZEM HCL 300mg m/r caps
82734998 DILTIAZEM HCL 240mg m/r caps
82735998 DILTIAZEM HCL 180mg m/r caps
82750998 LOSARTAN POTASS 2.5mg/mL susp
82751998 Losartan 2.5mg/ml oral suspension sugar free
82818998 INDAPAMIDE 1.5mg m/r tablets
82870998 INDAPAMIDE 1.5mg m/r tablets
82894998 LOSARTAN POTASSIUM 12.5mg tabs
82895998 Losartan 12.5mg tablets
82910998 OLMESART+AMLODIPN 40/10mg tabs
82911998 OLMESART+AMLODIPNE 40/5mg tabs
82912998 OLMESART+AMLODIPNE 20/5mg tabs

82913998 Olmesartan medoxomil 40mg / Amlodipine 10mg tablets

82914998 Olmesartan medoxomil 40mg / Amlodipine 5mg tablets

82915998 Olmesartan medoxomil 20mg / Amlodipine 5mg tablets
83406998 Indapamide 1.5mg modified-release tablets
83411998 Indapamide 1.5mg modified-release tablets
83435998 LISINO+HYDROCHL 10/12.5mg tabs
83436998 LISINO+HYDROCHL 20/12.5mg tabs
83448998 LISINO+HYDROCHL 20/12.5mg tabs
83449998 LISINO+HYDROCHL 10/12.5mg tabs
83598998 DILTIAZEM HCL 240mg m/r caps
83599998 DILTIAZEM HCL 300mg m/r caps
83605998 DILTIAZEM HCL 240mg m/r caps
83606998 DILTIAZEM HCL 180mg m/r caps
83607998 DILTIAZEM HCL 120mg m/r caps
83610998 DILTIAZEM HCL 120mg m/r tabs
83613998 DILTIAZEM HCL 120mg m/r caps
83614998 DILTIAZEM HCL 90mg m/r caps
83615998 DILTIAZEM HCL 60mg m/r caps
83616998 DILTIAZEM HCL 90mg m/r caps
83617998 DILTIAZEM HCL 180mg m/r caps
83618998 DILTIAZEM HCL 120mg m/r caps
83623998 CAPTOP+HYDROCHL 25/12.5mg tabs
83626998 DILTIAZEM HCL 200mg m/r caps
83627998 DILTIAZEM HCL 300mg m/r caps
83702998 Quinapril 20mg/5ml oral solution
83713998 INDAPAMIDE 1.5mg m/r tablets
83720998 TELMIS+HYDROCHL 80mg/25mg tabs
83721998 Telmisartan 80mg / Hydrochlorothiazide 25mg tablets

83724998 AMBRISENTAN 10mg tablets
83725998 Ambrisentan 10mg tablets
83726998 AMBRISENTAN 5mg tablets
83727998 Ambrisentan 5mg tablets
83832998 RAMIPRIL 2.5mg+5mg+10mg caps
83835998 Ramipril 2.5/5mg/10mg tablet
84035998 PERIND ARG+INDAP 5/1.25mg tabs
84036998 PERINDOPRIL ARGININE 10mg tabs
84037998 PERINDOPRIL ARGININE 5mg tabs
84038998 PERINDOPRIL ARGININE 2.5mg tab
84039998 Perindopril arginine 10mg tablets
84040998 Perindopril arginine 5mg tablets
84041998 Perindopril arginine 2.5mg tablets
84061998 Lisinopril 5mg/5ml oral suspension
84168998 VALSARTAN 320mg tablets
84169998 Valsartan 320mg tablets
84176998 LOSART+HYDRCHL 100/12.5mg tabs

84177998 Hydrochlorothiazide with losartan 12.5mg with 100mg Tablet
84178998 Losartan 100mg / Hydrochlorothiazide 12.5mg tablets
84189998 Felodipine 2.5mg modified-release tablets
84253998 Perindopril arginine 5mg / Indapamide 1.25mg tablets
84299998 Nifedipine 60mg modified-release tablets
84300998 Nifedipine 30mg modified-release tablets
84313998 Irbesartan 300mg/5ml oral suspension
84314998 Enalapril 5mg/5ml oral suspension
84315998 Enalapril 5mg/5ml oral solution
84429998 ALISKIREN 300mg tablets
84430998 ALISKIREN 150mg tablets
84431998 Aliskiren 300mg tablets
84432998 Aliskiren 150mg tablets
84568998 Captopril 5mg tablets
84572998 Captopril 2mg/5ml oral suspension
84649998 FELODIPINE 2.5mg m/r tablets
84734998 Nifedipine Oral solution
84790998 Sitaxentan 100mg tablets
84890998 ILOPROST 10mcg/mL neb soln 1mL
84891998 Iloprost 10micrograms/1ml nebuliser liquid ampoules
84901998 Nifedipine 100mg/5ml oral suspension
84902998 Sildenafil 10mg/5ml oral solution
84922998 Valsartan 160mg with amlodipine 10mg tablets
84924998 Valsartan 160mg with amlodipine 5mg tablets
84925998 Valsartan 80mg with amlodipine 5mg tablets
85014998 AMLODPNE+VALSRTN 10/160mg tabs
85015998 AMLODPNE+VALSARTN 5/160mg tabs
85016998 AMLODIPNE+VALSARTN 5/80mg tabs
85017998 Amlodipine 10mg / Valsartan 160mg tablets
85018998 Amlodipine 5mg / Valsartan 160mg tablets
85019998 Amlodipine 5mg / Valsartan 80mg tablets
85025998 Felodipine 2.5mg modified-release tablets

85218998 IRBES+HYDROCHL 300mg/25mg tabs
85219998 Irbesartan 300mg / Hydrochlorothiazide 25mg tablets
85395998 FELODIPINE 10mg m/r tablets
85396998 FELODIPINE 5mg m/r tablets
85414998 Valsartan 160mg tablets
85416998 Valsartan 80mg tablets
85460998 Captopril 12.5mg/5ml oral suspension
85496998 Ramipril 10mg/5ml oral suspension
85527978 Lisinopril 10mg tablets
85775998 Amlodipine 5mg/5ml oral suspension
85780998 Sotalol 25mg/5ml oral suspension
85788998 CELIPROLOL 400mg tablets
85789998 CELIPROLOL 200mg tablets
85791998 Nifedipine 60mg modified-release tablets
85792998 Nifedipine 30mg modified-release tablets
85844998 Bisoprolol 1.5mg/5ml oral suspension
85850998 Felodipine 2.5mg modified-release tablets
85900998 Nifedipine 10mg/5ml Oral suspension
85959998 OXPRENOLOL 80mg tablets
85960998 OXPRENOLOL 40mg tablets
85961998 OXPRENOLOL 20mg tablets
86017998 Labetalol 100mg/20ml solution for injection ampoules
86031998 NIFEDIPINE 20mg m/r tablets

86039998 Hydrochlorothiazide with olmesartan medoxomil 12.5mg with 20mg tablet

86040998 Hydrochlorothiazide with olmesartan medoxomil 25mg with 20mg tablet
86041998 Chlorothiazide 150mg/5ml oral suspension
86051998 Bisoprolol 5mg/5ml oral suspension
86052998 Nifedipine 20mg modified-release tablets
86053998 LERCANIDIPINE HCl 20mg tablets
86054998 Lercanidipine 20mg tablets
86089998 OLMESART+HYDROCHL 20/25mg tabs
86090998 OLMESAR+HYDROCH 20/12.5mg tabs

86092998 Olmesartan medoxomil 20mg / Hydrochlorothiazide 25mg tablets

86093998 Olmesartan medoxomil 20mg / Hydrochlorothiazide 12.5mg tablets
86098998 BISOPROLOL FUMARATE 2.5mg tabs
86099998 BISOPROLOL FUMARAT 1.25mg tabs
86107998 Diltiazem 180mg modified-release capsules
86108998 Diltiazem 120mg modified-release capsules
86124998 NIFEDIPINE 60mg m/r tablets
86125998 NIFEDIPINE 30mg m/r tablets
86128998 Bendroflumethiazide 2.5mg/5ml oral suspension
86129998 Perindopril erbumine 4mg/5ml oral suspension
86137998 Diltiazem 60mg/5ml oral suspension
86138998 Ramipril 2.5mg/5ml oral suspension
86350998 Lisinopril 7.5mg/5ml oral suspension
86454998 Captopril 6.25mg/5ml oral suspension

86520998 LOSART+HYDROCHLTHZ 100/25 tabs

86521998 Hydrochlorothiazide with losartan 25mg with 100mg Tablet
86522998 Losartan 100mg / Hydrochlorothiazide 25mg tablets
86583998 VERAPAMIL 240mg m/r tablets
86826998 Hydralazine 10mg/5ml oral suspension
86898998 QUINAPRIL 5mg tablets
86929998 Diltiazem 120mg modified-release capsules
86930998 Diltiazem 90mg modified-release capsules
86931998 Diltiazem 60mg modified-release capsules
86973998 NIFEDIPINE 60mg m/r tablets
86974998 NIFEDIPINE 30mg m/r tablets
86989998 Amlodipine 10mg tablets
86990998 Amlodipine 5mg tablets
87016998 Verapamil 240mg modified-release tablets
87021998 FELODIPINE 10mg m/r tablets
87022998 FELODIPINE 5mg m/r tablets
87027998 CANDESARTAN CILEXETL 32mg tabs
87028998 Candesartan 32mg tablets
87064998 Captopril 5mg/5ml oral suspension
87152998 VALSARTAN 40mg tablets
87153998 Valsartan 40mg tablets
87212998 VALSART+HYDROCHL 80/12.5mg tab

87213998 Hydrochlorothiazide with valsartan 12.5mg with 80mg Tablet
87214998 Valsartan 80mg / Hydrochlorothiazide 12.5mg tablets
87226998 RAMIPRIL 1.25mg capsules
87261998 Lisinopril 5mg/5ml oral solution
87288998 TIMOLOL+BENDROFL 10/2.5mg tabs
87290998 TIMOLOL 10mg tablets
87294998 FELODIPINE 10mg m/r tablets
87295998 FELODIPINE 5mg m/r tablets
87297998 AMLODIPINE 10mg tablets
87298998 AMLODIPINE 5mg tablets
87370998 RAMIPRIL 10mg capsules
87371998 RAMIPRIL 5mg capsules
87372998 RAMIPRIL 2.5mg capsules
87421998 VALSART+HYDROCHL 160/25mg tabs

87422998 Hydrochlorothiazide with valsartan 25mg with 160mg Tablet
87424998 Valsartan 160mg / Hydrochlorothiazide 25mg tablets
87426998 VALSRT+HYDROCHL 160/12.5mg tab

87427998 Hydrochlorothiazide with valsartan 12.5mg with 160mg Tablet
87428998 Valsartan 160mg / Hydrochlorothiazide 12.5mg tablets
87446998 FELODIPINE 10mg m/r tablets
87447998 FELODIPINE 5mg m/r tablets
87507998 DILTIAZEM HCL 240mg m/r caps
87508998 DILTIAZEM HCL 180mg m/r caps
87509998 DILTIAZEM HCL 120mg m/r caps

87510998 DILTIAZEM HCL 120mg m/r caps
87511998 DILTIAZEM HCL 90mg m/r caps
87512998 DILTIAZEM HCL 60mg m/r caps
87515998 LISINO+HYDROCHL 20/12.5mg tabs
87539998 Perhexiline maleate 100mg tablet
87572998 Amlodipine maleate 10mg tablets
87573998 Amlodipine maleate 5mg tablets
87621998 TRANDOLAPRIL 4mg capsules
87622998 Trandolapril 4mg capsules
87648998 ILOPROST 10mcg/mL neb soln 2m
87649998 Iloprost 20micrograms/2ml nebuliser liquid ampoules
87653998 FELODIPINE 10mg m/r tablets
87654998 FELODIPINE 5mg m/r tablets
87699998 RAMIPRIL 10mg tablets
87702998 RAMIPRIL 1.25mg tablets
87812998 Captopril 3mg/5ml oral solution
87879998 Felodipine 10mg modified-release tablets
87888998 FELODIPINE 10mg m/r tablets
87889998 FELODIPINE 5mg m/r tablets
87890998 NIFEDIPINE 10mg m/r tablets
87897998 FELODIPINE 10mg m/r tablets
87898998 FELODIPINE 5mg m/r tablets
87899998 RAMIPRIL 10mg tablets
87900998 RAMIPRIL 5mg tablets
87901998 RAMIPRIL 2.5mg tablets
87902998 RAMIPRIL 1.25mg tablets
87903998 Ramipril 10mg tablets
87904998 Ramipril 5mg tablets
87905998 Ramipril 2.5mg tablets
87906998 Ramipril 1.25mg tablets
87955998 CHLOROTHIAZIDE 250mg/5mL susp
87956998 Chlorothiazide 250mg/5ml oral suspension
87963998 Felodipine 5mg modified-release tablets
87969998 DILTIAZEM HCL 120mg m/r caps
87984998 DILTIAZEM HCL 240mg m/r caps
87992997 NIFEDIPINE 20mg m/r tablets
87992998 NIFEDIPINE 10mg m/r tablets
88034998 NIFEDIPINE 10mg m/r tablets
88062998 NIFEDIPINE 20mg m/r tablets
88077996 Atenolol 100mg tablets
88077997 Atenolol 50mg tablets
88077998 Atenolol 25mg tablets
88083997 DILTIAZEM HCL 300mg m/r caps
88083998 DILTIAZEM HCL 240mg m/r caps
88116998 Diltiazem 60mg modified-release capsules
88160998 BISOPROLOL FUMARATE 10mg tabs
88161998 BISOPROLOL FUMARATE 5mg tabs
88234997 NIFEDIPINE 20mg m/r tablets
88234998 NIFEDIPINE 10mg m/r tablets
88290997 DILTIAZEM HCL 300mg m/r caps

88290998 DILTIAZEM HCL 240mg m/r caps
88319996 DILTIAZEM HCL 60mg m/r caps
88319997 DILTIAZEM HCL 120mg m/r caps
88319998 DILTIAZEM HCL 90mg m/r caps
88328998 NIFEDIPINE 30mg m/r tablets
88335998 CANDESARTAN CILEXET 16mg tabs
88349996 ENALAPRIL MALEATE 10mg wafer
88349997 ENALAPRIL MALEATE 5mg wafer
88349998 ENALAPRIL MALEATE 2.5mg wafer
88350998 ENALAPRIL MALEATE 20mg wafer
88410998 DILTIAZEM HCL 60mg m/r tablets
88418997 NIFEDIPINE 60mg m/r tablets
88418998 NIFEDIPINE 30mg m/r tablets
88425998 TRANDOL+VERAP 2/180mg m/r cap

88433998 Trandolapril with verapamil 2mg + 180mg Modified-release capsule

88441998 Verapamil 180mg modified-release / Trandolapril 2mg capsules
88448997 NIFEDIPINE 20mg m/r tablets
88448998 NIFEDIPINE 10mg m/r tablets
88491998 VERAPAMIL 240mg m/r tablets
88506996 IRBESARTAN 300mg tablets
88506997 IRBESARTAN 150mg tablets
88506998 IRBESARTAN 75mg tablets
88507996 Irbesartan 300mg tablets
88507997 Irbesartan 150mg tablets
88507998 Irbesartan 75mg tablets
88508996 Captopril 50mg tablets
88508998 Captopril 12.5mg tablets
88541998 LERCANIDIPINE HCL 10mg tablets
88551997 NIFEDIPINE 20mg m/r tablets
88714998 Generic Tritace titration pack capsules
88837996 DILTIAZEM HCL 120mg m/r caps
88837997 DILTIAZEM HCL 90mg m/r caps
88837998 DILTIAZEM HCL 60mg m/r caps
88877997 NIFEDIPINE 20mg m/r tablets
88877998 NIFEDIPINE 10mg m/r tablets
88884998 VERAPAMIL 120mg m/r tablets
88945996 DILTIAZEM HCL 180mg m/r caps
88945997 DILTIAZEM HCL 120mg m/r caps
88945998 DILTIAZEM HCL 300mg m/r caps
88955998 PERIND ERB+INDAP 4/1.25mg tabs
88995979 DILTIAZEM HCL 120mg m/r tabs
88996979 Diltiazem 120mg modified-release tablets
89020998 NIFEDIPINE 20mg m/r tablets
89024998 VERAPAMIL 240mg m/r tablets
89057997 Mibefradil 100mg Tablet
89057998 Mibefradil 50mg Tablet
89058996 CAPTOPRIL 50mg tablets
89058997 CAPTOPRIL 25mg tablets x56

89058998 CAPTOPRIL 12.5mg tablets
89061997 Co-zidocapt 12.5mg/25mg tablets
89061998 Co-zidocapt 25mg/50mg tablets
89067998 VERAPAMIL 240mg m/r tablets
89069996 CAPTOPRIL 50mg tablets
89069997 CAPTOPRIL 25mg tablets x56
89069998 CAPTOPRIL 12.5mg tablets
89085997 DILTIAZEM HCL 360mg m/r caps
89085998 DILTIAZEM HCL 300mg m/r caps
89087996 DILTIAZEM HCL 240mg m/r caps
89087997 DILTIAZEM HCL 180mg m/r caps
89087998 DILTIAZEM HCL 120mg m/r caps
89102997 CAPTOPRIL 25mg tablets
89102998 CAPTOPRIL 12.5mg tablets
89103998 NIFEDIPINE 20mg m/r tablets
89145998 NIFEDIPINE 20mg m/r tablets
89186998 NIFEDIPINE 10mg m/r tablets
89190997 MIBEFRADIL 100mg tablets
89190998 MIBEFRADIL 50mg tablets
89237998 INDAPAMIDE 1.5mg m/r tablets
89282996 EPROSARTAN 600mg tablets
89282997 EPROSARTAN 400mg tablets
89282998 EPROSARTAN 300mg tablets
89283996 Eprosartan 600mg tablets
89283997 Eprosartan 400mg tablets
89283998 Eprosartan 300mg tablets
89288998 ATENOLOL+BENDRO 25/1.25mg cap
89290998 Atenolol 25mg / Bendroflumethiazide 1.25mg capsules
89292997 IRBES+HYDROCHL 300/12.5mg tabs
89292998 IRBES+HYDROCHL 150/12.5mg tabs

89305998 Perindopril erbumine 4mg / Indapamide 1.25mg tablets
89396998 CARVEDILOL 6.25mg tablets
89397998 Carvedilol 6.25mg tablets
89424998 PERINDOPRIL ERBUMINE 8mg tabs
89459998 NIFEDIPINE 40mg m/r tablets
89513996 VALSARTAN 160mg capsules
89513997 VALSARTAN 80mg capsules
89513998 VALSARTAN 40mg capsules
89514996 Valsartan 160mg capsules
89514997 Valsartan 80mg capsules
89514998 Valsartan 40mg capsules
89519996 NISOLDIPINE 30mg m/r tablets
89519997 NISOLDIPINE 20mg m/r tablets
89519998 NISOLDIPINE 10mg m/r tablets
89522996 Nisoldipine 30mg modified-release tablets
89522997 Nisoldipine 20mg modified-release tablets
89522998 Nisoldipine 10mg modified-release tablets
89543998 Enalapril 20mg tablets
89618998 NIFEDIPINE 40mg m/r tablets

89652997 NIFEDIPINE 20mg m/r tablets
89652998 NIFEDIPINE 10mg m/r tablets
89656996 BISOPROLOL FUMARATE 10mg tabs
89656997 BISOPROLOL FUMARATE 7.5mg tabs
89656998 BISOPROLOL FUMARATE 5mg tabs
89658996 BISOPROLOL FUMARAT 3.75mg tabs
89658997 BISOPROLOL FUMARATE 2.5mg tabs
89658998 BISOPROLOL FUMARAT 1.25mg tabs
89659996 Bisoprolol 7.5mg tablets
89659997 Bisoprolol 3.75mg tablets
89659998 Bisoprolol 2.5mg tablets
89704997 DILTIAZEM HCL 120mg m/r tabs
89704998 DILTIAZEM HCL 90mg m/r tablets
89768997 NIFEDIPINE 20mg m/r tablets
89768998 NIFEDIPINE 10mg m/r tablets
90049998 Captopril 2mg tablets
90077998 LOSART+HYDROCHLTHZ 50/12.5 tab
90181998 DILTIAZEM HCL 90mg m/r caps
90182998 DILTIAZEM HCL 60mg m/r caps
90210998 DILTIAZEM HCL 200mg m/r caps
90294998 VERAPAMIL 40mg/5mL s/f soln
90432996 TELMISARTAN 20mg tablets
90432997 TELMISARTAN 80mg tablets
90432998 TELMISARTAN 40mg tablets

90433998 Diltiazem and hydrochlorothiazide 150mg+12.5mg modified-release capsules
90434998 DIL+HYDROCHLOR 150/12.5mg cap
90503996 Candesartan 8mg tablets
90503997 Candesartan 4mg tablets
90503998 Candesartan 2mg tablets
90504998 Candesartan 16mg tablets
90533996 IMIDAPRIL HCL 20mg tablets
90533997 IMIDAPRIL HCL 10mg tablets
90533998 IMIDAPRIL HCL 5mg tablets
90547998 Telmisartan 40mg / Hydrochlorothiazide 12.5mg tablets
90549998 TELMIS+HYDROCHL 40/12.5mg tabs
90559998 NIFEDIPINE 30mg m/r tablets
90576996 Imidapril 20mg tablets
90576997 Imidapril 10mg tablets
90576998 Imidapril 5mg tablets
90609996 MOXONIDINE 300micrograms tabs
90609997 MOXONIDINE 400micrograms tabs
90609998 MOXONIDINE 200micrograms tabs
90613996 Moxonidine 300microgram tablets
90613997 Moxonidine 400microgram tablets
90613998 Moxonidine 200microgram tablets
90629996 DILTIAZEM HCL 300mg m/r caps
90629997 DILTIAZEM HCL 240mg m/r caps
90706996 DILTIAZEM HCL 120mg m/r caps
90706997 DILTIAZEM HCL 90mg m/r caps

90706998 DILTIAZEM HCL 60mg m/r caps
90722996 Nifedipine 40mg modified-release tablets
90722997 Nifedipine 60mg modified-release tablets
90722998 Nifedipine 24 30mg Modified-release tablet
90740998 CAPTOP+HYDROCHL 25/12.5mg tabs
90781998 DILTIAZEM HCL 60mg m/r tablets
90789998 LOSARTAN POTASSIUM 100mg tabs
90816997 Moexipril 15mg tablets
90816998 Moexipril 7.5mg tablets
90817997 MOEXIPRIL HCL 15mg tablets
90817998 MOEXIPRIL HCL 7.5mg tablets
90918998 NEBIVOLOL 5mg tablets
90919998 Nebivolol 5mg tablets
90981998 NIFEDIPINE 10mg m/r tablets
91065998 Ramipril 2.5/5mg/10mg capsule
91079997 CO-TENIDONE 100/25mg tablets
91079998 CO-TENIDONE 50/12.5mg tablets
91085998 DILTIAZEM HCL 300mg m/r caps
91089997 Irbesartan 300mg / Hydrochlorothiazide 12.5mg tablets
91089998 Irbesartan 150mg / Hydrochlorothiazide 12.5mg tablets
91145997 Nifedipine 60mg modified-release capsules
91145998 Nifedipine 30mg modified-release capsules
91168997 DILTIAZEM HCL 90mg m/r tablets
91168998 DILTIAZEM HCL 120mg m/r tabs
91240998 Losartan 50mg / Hydrochlorothiazide 12.5mg tablets
91270998 NIFEDIPINE 20mg m/r tablets
91344998 Lercanidipine 10mg tablets
91358996 DILTIAZEM HCL 180mg m/r caps
91358997 DILTIAZEM HCL 120mg m/r caps
91358998 DILTIAZEM HCL 90mg m/r caps
91422997 NIFEDIPINE 5mg capsules
91422998 NIFEDIPINE 10mg capsules
91444998 Perindopril erbumine 8mg tablets
91491998 SOTALOL 40mg/4mL injection
91518998 DILTIAZEM HCL 240mg m/r caps
91520998 Losartan 100mg tablets
91565997 NIFEDIPINE 60mg m/r capsules
91565998 NIFEDIPINE 30mg m/r capsules
91571996 CANDESARTAN CILEXETIL 8mg tabs
91571997 CANDESARTAN CILEXETIL 4mg tabs
91571998 CANDESARTAN CILEXETIL 2mg tabs

91577998 Hydrochlorothiazide with losartan 12.5mg with 50mg Tablet
91607998 INDAPAMIDE 2.5mg tablets
91779990 Bisoprolol 1.25mg tablets
91784998 FELODIPINE 2.5mg m/r tablets
91796998 VERAPAMIL 240mg m/r tablets
91810998 VERAPAMIL 240mg m/r tablets
91818998 INDAPAMIDE 2.5mg tablets
91861998 Olmesartan medoxomil 20mg tablets

91867998 OLMESARTAN MEDOXOMIL 10mg tabs
91868998 OLMESARTAN MEDOXOMIL 20mg tabs
91872998 Olmesartan medoxomil 10mg tablets
91873998 Olmesartan medoxomil 40mg tablets
91884998 PINDOLOL 5mg tablets
91887998 PINDOLOL 15mg tablets
91958996 Enalapril 20mg wafer
91958997 Enalapril 10mg wafer
91958998 Enalapril 5mg wafer
92013990 Bisoprolol 1.25mg tablets
92042997 NIFEDIPINE 20mg m/r tablets
92042998 NIFEDIPINE 10mg m/r tablets
92094998 Methyldopa 250mg Capsule
92151998 BISOPROLOL FUMARATE 10mg tabs
92161990 Lercanidipine 10mg tablets
92181990 Lercanidipine 20mg tablets
92202998 DILTIAZEM HCL 300mg m/r caps
92204997 DILTIAZEM HCL 300mg m/r caps
92204998 DILTIAZEM HCL 240mg m/r caps
92341998 Diltiazem 60mg modified-release capsules
92343990 Nebivolol 2.5mg tablets
92354998 FELODIPINE 10mg m/r tablets
92355998 FELODIPINE 5mg m/r tablets
92439990 Perindopril erbumine 4mg tablets
92440990 Perindopril erbumine 2mg tablets
92516997 Losartan 25mg tablets
92516998 Losartan 50mg tablets
92517997 LOSARTAN POTASSIUM 25mg tabs
92517998 LOSARTAN POTASSIUM 50mg tabs
92527996 DILTIAZEM HCL 240mg m/r caps
92527997 DILTIAZEM HCL 180mg m/r caps
92527998 DILTIAZEM HCL 120mg m/r caps
92588998 NIFEDIPINE 20mg m/r tablets
92627996 DILTIAZEM HCL 240mg m/r caps
92627997 DILTIAZEM HCL 180mg m/r caps
92627998 DILTIAZEM HCL 120mg m/r caps
92663990 Amlodipine 5mg tablets
92670998 INDAPAMIDE 2.5mg tablets
92687990 Indapamide 1.5mg modified-release tablets
92702990 Verapamil 120mg modified-release tablets
92727997 Diltiazem 360mg modified-release capsules
92727998 Diltiazem 24hr 300mg modified-release capsules
92731998 Quinapril 40mg tablets
92732998 QUINAPRIL 40mg tablets
92736997 BENDROFLUMETHIAZIDE 5mg tablet
92736998 BENDROFLUMETHIAZIDE 2.5mg tabs
92753996 Diltiazem 240mg modified-release capsules
92753997 DILTIAZEM HCL 180mg m/r caps
92753998 Diltiazem 24hr 120mg modified-release capsules
92754996 Nifedipine extra 60mg Modified-release tablet

92754997 Nifedipine extra 30mg Modified-release tablet
92754998 Nifedipine 24 20mg Modified-release tablet
92762997 NIFEDIPINE 10mg m/r tablets
92762998 NIFEDIPINE 20mg m/r tablets
92810990 BISOPROLOL FUMARAT 1.25mg tabs
92824996 DILTIAZEM HCL 240mg m/r caps
92824997 DILTIAZEM HCL 180mg m/r caps
92824998 DILTIAZEM HCL 120mg m/r caps
92826998 Verapamil 40mg/5ml oral solution sugar free
92835990 Amlodipine 5mg tablets
92849996 DILTIAZEM HCL 240mg m/r caps
92849997 DILTIAZEM HCL 180mg m/r caps
92849998 DILTIAZEM HCL 120mg m/r caps
92885990 Perindopril erbumine 8mg tablets
92886990 Perindopril erbumine 4mg tablets
92887990 Perindopril erbumine 2mg tablets
92887996 Enalapril 10mg tablets
92887997 Enalapril 5mg tablets
92887998 Enalapril 2.5mg tablets
92889998 Bisoprolol 10mg / Hydrochlorothiazide 6.25mg tablets
92890998 BISOPROLOL+HYDROCHLOROTH tabs
92926998 OXPRENOLOL 160mg m/r tablets
92960990 Perindopril erbumine 4mg tablets
92968997 Lacidipine 4mg tablets
92968998 Lacidipine 2mg tablets
92969997 LACIDIPINE 4mg tablets
92969998 LACIDIPINE 2mg tablets
92990996 Telmisartan 20mg tablets
92990997 Telmisartan 80mg tablets
92990998 Telmisartan 40mg tablets
93013992 NIFEDIPINE 20mg m/r tablets
93014996 DILTIAZEM HCL 120mg m/r caps
93014997 DILTIAZEM HCL 90mg m/r caps
93014998 DILTIAZEM HCL 60mg m/r caps
93076990 Atenolol 50mg tablets
93101998 Quinapril 10mg / Hydrochlorothiazide 12.5mg tablets
93102998 QUINAPRIL+HYDROCHLOROTHIAZIDE
93133998 Nimodipine 30mg tablets
93136997 Fosinopril 20mg tablets
93136998 Fosinopril 10mg tablets
93137997 FOSINOPRIL 20mg tablets
93137998 FOSINOPRIL 10mg tablets
93149990 Bisoprolol 1.25mg tablets
93199997 Guanethidine 25mg Tablet
93199998 Guanethidine 10mg Tablet
93204998 RESERPINE 100mcg tablets
93209998 Co-prenozide 160mg/0.25mg modified-release tablets

93210998 Oxprenolol with cyclopenthiazide 160mg+0.25mg Modified-release tablet
93219997 Co-tenidone 100mg/25mg tablets

93219998 Co-tenidone 50mg/12.5mg tablets
93232997 Nifedipine 10mg modified-release capsules
93232998 Nifedipine 20mg modified-release capsules
93249992 NIFEDIPINE TAB 5 mg
93251997 NIFEDIPINE 10mg m/r capsules
93251998 NIFEDIPINE 20mg m/r capsules
93295992 NADOLOL 80MG/BENDROFLUAZIDE 5MG MG TAB
93306990 Bisoprolol 1.25mg tablets
93329990 Perindopril erbumine 8mg tablets
93330990 Perindopril erbumine 4mg tablets
93331990 Perindopril erbumine 2mg tablets
93342990 Bisoprolol 2.5mg tablets
93344992 CO-TENIDONE 100/25mg tablets
93345992 CO-TENIDONE 50/12.5mg tablets
93392990 Nifedipine 60mg modified-release tablets
93396990 Nifedipine 24 30mg Modified-release tablet
93397992 CATAPRES 15 MCG/ML INJ
93459996 ENALAPRIL MAL 10mg tabs x28
93459997 ENALAPRIL MALEATE 5mg tabs x2
93523997 Amlodipine besilate 10mg tablets
93523998 Amlodipine besilate 5mg tablets
93524997 Amlodipine 10mg tablets
93524998 Amlodipine 5mg tablets
93561996 ATENOLOL 25mg tablets
93561997 ATENOLOL 100mg tablets
93561998 ATENOLOL 50mg tablets
93562996 ATENOLOL 25mg tablets
93562997 ATENOLOL 100mg tablets
93562998 ATENOLOL 50mg tablets
93563998 ATENOLOL 50mg tablets
93567996 Ramipril 5mg capsules
93567997 Ramipril 2.5mg capsules
93567998 Ramipril 1.25mg capsules
93568996 RAMIPRIL 5mg capsules
93568997 RAMIPRIL 2.5mg capsules
93568998 RAMIPRIL 1.25mg capsules
93597997 Perindopril erbumine 4mg tablets
93597998 Perindopril erbumine 2mg tablets
93598997 PERINDOPRIL ERBUMINE 4mg tabs
93598998 PERINDOPRIL ERBUMINE 2mg tabs
93599996 Diltiazem 240mg modified-release capsules
93599997 Diltiazem 90mg modified-release tablets
93599998 Diltiazem 120mg modified-release tablets
93600996 DILTIAZEM HCL 90mg m/r caps
93600997 DILTIAZEM HCL 60mg m/r tablets
93600998 DILTIAZEM HCL 120mg m/r tabs
93608998 ATENOLOL 25mg tablets
93659998 Clonidine 250microgram modified-release capsules
93690992 CLOREXOLONE 10 MG TAB
93691996 CARVEDILOL 3.125mg tablets

93691997 CARVEDILOL 25mg tablets
93691998 CARVEDILOL 12.5mg tablets
93692996 Carvedilol 3.125mg tablets
93692997 Carvedilol 25mg tablets
93692998 Carvedilol 12.5mg tablets

93694998 Guanethidine 10mg/1ml solution for injection ampoules
93699992 HYDRALAZINE HCl 10 MG TAB
93710997 Diltiazem 200mg modified-release capsules
93710998 Diltiazem 300mg modified-release capsules
93727992 RAMIPRIL 1.25mg tablets
93748997 LISINO+HYDROCHL 10/12.5mg tabs
93748998 LISINO+HYDROCHL 20/12.5mg tabs
93772998 Diltiazem 60mg modified-release tablets
93776990 Verapamil 240mg modified-release tablets
93798992 LABETALOL TAB
93846998 METOLAZONE 500mcg tablets
93850990 Acebutolol 400mg tablets
93851990 Acebutolol 200mg capsules
93896990 Lisinopril 10mg tablets
93905998 Carteolol HCl 10mg tablets
93928996 TRANDOLAPRIL 2mg capsules
93928997 TRANDOLAPRIL 1mg capsules
93928998 TRANDOLAPRIL 500mcg capsules
93929998 VERAPAMIL 240mg m/r tablets
93937998 CARTEOLOL 10mg tablets
94040992 RAMIPRIL 2.5mg tablets
94050992 DILTIAZEM HCL 120mg m/r tabs
94055997 NIFEDIPINE 10mg m/r tablets
94055998 NIFEDIPINE 20mg m/r tablets
94082998 VERAPAMIL 5mg/2mL injection
94101992 CHLOROTHIAZIDE 25 MG LIQ
94111998 DILTIAZEM HCL 60mg m/r tablets
94118998 NIFEDIPINE 10mg capsules
94139998 Ramipril 10mg capsules
94145996 NIFEDIPINE 5mg capsules
94145998 NIFEDIPINE 10mg capsules
94182992 HYDRALAZINE 1 MG SYR
94243992 NATRILIX 5 MG TAB
94258992 OXPRENOLOL 10 MG TAB
94275992 PERHEXILINE MALEATE 100 MG TAB
94341990 Amlodipine 5mg tablets
94361990 Ramipril 2.5mg capsules
94376990 DILTIAZEM HCL 240mg m/r caps
94395996 Quinapril 20mg tablets
94395997 Quinapril 10mg tablets
94395998 Quinapril 5mg tablets
94396996 QUINAPRIL 20mg tablets
94396997 QUINAPRIL 10mg tablets
94396998 QUINAPRIL 5mg tablets

94419992	BENDROFLUAZIDE 1 MG TAB
94465998	Verapamil 160mg tablets
94474998	Isradipine 2.5mg tablets
94475998	ISRADIPINE 2.5mg tablets
94497998	METOPROLOL FUMARATE 95mg tabs
94509990	Ramipril 10mg capsules
94510990	Ramipril 5mg capsules
94511990	Ramipril 2.5mg capsules
94520996	DILTIAZEM HCL 180mg m/r caps
94520997	DILTIAZEM HCL 120mg m/r caps
94520998	DILTIAZEM HCL 90mg m/r caps
94541992	RESERPINE/BENDROFLUAZIDE 150 MCG TAB
94545998	Amiloride with atenolol with hydrochlorothiazide capsules
94548990	Moxonidine 300microgram tablets
94549998	Hydrochlorothiazide with timolol and amiloride 25mg with 10mg with 2.5mg Tablet
94550998	Amiloride with timolol with hydrochlorothiazide tablets
94551998	Timolol maleate with amiloride and hydrochlorothiazide Tablet
94553997	Hydrochlorothiazide with captopril 12.5mg with 25mg Tablet
94553998	Hydrochlorothiazide with captopril 25mg with 50mg Tablet
94561990	Lisinopril 10mg tablets
94573998	Timolol 10mg tablets
94603996	Trandolapril 2mg capsules
94603997	Trandolapril 1mg capsules
94603998	Trandolapril 500microgram capsules
94616990	Moxonidine 200microgram tablets
94633990	Ramipril 10mg capsules
94633997	Hydrochlorothiazide with metoprolol tartrate 12.5mg with 100mg tablet
94633998	Hydrochlorothiazide with metoprolol tartrate 25mg with 200mg Modified-release tablet
94634997	Metoprolol 100mg / Hydrochlorothiazide 12.5mg tablets
94634998	Metoprolol 200mg modified-release / Hydrochlorothiazide 25mg tablets
94635998	Metoprolol tartrate with chlortalidone Tablet
94636990	Ramipril 1.25mg capsules
94637997	CELIPROLOL 400mg tablets
94637998	CELIPROLOL 200mg tablets
94668998	Nifedipine with atenolol 20mg + 50mg Capsule
94669998	Atenolol 50mg / Nifedipine 20mg modified-release capsules
94670998	Atenolol with amiloride and hydrochlorothiazide capsules

94671998 Hydrochlorothiazide with atenolol and amiloride Capsule
94672997 Celiprolol 400mg tablets
94672998 Celiprolol 200mg tablets
94679996 ATENOLOL 25mg tablets
94679997 ATENOLOL 100mg tablets
94679998 ATENOLOL 50mg tablets
94682992 ATENOLOL/CHLORTHALIDONE 50 MG TAB
94701992 BENDROFLUAZIDE 40 MG TAB
94702992 BENDROFLUAZIDE 10 MG TAB
94713990 Ramipril 10mg tablets
94716998 DILTIAZEM HCL 180mg m/r caps
94717990 Ramipril 5mg tablets
94719990 Ramipril 2.5mg tablets
94726998 ATENOLOL+NIFEDIP 50/20mg m/r
94732998 ATENOLOL+NIFEDIP 50/20mg m/r
94739998 DILTIAZEM HCL 60mg m/r tablets
94740997 Diltiazem 60mg modified-release capsules
94740998 Diltiazem 60mg tablets
94741997 NIMODIPINE 30mg tablets
94748998 Lisinopril 20mg tablets
94749990 Diltiazem 90mg modified-release capsules
94749996 Lisinopril 10mg tablets
94749997 Lisinopril 5mg tablets
94749998 Lisinopril 2.5mg tablets
94750998 LISINOPRIL 20mg tablets
94751996 LISINOPRIL 10mg tablets
94751997 LISINOPRIL 5mg tablets
94751998 LISINOPRIL 2.5mg tablets
94752998 LISINOPRIL 20mg tablets
94753996 LISINOPRIL 10mg tablets
94753997 LISINOPRIL 5mg tablets
94753998 LISINOPRIL 2.5mg tablets
94756990 Moxonidine 400microgram tablets
94757990 Moxonidine 300microgram tablets
94758990 Moxonidine 200microgram tablets
94766996 Nifedipine 60mg Modified-release tablet
94766997 Nifedipine 30mg modified-release tablets
94766998 Nifedipine 10mg modified-release tablets
94770990 Amlodipine 5mg tablets
94774996 VERAPAMIL 240mg m/r capsules
94774997 VERAPAMIL 180mg m/r capsules
94774998 VERAPAMIL 120mg m/r capsules
94775996 Verapamil 240mg modified-release capsules
94775997 Verapamil 180mg modified-release capsules
94775998 Verapamil 120mg modified-release capsules
94783996 Bisoprolol 1.25mg tablets
94783997 Bisoprolol 10mg tablets
94783998 Bisoprolol 5mg tablets
94792990 Amlodipine 10mg tablets

94802998 BISOPROLOL FUMARATE 5mg tabs
94803992 CHLOROTHIAZIDE 50 MG SUS
94804992 CHLOROTHIAZIDE SACHETS 60 MG
94804998 BISOPROLOL FUMARATE 10mg tabs
94808997 BISOPROLOL FUMARATE 10mg tabs
94808998 BISOPROLOL FUMARATE 5mg tabs
94810998 Verapamil 5mg/2ml solution for injection ampoules
94811992 CHLOROTHIAZIDE 250 MG SYR
94813997 Labetalol 400mg tablets
94813998 Labetalol 50mg tablets
94833990 Ramipril 10mg capsules
94834990 Ramipril 5mg capsules
94860998 Diltiazem 60mg modified-release tablets
94867996 TRANDOLAPRIL 2mg capsules
94867997 TRANDOLAPRIL 1mg capsules
94867998 TRANDOLAPRIL 500mcg capsules
94868997 CAPTOP+HYDROCHL 25/12.5mg tabs
94868998 CAPTOP+HYDROCHL 50/25mg tabs
94869990 Diltiazem 60mg modified-release tablets

94869997 Captopril 25mg with Hydrochlorothiazide 12.5mg tablets
94869998 Captopril 50mg with Hydrochlorothiazide 25mg tablets
94876990 Amlodipine 5mg tablets
94889990 Bendroflumethiazide 2.5mg tablets
94924992 DILTIAZEM HCL S/R 180 CAP
94929997 Atenolol 25mg tablets
94929998 Atenolol 25mg/5ml oral solution sugar free
94931990 Carvedilol 3.125mg tablets
94949990 Lisinopril 20mg / Hydrochlorothiazide 12.5mg tablets
94958990 Bisoprolol 5mg tablets
94958992 ENDURONYL TAB
94967990 Carvedilol 3.125mg tablets
94972990 Ramipril 1.25mg capsules
94982990 Amlodipine 5mg tablets
94984990 Carvedilol 12.5mg tablets
94985990 Carvedilol 6.25mg tablets
94986990 Carvedilol 3.125mg tablets
94989990 Carvedilol 12.5mg tablets
94990990 Carvedilol 6.25mg tablets
94991990 Carvedilol 3.125mg tablets
94992990 Lisinopril 20mg / Hydrochlorothiazide 12.5mg tablets
94993990 Lisinopril 10mg / Hydrochlorothiazide 12.5mg tablets
94995990 Amlodipine 5mg tablets
95010990 Amlodipine 10mg tablets
95011990 Amlodipine 5mg tablets

95011998 Bendroflumethiazide 2.5mg with Timolol maleate 10mg tablets
95012997 Bendroflumethiazide 5mg with Nadolol 80mg tablets
95012998 Bendroflumethiazide 5mg with Nadolol 40mg tablets

95014997 Bendroflumethiazide 5mg with Propanolol 160mg modified-release capsules

95014998 Bendroflumethiazide 2.5mg with Propanolol 80mg capsules
95021997 Atenolol 100mg with Chlortalidone 25mg tablets
95021998 Atenolol 50mg with Chlortalidone 12.5mg tablets
95042990 Ramipril 10mg capsules
95043990 Ramipril 5mg capsules
95044990 Ramipril 2.5mg capsules
95058990 Ramipril 10mg capsules
95059990 Ramipril 5mg capsules
95061990 Ramipril 1.25mg capsules
95088992 HARMONYL .25 MG TAB
95100990 Ramipril 10mg capsules
95101990 Ramipril 5mg capsules
95102990 Ramipril 2.5mg capsules
95103990 Ramipril 1.25mg capsules
95112992 HYDRALAZINE 6.25 MG SYR
95114992 HYDRALAZINE HCl 12.5 MG TAB

95120992 HYDROCHLOROTHIAZIDE /METOPROLOL TARTRATE 25 MG TAB
95127990 Ramipril 10mg capsules
95128990 Ramipril 5mg capsules
95129990 Ramipril 2.5mg capsules
95133990 Ramipril 10mg capsules
95134990 Ramipril 5mg capsules
95135990 Ramipril 2.5mg capsules
95136990 Ramipril 10mg capsules
95137990 Ramipril 5mg capsules
95138990 Ramipril 2.5mg capsules
95140998 Metoprolol 50mg/5ml oral suspension
95145990 Ramipril 10mg capsules
95146990 Ramipril 5mg capsules
95147990 Ramipril 5mg tablets
95148990 Ramipril 2.5mg tablets
95149990 Ramipril 2.5mg capsules
95150990 Ramipril 1.25mg capsules
95159998 TIMOLOL+BENDROFL 20/5mg tabs

95161997 Timolol maleate with bendroflumethiazide 20mg + 5mg Tablet
95161998 Timolol 10mg / Bendroflumethiazide 2.5mg tablets
95209990 Felodipine 5mg modified-release tablets
95253990 Labetalol 400mg tablets
95264998 SOTAL+HYDROCHLO 80/12.5mg tab
95265998 SOTALOL 40mg/4mL injection
95267997 Sotalol 160mg with hydrochlorothiazide 25mg tablet
95267998 Sotalol 80mg with hydrochlorothiazide 12.5mg tablet
95268997 Sotalol 2mg/ml injection
95268998 Sotalol 10mg/ml injection
95269998 Sotalol 200mg tablets

95270996 Sotalol 160mg tablets
95270997 Sotalol 80mg tablets
95270998 Sotalol 40mg tablets
95276992 METHYLDOPA 200 MG TAB

95276998 Sodium nitroprusside 50mg powder for solution for infusion vials
95321990 Bendroflumethiazide 2.5mg tablets
95324998 Reserpine with hydrochlorothiazide tablet
95325997 Reserpine 250micrograms tablet
95325998 Reserpine 100micrograms tablet
95328990 Felodipine 10mg modified-release tablets
95329990 Felodipine 5mg modified-release tablets
95329998 RAUWOLFIA ALKALOIDS 2mg tabs
95421998 Practolol 2mg/ml injection
95453990 Bendroflumethiazide 2.5mg tablets
95465990 Verapamil 240mg modified-release tablets
95468990 Metoprolol 50mg tablets
95469998 Polythiazide 1mg tablets
95505990 Sotalol 40mg tablets
95514998 Pindolol 10mg / Clopamide 5mg tablets
95515997 Pindolol 15mg tablets
95515998 Pindolol 5mg tablets
95540992 RAUTRAX 50 MG TAB
95572992 VERAPAMIL 120mg tablets
95588990 Verapamil 80mg tablets
95602992 SOTALOL HCl S/R 80 MG TAB
95616998 Xipamide 20mg tablets
95626997 Verapamil 120mg modified-release tablets
95626998 Verapamil 240mg modified-release tablets
95628996 Verapamil 120mg tablets
95628997 Verapamil 80mg tablets
95628998 Verapamil 40mg tablets
95631996 FELODIPINE 2.5mg m/r tablets
95631997 FELODIPINE 10mg m/r tablets
95631998 FELODIPINE 5mg m/r tablets
95635990 Co-zidocapt 25mg/50mg tablets
95651990 Sotalol 40mg tablets
95662998 Oxprenolol 160mg modified-release tablets
95673990 Lisinopril 20mg tablets
95674990 Lisinopril 10mg tablets
95675990 Lisinopril 5mg tablets
95678990 Lisinopril 20mg tablets
95680990 Lisinopril 5mg tablets
95682990 Lisinopril 20mg tablets
95684990 Lisinopril 5mg tablets
95685990 Lisinopril 2.5mg tablets
95688990 Lisinopril 20mg tablets
95689990 Lisinopril 10mg tablets
95690990 Lisinopril 5mg tablets
95691990 Lisinopril 2.5mg tablets

95699990 Lisinopril 20mg tablets
95700990 Lisinopril 10mg tablets
95701990 Lisinopril 5mg tablets
95702990 Lisinopril 2.5mg tablets
95712990 Lisinopril 20mg tablets
95714990 DILTIAZEM HCL 300mg m/r caps
95715990 DILTIAZEM HCL 240mg m/r caps
95716990 DILTIAZEM HCL 180mg m/r caps
95717990 Lisinopril 5mg tablets
95723990 Lisinopril 5mg tablets
95723998 Nifedipine 12 20mg Modified-release tablet
95724990 Lisinopril 2.5mg tablets
95724996 Nifedipine 20mg Capsule
95724997 Nifedipine 5mg capsules
95724998 Nifedipine 10mg capsules
95730997 Nicardipine 30mg capsules
95730998 Nicardipine 20mg capsules
95765997 Nadolol 80mg / Bendroflumethiazide 5mg tablets
95765998 Nadolol 40mg / Bendroflumethiazide 5mg tablets
95766997 Nadolol 80mg tablets
95766998 Nadolol 40mg tablets
95787990 Lisinopril 20mg tablets
95788990 Lisinopril 10mg tablets
95789990 Lisinopril 5mg tablets
95790990 Lisinopril 2.5mg tablets
95799996 MINOXIDIL 10mg tablets
95799997 MINOXIDIL 5mg tablets
95799998 MINOXIDIL 2.5mg tablets
95800996 Minoxidil 10mg tablets
95800997 Minoxidil 5mg tablets
95800998 Minoxidil 2.5mg tablets
95825998 Metoprolol 200mg modified-release tablets
95826997 Metoprolol 100mg tablets
95826998 Metoprolol 50mg tablets
95827998 Metolazone 5mg tablets
95836997 NIFEDIPINE 60mg m/r tablets
95836998 NIFEDIPINE 30mg m/r tablets
95852990 Nicardipine 30mg capsules
95853998 METHYLDO+HYDROCL 250/15mg tab
95855998 Methyldopa with hydrochlorothiazide Tablet
95856997 Methyldopa 50mg/ml Injection
95856998 Methyldopa 250mg/5ml oral suspension
95861998 METHYCLOTHIAZIDE 5mg tablets
95862998 Methyclothiazide 5mg Tablet
95870998 Methoserpidine with benzthiazide Tablet
95871997 Methoserpidine 10mg Tablet
95871998 Methoserpidine 5mg Tablet
95897998 Meprobamate with bendroflumethiazide Tablet
95908998 Mefruside 25mg Tablet
95956998 BETAXOLOL HCL 20mg tablets

95957998 Betaxolol 20mg tablets
95958997 BETHANIDINE 50mg tablets
95958998 BETANIDINE 10mg tablets
95959997 Bethanidine sulphate 50mg tablets
95959998 Bethanidine sulphate 10mg tablets
96002990 Bisoprolol 5mg tablets
96013990 Indapamide 2.5mg tablets
96017990 Bisoprolol 10mg tablets

96031998 Labetalol 50mg/10ml solution for injection pre-filled syringes
96032997 Labetalol 200mg tablets
96032998 Labetalol 100mg tablets
96043997 METOPROLOL 100mg tablets
96043998 METOPROLOL 50mg tablets
96065990 Celiprolol 200mg tablets
96070997 CO-TENIDONE 100/25mg tablets
96070998 CO-TENIDONE 50/12.5mg tablets
96093992 METOPROLOL 50mg tablets
96098992 DILTIAZEM HCl SR 300 MG CAP
96099992 CAPTOPRIL 4 MG/ML LIQ
96101990 Sotalol 40mg tablets
96129997 Indapamide 1.5mg modified-release tablets
96129998 Indapamide 2.5mg tablets
96164990 Verapamil 120mg tablets
96169990 Nifedipine 10mg modified-release tablets
96178990 Enalapril 20mg tablets
96180990 Enalapril 5mg tablets
96203998 Hydroflumethiazide 50mg Tablet
96209997 HYDROCHLOROTHIAZIDE 50mg tabs
96209998 HYDROCHLOROTHIAZIDE 25mg tabs
96212997 Hydrochlorothiazide 50mg tablets
96212998 Hydrochlorothiazide 25mg tablets

96214998 Hydralazine 20mg powder for solution for injection ampoules
96219990 Bendroflumethiazide 2.5mg tablets
96222998 Metolazone 500microgram low dose Tablet
96243990 Bisoprolol 5mg tablets
96276989 Bisoprolol 10mg tablets
96289990 Bisoprolol 5mg tablets
96313990 Bendroflumethiazide 2.5mg tablets
96332992 METOPROLOL FUMARATE 190 MG TAB
96337989 Bisoprolol 10mg tablets
96337990 Bisoprolol 5mg tablets
96346989 Bisoprolol 10mg tablets
96346990 Bisoprolol 5mg tablets
96348989 Bisoprolol 10mg tablets
96348990 Bisoprolol 5mg tablets
96349989 Bisoprolol 10mg tablets
96349990 Bisoprolol 5mg tablets
96365990 Nifedipine 5mg capsules

96368996 DILTIAZEM HCL 200mg m/r caps
96368997 DILTIAZEM HCL 300mg m/r caps
96368998 DILTIAZEM HCL 60mg m/r tablets
96380990 Bendroflumethiazide 2.5mg tablets
96406997 Diazoxide 300mg/20ml solution for injection ampoules
96456997 Debrisoquine 20mg tablets
96456998 Debrisoquine 10mg tablets
96465992 SOTALOL HCl 40 MG INJ
96473998 Furosemide with penbutolol Tablet
96587998 Cyclopenthiazide 500microgram tablets
96630998 Clopamide 5mg with Pindolol 10mg tablets

96631998 Clonidine 150micrograms/1ml solution for injection ampoules
96632997 Clonidine 300microgram tablets
96632998 Clonidine 100microgram tablets
96640989 Nifedipine 12 20mg Modified-release tablet
96640990 Nifedipine 10mg modified-release tablets
96657990 Enalapril 20mg tablets
96658990 Enalapril 2.5mg tablets
96661988 Enalapril 10mg tablets
96664990 Enalapril 20mg tablets
96665988 Enalapril 10mg tablets
96665989 Enalapril 5mg tablets
96667990 Enalapril 20mg tablets
96668988 Enalapril 10mg tablets
96668989 Enalapril 5mg tablets
96678990 Enalapril 20mg tablets
96679988 Enalapril 10mg tablets
96679989 Enalapril 5mg tablets
96683997 Chlortalidone 25mg with Atenolol 100mg tablets
96683998 Chlortalidone 12.5mg with Atenolol 50mg tablets
96684990 Diltiazem 120mg modified-release tablets
96685997 Chlortalidone 100mg tablets
96685998 Chlortalidone 50mg tablets
96686990 Enalapril 20mg tablets
96687988 Enalapril 10mg tablets
96687989 Enalapril 5mg tablets
96687990 Enalapril 2.5mg tablets
96689990 Enalapril 20mg tablets
96690988 Enalapril 10mg tablets
96690989 Enalapril 5mg tablets
96690990 Enalapril 2.5mg tablets
96696998 Chlorothiazide 500mg tablets
96710992 METOPROLOL FUMARATE 95 MG TAB
96711989 Celiprolol 400mg tablets
96714990 Sotalol 80mg tablets
96719989 Celiprolol 400mg tablets
96735989 Celiprolol 400mg tablets
96735990 Celiprolol 200mg tablets
96782992 TIMOLOL 10MG/BENDROFLUAZIDE 2.5MG TAB

96795996 Enalapril 2.5mg wafer
96795997 Enalapril titration pack
96795998 Enalapril 20mg tablets
96796992 PERINDOPRIL/TERT-BUTYLAMINE 2 MG TAB
96796996 Enalapril 10mg tablets
96796997 Enalapril 5mg tablets
96796998 Enalapril 2.5mg tablets
96822990 Diltiazem 60mg modified-release tablets
96828992 METOPROLOL 100mg tablets
96832988 Verapamil 120mg tablets
96832989 Verapamil 80mg tablets
96832990 Verapamil 40mg tablets
96849989 Metoprolol 100mg tablets
96849990 Metoprolol 50mg tablets
96856990 Labetalol 100mg tablets
96857992 DILTIAZEM HCL 300mg m/r caps
96862990 Indapamide 2.5mg tablets
96864990 Pindolol 5mg tablets
96870990 Diltiazem 60mg modified-release tablets
96876989 Nifedipine 10mg modified-release tablets
96876990 Nifedipine 30mg modified-release tablets
96882992 VERAPAMIL S/F 40 MG/5ML SOL
96888996 Captopril 50mg tablets
96888997 Captopril 25mg tablets
96888998 Captopril 12.5mg tablets
96895989 Co-tenidone 100mg/25mg tablets
96895990 Co-tenidone 50mg/12.5mg tablets
96900990 Enalapril 20mg tablets
96902988 Enalapril 10mg tablets
96902989 Enalapril 5mg tablets
96902990 Enalapril 2.5mg tablets
96904988 Diltiazem 90mg modified-release tablets
96904989 Diltiazem 90mg modified-release capsules
96904990 Diltiazem 12hr 180mg modified-release capsules
96907998 Atenolol 5mg/10ml solution for injection ampoules
96908997 DILTIAZEM HCL 120mg m/r tabs
96908998 DILTIAZEM HCL 90mg m/r tablets
96924988 Atenolol 100mg tablets
96924989 Atenolol 50mg tablets
96924990 Atenolol 25mg tablets
96927992 DILTIAZEM HCL 180mg m/r caps
96940997 Atenolol 50mg tablets
96940998 Atenolol 100mg tablets

96990998 Acebutolol 200mg / Hydrochlorothiazide 12.5mg tablets
96992996 Acebutolol 400mg tablets
96992997 Acebutolol 200mg capsules
96992998 Acebutolol 100mg capsules
96993988 Nifedipine 10mg modified-release tablets
97039990 Captopril 50mg tablets

97040990 Captopril 25mg tablets
97048992 NIFEDIPINE 10mg capsules
97060998 CO-ZIDOCAPT 50mg/25mg tablets
97086992 CAPTOPRIL 100 MG TAB
97098998 OXPRENOLOL 160mg m/r tablets
97116989 Verapamil 240mg modified-release tablets
97122990 Diltiazem 60mg modified-release tablets
97124998 Oxprenolol 160mg Tablet
97125996 Oxprenolol 80mg tablets
97125997 Oxprenolol 40mg tablets
97125998 Oxprenolol 20mg tablets
97136989 Sotalol 80mg tablets
97136990 Sotalol 40mg tablets
97137989 Atenolol 50mg tablets
97137990 Atenolol 25mg tablets
97139989 Captopril 25mg tablets
97139990 Captopril 12.5mg tablets
97157990 Sotalol 80mg tablets
97159990 Nifedipine 12 20mg Modified-release tablet
97204996 Methyldopa 500mg tablets
97204997 Methyldopa 250mg tablets
97204998 Methyldopa 125mg tablets
97214997 Hydralazine 50mg tablets
97214998 Hydralazine 25mg tablets
97217997 Bendroflumethiazide 5mg tablets
97217998 Bendroflumethiazide 2.5mg tablets
97222989 Sotalol 160mg tablets
97222990 Sotalol 80mg tablets
97241998 VERAPAMIL 240mg m/r tablets
97251998 Telmisartan 80mg / Hydrochlorothiazide 12.5mg tablets
97272997 NICARDIPINE 30mg capsules
97272998 NICARDIPINE 20mg capsules
97295998 INDAPAMIDE 2.5mg tablets
97337998 CLONIDINE 150mcg/mL injection
97347992 ENALAPRIL MALEATE 40 MG TAB
97350996 LABETALOL 400mg tablets
97350997 LABETALOL 200mg tablets
97350998 LABETALOL 100mg tablets
97359997 ENALAPRIL MAL tabs titre pack
97359998 ENALAPRIL MALEATE 20mg tablets
97382996 VERAPAMIL 120mg tablets
97382997 VERAPAMIL 80mg tablets
97382998 VERAPAMIL 40mg tablets
97425998 HYDROFLUMETHIAZIDE 50mg tabs
97491998 ENALAPRIL MAL 20mg tabs x28
97510998 NIFEDIPINE 20mg m/r tablets
97551998 Reserpine with hydrochlorothiazide tablet
97552998 RESERPINE 250mcg tablets
97556990 Bendroflumethiazide 5mg tablets
97557990 Bendroflumethiazide 2.5mg tablets

97558990 Atenolol 25mg tablets
97588996 OXPRENOLOL 80mg tablets
97597998 Spironolactone with propranolol tablet
97635998 NIFEDIPINE 20mg m/r tablets
97649998 OLMESARTAN MEDOXOMIL 40mg tabs
97680992 METOPROLOL 200mg m/r tablets
97707996 Diltiazem 12hr 180mg modified-release capsules
97707997 Diltiazem 12hr 120mg modified-release capsules
97707998 Diltiazem 90mg modified-release capsules
97710997 CO-TENIDONE 100/25mg tablets
97710998 CO-TENIDONE 50/12.5mg tablets

97727997 Esmolol HCl 250mg/ml concentrate solution for infusion
97733989 Nifedipine 10mg capsules
97733990 Nifedipine 5mg capsules
97733996 DILTIAZEM HCL 300mg m/r caps
97733997 DILTIAZEM HCL 180mg m/r caps
97733998 DILTIAZEM HCL 120mg m/r caps
97740989 Metoprolol 100mg tablets
97740990 Metoprolol 50mg tablets
97741992 METHYLDOPA 250 MG CAP
97745989 Labetalol 200mg tablets
97745990 Labetalol 100mg tablets
97747990 Indapamide 2.5mg tablets
97764988 Diltiazem 120mg modified-release tablets
97764989 Diltiazem 90mg modified-release tablets
97764990 Diltiazem 60mg modified-release tablets
97769989 Co-tenidone 100mg/25mg tablets
97769990 Co-tenidone 50mg/12.5mg tablets
97786988 Atenolol 100mg tablets
97786989 Atenolol 50mg tablets
97786990 Atenolol 25mg tablets
97790998 VERAPAMIL 160mg tablets
97793988 Captopril 50mg tablets
97793989 Captopril 25mg tablets
97793990 Captopril 12.5mg tablets
97801989 Metoprolol 100mg tablets
97801990 Metoprolol 50mg tablets
97804990 Indapamide 2.5mg tablets
97816998 ESMOLOL HCL 100mg/10mL inj
97820989 Captopril 25mg tablets
97820990 Captopril 12.5mg tablets
97821989 Captopril 25mg tablets
97821998 VERAPAMIL 120mg m/r tablets
97822998 Verapamil hcl 120mg modified release tablets
97831989 Captopril 25mg tablets
97831990 Captopril 12.5mg tablets
97832988 Captopril 50mg tablets
97832989 Captopril 25mg tablets
97832990 Captopril 12.5mg tablets

97833988 Captopril 50mg tablets
97833989 Captopril 25mg tablets
97833990 Captopril 12.5mg tablets
97856990 Sotalol 80mg tablets
97895989 Verapamil 240mg modified-release tablets
97896989 Verapamil 80mg tablets
97939989 Verapamil 80mg tablets
97939990 Verapamil 40mg tablets
97949998 ATENOL+CO-AMILOZ 50/2.5/25mg
97955979 FELODIPINE 2.5mg m/r tablets
97958979 FELODIPINE 2.5mg m/r tablets
97968988 Atenolol 100mg tablets
97968989 Atenolol 50mg tablets
97968990 Atenolol 25mg tablets
97981979 Amlodipine 10mg tablets
98003979 Amlodipine 5mg tablets
98027979 NIFEDIPINE 20mg m/r tablets
98033979 Nifedipine 20mg modified-release tablets
98045979 NIFEDIPINE 60mg m/r tablets
98046979 NIFEDIPINE 30mg m/r tablets
98047979 NIFEDIPINE 30mg m/r tablets
98049979 NIFEDIPINE 30mg m/r tablets
98052979 Nifedipine 10mg modified-release tablets
98058990 Enalapril 5mg tablets
98093979 DILTIAZEM HCL 60mg m/r tablets
98097979 DILTIAZEM HCL 200mg m/r caps
98106979 Diltiazem 60mg modified-release capsules
98113979 Diltiazem 120mg modified-release capsules
98115979 Diltiazem 120mg modified-release capsules
98117979 Diltiazem 90mg modified-release capsules
98118979 Diltiazem 90mg modified-release capsules
98128979 Diltiazem 90mg modified-release tablets
98143989 Atenolol 100mg tablets
98143990 Atenolol 50mg tablets
98144989 Bendroflumethiazide 2.5mg tablets
98144990 Bendroflumethiazide 5mg tablets
98153998 RAMIPRIL 10mg capsules
98169997 CILAZAPRIL 5mg tablets
98169998 CILAZAPRIL 2.5mg tablets
98181989 Co-tenidone 50mg/12.5mg tablets
98181990 Co-tenidone 100mg/25mg tablets
98216996 ENALAPRIL MAL 10mg tabs x28
98216997 ENALAPRIL MALEATE 5mg tablets
98216998 ENALAPRIL MALEATE 2.5mg tabs
98231992 NIFEDIPINE 10mg capsules
98267998 NIFEDIPINE 20mg m/r tablets
98276998 CLONIDINE 250mcg m/r capsules
98284998 OXPRENOLOL 160mg tablets
98285996 OXPRENOLOL 80mg tablets
98285997 OXPRENOLOL 40mg tablets

98285998 OXPRENOLOL 20mg tablets
98286996 LABETALOL 200mg tablets
98286997 LABETALOL 100mg tablets
98286998 LABETALOL 50mg tablets
98290997 NICARDIPINE 45mg m/r capsules
98290998 NICARDIPINE 30mg m/r capsules
98292996 METHYLDOPA 500mg tablets
98292997 METHYLDOPA 250mg tablets
98292998 METHYLDOPA 125mg tablets
98297996 SOTALOL 200mg tablets
98297997 SOTALOL 80mg tablets
98297998 SOTALOL 40mg tablets
98300998 ATENOLOL 50mg tablets
98301998 CO-TENIDONE 100/25mg tablets
98322979 Losartan 50mg / Hydrochlorothiazide 12.5mg tablets
98336989 Bendroflumethiazide 2.5mg tablets
98336990 Bendroflumethiazide 5mg tablets
98338997 NIFEDIPINE 60mg m/r tablets
98338998 NIFEDIPINE 30mg m/r tablets
98344989 Co-tenidone 100mg/25mg tablets
98344990 Co-tenidone 50mg/12.5mg tablets
98348990 Diltiazem 60mg modified-release tablets
98361988 Labetalol 400mg tablets
98366988 Verapamil 120mg tablets
98366989 Verapamil 80mg tablets
98366990 Verapamil 40mg tablets
98367989 Nifedipine 10mg capsules
98367990 Nifedipine 5mg capsules
98367998 NADOLOL+BENDROFLU 80/5mg tabs
98368997 NADOLOL 80mg tablets
98368998 NADOLOL 40mg tablets
98398998 TIMOLOL+CO-AMILOZ 10/2.5/25mg
98404998 TIMOLOL 10mg tablets
98405998 METHYLDOPAT HCL 250mg/5mL inj
98406998 METHYLDOPA 250mg/5mL mixture
98422979 Ramipril 2.5mg capsules
98454998 Reserpine with phenobarbital & theobromine tablet
98471997 Cilazapril 5mg tablets
98471998 Cilazapril 2.5mg tablets
98473989 Nifedipine 5mg capsules
98482989 Metoprolol 100mg tablets
98482990 Metoprolol 50mg tablets
98488990 Methyldopa 250mg tablets
98488998 Methoserpidine with benzthiazide Tablet
98496979 LISINO+HYDROCHL 10/12.5mg tabs
98499979 LISINO+HYDROCHL 10/12.5mg tabs
98553979 LISINOPRIL 2.5mg tablets
98562979 Lisinopril 2.5mg tablets
98567988 Diltiazem 120mg modified-release tablets
98567989 Diltiazem 90mg modified-release tablets

98567990 Diltiazem 60mg modified-release tablets
98581979 Enalapril 10mg tablets
98591988 Atenolol 25mg tablets
98591989 Atenolol 50mg tablets
98591990 Atenolol 100mg tablets
98600998 METOPROL+HYDROCH 200/25mg m/r
98603998 METOPROLOL 200mg m/r tablets
98604998 METOPROLOL 5mg/5mL injection
98624979 CAPTOPRIL 12.5mg tablets
98624998 VERAPAMIL 160mg tablets
98625996 VERPAMIL HCL 120mg tabs x56
98625997 VERAPAMIL 80mg tablets
98625998 VERAPAMIL 40mg tablets
98636989 Co-tenidone 100mg/25mg tablets
98636990 Co-tenidone 50mg/12.5mg tablets
98650990 Metoprolol 50mg tablets
98734979 METHYLDOPA 500mg tablets
98755998 VERAPAMIL 160mg tablets
98756998 VERPAMIL HCL 120mg tabs x56
98775998 PENBUTOL+FRUSEM 40/20mg tabs
98797990 Bendroflumethiazide 2.5mg tablets
98798988 Atenolol 25mg tablets
98798989 Atenolol 100mg tablets
98798990 Atenolol 50mg tablets
98799990 Atenolol 50mg tablets
98816998 TIMOLOL+BENDROFL 10/2.5mg tabs
98844979 CELIPROLOL 200mg tablets
98846979 CELIPROLOL 200mg tablets
98851996 ACEBUTOLOL 400mg tablets
98851997 ACEBUTOLOL 200mg capsules
98851998 ACEBUTOLOL 100mg capsules
98876979 BISOPROLOL FUMARATE 2.5mg tabs
98877996 CAPTOPRIL 50mg tablets
98877997 CAPTOPRIL 25mg tablets
98877998 CAPTOPRIL 12.5mg tablets
98886997 NIFEDIPINE 20mg m/r tablets
98886998 NIFEDIPINE 10mg m/r tablets
98888997 NIFEDIPINE 10mg capsules
98888998 NIFEDIPINE 5mg capsules
98893979 Bisoprolol 1.25mg tablets
98894998 LABETALOL 400mg tablets
98937998 Metoprolol 5mg/5ml solution for injection ampoules
98943997 BENDROFLUMETHIAZIDE 5mg tablet
98943998 BENDROFLUMETHIAZIDE 2.5mg tabs
98961997 PINDOLOL 15mg tablets
98961998 PINDOLOL 5mg tablets
98963998 PINDOLOL+CLOPAMIDE 10/5mg tabs
98974979 NADOLOL 80mg tablets
98985996 Felodipine 2.5mg modified-release tablets
98985997 Felodipine 10mg modified-release tablets

98985998 Felodipine 5mg modified-release tablets
99013997 Nicardipine 45mg modified-release capsules
99013998 Nicardipine 30mg modified-release capsules
99017979 Atenolol 50mg tablets
99031998 CO-PRENOZIDE 160/0.25mg tabs
99033998 LABETALOL 100mg/20mL injection
99040989 Verapamil 240mg modified-release tablets
99067996 ATENOLOL 5mg/10mL injection
99067997 ATENOLOL 25mg/5mL syrup
99067998 ATENOLOL 100mg tablets
99068998 CO-TENIDONE 50/12.5mg tablets
99069998 Meprobamate with bendroflumethiazide Tablet
99102997 METHYLDOPA 500mg tablets
99102998 METHYLDOPA 250mg tablets
99118998 SOTAL+HYDROCHLOR 160/25mg tab
99119997 SOTALOL 160mg tablets
99119998 SOTALOL 80mg tablets
99132998 OXPRENOLOL 160mg m/r tablets
99151998 ACEBU+HYDROCH 200/12.5mg tabs
99161998 CHLOROTHIAZIDE 500mg tablets
99162997 LISINO+HYDROCHL 10/12.5mg tabs
99162998 LISINO+HYDROCHL 20/12.5mg tabs
99169988 Atenolol 25mg tablets
99169989 Atenolol 100mg tablets
99169990 Atenolol 50mg tablets
99192998 Rauwolfia alkaloids 2mg tablet
99266998 Enalapril 20mg / Hydrochlorothiazide 12.5mg tablets
99272998 ENALAP+HYDROCHL 20/12.5mg tabs
99277990 Atenolol 50mg tablets
99278988 Atenolol 25mg tablets
99278989 Atenolol 100mg tablets
99278990 Atenolol 50mg tablets
99328997 Lisinopril 10mg / Hydrochlorothiazide 12.5mg tablets
99328998 Lisinopril 20mg / Hydrochlorothiazide 12.5mg tablets
99334988 Verapamil 120mg tablets
99334989 Verapamil 80mg tablets
99334990 Verapamil 40mg tablets
99336989 Verapamil 80mg tablets
99366998 POLYTHIAZIDE 1mg tablets
99368998 BENDROFLUMETHIAZIDE 5mg tablet
99373998 CYCLOPENTHIAZIDE 500mcg tabs
99384996 CILAZAPRIL 1mg tablets
99384997 CILAZAPRIL 500mcg tablets
99384998 CILAZAPRIL 250mcg tablets
99407998 NIFEDIPINE 20mg m/r tablets
99429998 METOLAZONE 5mg tablets
99458998 DILTIAZEM HCL 60mg m/r tablets
99459998 METOPROL+CHLORTHAL 100/12.5mg
99460997 METOPROLOL 100mg tablets
99460998 METOPROLOL 50mg tablets

99461998 METOPROLOL 200mg m/r tablets
99501989 Metoprolol 100mg tablets
99501990 Metoprolol 50mg tablets
99508989 Methyldopa 250mg tablets
99509989 Methyldopa 250mg tablets
99526996 GUANETHIDINE 10mg/1mL inj
99526997 GUANETHIDINE 25mg tablets
99526998 GUANETHIDINE 10mg tablets
99538988 Labetalol 400mg tablets
99538989 Labetalol 200mg tablets
99538990 Labetalol 100mg tablets
99539989 Labetalol 200mg tablets
99539990 Labetalol 100mg tablets
99548997 CHLORTHALIDONE 100mg tablets
99548998 CHLORTALIDONE 50mg tablets
99568990 Hydralazine 25mg tablets
99569989 Hydralazine 50mg tablets
99569990 Hydralazine 25mg tablets
99620988 Diltiazem 60mg modified-release capsules
99620989 Diltiazem 12hr 120mg modified-release capsules
99620990 Diltiazem 60mg modified-release tablets
99622990 Diltiazem 60mg modified-release tablets
99660996 Cilazapril 1mg tablets
99660997 Cilazapril 500microgram tablets
99660998 Cilazapril 250micrograms tablets
99677997 HYDROCHLOROTHIAZIDE 50mg tabs
99677998 HYDROCHLOROTHIAZIDE 25mg tabs
99678997 BETHANIDINE 50mg tablets
99678998 BETANIDINE SULFATE 10mg tabs
99686998 TELMIS+HYDROCHL 80/12.5mg tabs
99707989 Oxprenolol 160mg modified-release tablets
99712989 Oxprenolol 40mg tablets
99721988 Nifedipine 20mg modified-release tablets
99721989 Nifedipine 10mg capsules
99721990 Nifedipine 5mg capsules
99723990 Nifedipine 5mg capsules
99724989 Nifedipine 10mg capsules
99734998 XIPAMIDE 20mg tablets
99782997 DEBRISOQUINE 20mg tablets
99782998 DEBRISOQUINE 10mg tablets
99783997 Methoserpidine 10mg Tablet
99783998 Methoserpidine 5mg Tablet
99790989 Bendroflumethiazide 5mg tablets
99790990 Bendroflumethiazide 2.5mg tablets
99791990 Bendroflumethiazide 2.5mg tablets
99792989 Bendroflumethiazide 5mg tablets
99792990 Bendroflumethiazide 2.5mg tablets
99808998 NADOLOL+BENDROFLU 40/5mg tabs
99809998 VERAPAMIL 5mg/2mL injection
99810997 VERAPAMIL 80mg tablets

99810998 VERAPAMIL 40mg tablets
99819998 METOPROLOL+HYDROCHL 100/12.5m
99850997 CLONIDINE 300mcg tablets
99850998 CLONIDINE 100microgram tablets
99851996 CAPTOPRIL 50mg tablets
99851997 CAPTOPRIL 25mg tablets
99851998 CAPTOPRIL 12.5mg tablets
99891998 TIMOLOL 10mg tablets
99892997 METOPROLOL 100mg tablets
99892998 METOPROLOL 50mg tablets
99894997 BENDROFLUMETHIAZIDE 5mg tablet
99894998 BENDROFLUMETHIAZIDE 2.5mg tabs
99916998 MEFRUSIDE 25mg tablets
99943997 BENDROFLUMETHIAZIDE 5mg tablet
99943998 BENDROFLUMETHIAZIDE 2.5mg tabs
99944996 HYDRALAZINE HCL 20mg inj
99944997 HYDRALAZINE HCL 50mg tabs
99944998 HYDRALAZINE HCL 25mg tabs
99975996 METHYLDOPA 500mg tablets
99975997 METHYLDOPA 250mg tablets
99975998 METHYLDOPA 125mg tablets
99996998 Reserpine with bendroflumethiazide tablet

Immunosupressants

drugcode	genericname
53189979	Ciclosporin 50mg capsules
53190979	Ciclosporin 100mg capsules
53191979	Ciclosporin 25mg capsules
55575979	Ciclosporin 50mg capsules
55576979	Ciclosporin 100mg capsules
55577979	Ciclosporin 25mg capsules
60124979	Ciclosporin 50mg capsules
64115979	Methotrexate 25mg/3ml solution for injection pre-filled syringes
64937979	Tacrolimus 500micrograms/5ml oral suspension
69316979	Azathioprine 125mg/5ml oral suspension
73065978	Methotrexate 7.5mg/0.15ml solution for injection pre-filled disposable devices
73066978	Methotrexate 7.5mg/0.15ml solution for injection pre-filled disposable devices
73067978	Methotrexate 30mg/0.6ml solution for injection pre-filled disposable devices
73068978	Methotrexate 30mg/0.6ml solution for injection pre-filled disposable devices
73069978	Methotrexate 27.5mg/0.55ml solution for injection pre-filled disposable devices
73071978	Methotrexate 25mg/0.5ml solution for injection pre-filled disposable devices
73072978	Methotrexate 25mg/0.5ml solution for injection pre-filled disposable devices
73073978	Methotrexate 22.5mg/0.45ml solution for injection pre-filled disposable devices
73074978	Methotrexate 22.5mg/0.45ml solution for injection pre-filled disposable devices
73075978	Methotrexate 20mg/0.4ml solution for injection pre-filled disposable devices
73076978	Methotrexate 20mg/0.4ml solution for injection pre-filled disposable devices
73077978	Methotrexate 17.5mg/0.35ml solution for injection pre-filled disposable devices
73078978	Methotrexate 17.5mg/0.35ml solution for injection pre-filled disposable devices
73079978	Methotrexate 15mg/0.3ml solution for injection pre-filled disposable devices
73080978	Methotrexate 15mg/0.3ml solution for injection pre-filled disposable devices
73081978	Methotrexate 12.5mg/0.25ml solution for injection pre-filled disposable devices
73082978	Methotrexate 12.5mg/0.25ml solution for injection pre-filled disposable devices
73083978	Methotrexate 10mg/0.2ml solution for injection pre-filled disposable devices
73084978	Methotrexate 10mg/0.2ml solution for injection pre-filled disposable devices
75901978	Leflunomide 15mg tablets
78442979	Methotrexate 50mg/2ml solution for injection vials
79182979	Tacrolimus 2.5mg/5ml oral solution
80925979	Azathioprine 25mg/5ml oral suspension
80929979	Azathioprine 20mg/5ml oral suspension
80961979	Azathioprine 10mg/5ml oral suspension
81029998	Tacrolimus 5mg capsules
81030998	Tacrolimus 1mg capsules
81031998	Tacrolimus 500microgram capsules
81076998	Tacrolimus 5mg/5ml oral suspension
81152998	Tacrolimus 1mg capsules
81153998	Tacrolimus 500microgram capsules
81282998	Ciclosporin 100mg capsules
81283998	Ciclosporin 50mg capsules
81284998	Ciclosporin 25mg capsules
81490998	Methotrexate 27.5mg/0.55ml prefilled syringes
81491998	Methotrexate 22.5mg/0.45ml prefilled syringes
81492998	Methotrexate 17.5mg/0.35ml prefilled syringes
81493998	Methotrexate 12.5mg/0.25ml prefilled syringes

81494998 Methotrexate 27.5mg/0.55ml solution for injection pre-filled disposable devices
81495998 Methotrexate 22.5mg/0.45ml solution for injection pre-filled disposable devices
81496998 Methotrexate 17.5mg/0.35ml solution for injection pre-filled disposable devices
81498998 Methotrexate 12.5mg/0.25ml solution for injection pre-filled disposable devices
81539998 Tacrolimus 1mg capsules
81540998 Tacrolimus 500microgram capsules
81638998 Methotrexate 30mg/1.5ml solution for injection pre-filled syringes
81640998 Methotrexate 25mg/1.25ml solution for injection pre-filled syringes
81642998 Methotrexate 20mg/1ml solution for injection pre-filled syringes
81771998 Ciclosporin 25mg capsules
81773998 Ciclosporin 100mg capsules
81774998 Ciclosporin 50mg capsules
81815998 Methotrexate 30mg/0.6ml prefilled syringes
81816998 Methotrexate 30mg/0.6ml solution for injection pre-filled syringes
81893998 Tacrolimus 2.5mg/5ml oral suspension
81896998 Sirolimus 500microgram tablets
81897998 Sirolimus 500microgram tablets
81988998 Tacrolimus 500microgram capsules
81989998 Tacrolimus 5mg capsules
81990998 Tacrolimus 1mg capsules
82203998 Azathioprine 50mg tablets
82204998 Azathioprine 25mg tablets
82312998 Tacrolimus 1mg granules sachets sugar free
82313998 Tacrolimus 200microgram granules sachets sugar free
82317998 Tacrolimus 1mg granules sachets sugar free
82318998 Tacrolimus 200microgram granules sachets sugar free
82480998 Ciclosporin 100mg capsules
82481998 Ciclosporin 50mg capsules
82482998 Ciclosporin 25mg capsules
82766998 Tacrolimus 3mg modified-release capsules
82767998 Tacrolimus 3mg modified-release capsules
82840998 Methotrexate 25mg/0.5ml prefilled syringes
82841998 Methotrexate 20mg/0.4ml prefilled syringes
82842998 Methotrexate 15mg/0.3ml prefilled syringes
82843998 Methotrexate 10mg/0.2ml prefilled syringes
82844998 Methotrexate 7.5mg/0.15ml prefilled syringes
82845998 Methotrexate 25mg/0.5ml solution for injection pre-filled syringes
82846998 Methotrexate 20mg/0.4ml solution for injection pre-filled syringes
82847998 Methotrexate 15mg/0.3ml solution for injection pre-filled syringes
82848998 Methotrexate 10mg/0.2ml solution for injection pre-filled syringes
82849998 Methotrexate 7.5mg/0.15ml solution for injection pre-filled syringes
83228978 Ciclosporin 50mg capsules
83229978 Ciclosporin 100mg capsules
83485998 Azathioprine 10mg capsules
84438998 Methotrexate 10mg/5ml oral solution
84439998 Methotrexate 10mg/5ml oral suspension
84516998 Tacrolimus 5mg modified-release capsules
84517998 Tacrolimus 1mg modified-release capsules
84518998 Tacrolimus 500microgram modified-release capsules
84519998 Tacrolimus 5mg modified-release capsules

84520998 Tacrolimus 1mg modified-release capsules
84521998 Tacrolimus 500microgram modified-release capsules
84920998 Ciclosporin 250mg/5ml solution for infusion ampoules
84921998 Ciclosporin 50mg/1ml solution for infusion ampoules
84927998 Ciclosporin 250mg/5ml solution for infusion ampoules
84928998 Ciclosporin 50mg/1ml solution for infusion ampoules
85097998 Methotrexate 1g/40ml solution for injection vials
85100998 Methotrexate 7.5mg/5ml oral suspension
85252998 Azathioprine 50mg/5ml oral suspension
85501998 Tacrolimus oral liquid
85639998 Methotrexate 7.5mg/0.75ml prefilled syringes
85640998 Methotrexate 10mg/ml prefilled syringes
85641998 Methotrexate 15mg/1.5ml prefilled syringes
85642998 Methotrexate 20mg/2ml prefilled syringes
85643998 Methotrexate 25mg/2.5ml prefilled syringes
85644998 Methotrexate 7.5mg/0.75ml solution for injection pre-filled syringes
85645998 Methotrexate 10mg/1ml solution for injection pre-filled syringes
85646998 Methotrexate 15mg/1.5ml solution for injection pre-filled syringes
85648998 Methotrexate 20mg/2ml solution for injection pre-filled syringes
85650998 Methotrexate 25mg/2.5ml solution for injection pre-filled syringes
85737998 Methotrexate 5g/50ml solution for infusion vials
85738998 Methotrexate 1g/10ml solution for injection vials
85776998 Methotrexate oral solution
85777998 Methotrexate 12.5mg/5ml oral suspension
86327998 Methotrexate 2.5mg/5ml oral suspension
86339998 Methotrexate 5mg/0.2ml solution for injection pre-filled syringes
86342998 Methotrexate 22.5mg/0.9ml solution for injection pre-filled syringes
86343998 Methotrexate 15mg/0.6ml solution for injection pre-filled syringes
86344998 Methotrexate 12.5mg/0.5ml solution for injection pre-filled syringes
86345998 Methotrexate 10mg/0.4ml solution for injection pre-filled syringes
86427998 Methotrexate 17.5mg/0.7ml solution for injection pre-filled syringes
86434998 Methotrexate 20mg/0.8ml solution for injection pre-filled syringes
86435998 Methotrexate 25mg/1ml solution for injection pre-filled syringes
86436998 Methotrexate 500mg/20ml solution for injection vials
86437998 Methotrexate 50mg/2ml solution for injection vials
86438998 Methotrexate 7.5mg/0.3ml solution for injection pre-filled syringes
86439998 Methotrexate 30mg/1.2ml solution for injection pre-filled syringes
86440998 Methotrexate 27.5mg/1.1ml solution for injection pre-filled syringes
86470998 Azathioprine 250mg/5ml oral solution
86519998 Azathioprine 50mg/5ml oral solution
86729998 Rituximab 500mg/50ml solution for infusion vials
86731998 Rituximab 100mg/10ml solution for infusion vials
86880998 Alemtuzumab 30mg/1ml solution for infusion vials
87029998 Tacrolimus 1mg/ml suspension
87974998 Tacrolimus 0.1% ointment
90439998 Sirolimus 2mg tablets
90488998 Sirolimus 2mg tablets
90824998 Tacrolimus 0.1% ointment
90825998 Tacrolimus 0.03% ointment
91215998 Azathioprine 50mg tablets

91287998 Basiliximab 20mg powder and solvent for solution for injection vials
91309998 Azathioprine capsules
91373997 Ciclosporin 10mg capsules
91373998 Ciclosporin 100mg/ml oral solution sugar free
91437998 Tacrolimus 0.03% ointment
91624998 Basiliximab 20mg powder and solvent for solution for injection vials
91672998 Sirolimus 1mg tablets
91673998 Sirolimus 1mg tablets
91710998 Sirolimus 1mg/ml oral solution sugar free
91711998 Sirolimus 1mg/ml oral solution sugar free
91939998 Tacrolimus 500microgram capsules
92244990 Ciclosporin 100mg capsules
92245990 Ciclosporin 50mg capsules
92246990 Ciclosporin 25mg capsules
92486996 Leflunomide 100mg tablets
92486997 Leflunomide 20mg tablets
92486998 Leflunomide 10mg tablets
92487996 Leflunomide 100mg tablets
92487997 Leflunomide 20mg tablets
92487998 Leflunomide 10mg tablets
92488997 Methotrexate 2.5mg tablets
92488998 Methotrexate 200mg/8ml solution for injection vials
92522979 Tacrolimus 500microgram capsules
92523979 Tacrolimus 500microgram capsules
92532979 Tacrolimus 1mg capsules
92533979 Tacrolimus 1mg capsules
92537979 Tacrolimus 1mg capsules
92544979 Ciclosporin 50mg capsules
92547998 Tacrolimus 5mg/1ml solution for infusion ampoules
92552979 Ciclosporin 100mg capsules
92555979 Ciclosporin 100mg capsules
92558979 Ciclosporin 25mg capsules
92559979 Ciclosporin 25mg capsules
92562996 Tacrolimus 500microgram capsules
92562997 Tacrolimus 5mg capsules
92562998 Tacrolimus 1mg capsules
92571979 Azathioprine 25mg tablets
92579979 Azathioprine 50mg tablets
92639979 Methotrexate 5g/200ml solution for infusion vials
92650979 Methotrexate 200mg/8ml solution for injection vials
92655979 Methotrexate 50mg/2ml solution for injection vials
92930998 Azathioprine 50mg tablets
92989996 Ciclosporin 100mg capsules
92989997 Ciclosporin 50mg capsules
92989998 Ciclosporin 25mg capsules
93074990 Methotrexate 2.5mg tablets
94078992 Azathioprine 100 mg tab
94308990 Azathioprine 50mg tablets
94417992 Azathioprine 50 mg sus
94593997 Ciclosporin 10mg capsules

94593998 Ciclosporin 50mg capsules
94600990 Methotrexate 20mg/0.8ml solution for injection pre-filled syringes
94690992 Azathioprine 125 mg tab
94691992 Azathioprine 10 mg tab
94697998 Azathioprine 50mg tablets
95153992 Azathioprine 10mg tablets
95252990 Azathioprine 50mg tablets
95725990 Azathioprine 50mg tablets
95866998 Methotrexate 25mg/ml injection
95867996 Methotrexate 5mg/2ml solution for injection vials
95867997 Methotrexate 10mg tablets
95867998 Methotrexate 2.5mg tablets
95868996 Methotrexate 50mg/3ml Injection
95868997 Methotrexate 5g/50ml solution for infusion vials
95868998 Methotrexate 50mg/2ml solution for injection vials
95869997 Methotrexate 10mg tablets
95869998 Methotrexate 2.5mg tablets
96199990 Azathioprine 50mg tablets
96279990 Methotrexate 2.5mg tablets
96580998 Ciclosporin 250mg/5ml solution for infusion ampoules
96581996 Ciclosporin 100mg capsules
96581997 Ciclosporin 25mg capsules
96581998 Ciclosporin 100mg/ml oral solution sugar free
96752989 Methotrexate 10mg tablets
96752990 Methotrexate 2.5mg tablets
96820988 Methotrexate 5mg/2ml solution for injection vials
96922989 Azathioprine 50mg tablets
96922990 Azathioprine 25mg tablets
96932998 Azathioprine 50mg powder for solution for injection vials
96933998 Azathioprine 50mg tablets
96934997 Azathioprine 50mg powder for solution for injection vials
96934998 Azathioprine 25mg tablets
97036997 Azathioprine 10mg tablets
97036998 Azathioprine 50mg tablets
97223992 Ciclosporin 250mg/5ml solution for infusion ampoules
97595996 Azathioprine 10mg tablets
97595997 Azathioprine 50mg tablets
97595998 Azathioprine 25mg tablets
97785990 Azathioprine 50mg tablets
98013988 Methotrexate 5g/50ml solution for infusion vials
98211990 Azathioprine 50mg tablets
98238996 Ciclosporin 25mg capsules
98238998 Ciclosporin 100mg/ml oral solution sugar free
98639990 Azathioprine 50mg tablets
98640990 Azathioprine 50mg tablets
98950997 Ciclosporin 50mg capsules
98950998 Ciclosporin 100mg capsules
98958988 Methotrexate 2.5mg tablets
98959989 Methotrexate 10mg tablets
98959990 Methotrexate 2.5mg tablets

99212997	Tacrolimus 5mg capsules
99212998	Tacrolimus 1mg capsules
99797989	Azathioprine 25mg tablets
99797990	Azathioprine 50mg tablets
99798990	Azathioprine 50mg tablets
99799990	Azathioprine 50mg tablets
99956998	Methotrexate 5mg/2ml solution for injection vials

Interactions

drugcode	genericname
52097979	Darunavir 800mg tablets
52098979	Darunavir 800mg tablets
53189979	Ciclosporin 50mg capsules
53190979	Ciclosporin 100mg capsules
53191979	Ciclosporin 25mg capsules
53323979	Darunavir 100mg/ml oral suspension sugar free
55575979	Ciclosporin 50mg capsules
55576979	Ciclosporin 100mg capsules
55577979	Ciclosporin 25mg capsules
60124979	Ciclosporin 50mg capsules
60538979	Etravirine 200mg tablets
73291978	Posaconazole 100mg gastro-resistant tablets
79758979	Ketoconazole 100mg/5ml oral suspension
80920998	Etravirine 200mg tablets
81049979	Amiodarone 50mg/5ml oral solution
81066979	Amiodarone 20mg/5ml oral suspension
81070979	Amiodarone 200mg/5ml oral suspension
81074979	Amiodarone 100mg/5ml oral suspension
81076979	Amiodarone 100mg/5ml oral solution
81282998	Ciclosporin 100mg capsules
81283998	Ciclosporin 50mg capsules
81284998	Ciclosporin 25mg capsules
81771998	Ciclosporin 25mg capsules
81773998	Ciclosporin 100mg capsules
81774998	Ciclosporin 50mg capsules
82132998	Ritonavir 100mg tablets
82133998	Ritonavir 100mg tablets
82466998	Tipranavir 100mg/ml oral solution sugar free
82480998	Ciclosporin 100mg capsules
82481998	Ciclosporin 50mg capsules
82482998	Ciclosporin 25mg capsules
82698998	Darunavir 150mg tablets
82700998	Darunavir 75mg tablets
82729998	Miconazole 25mg/ml oral gel
83038998	Darunavir 400mg tablets
83039998	Darunavir 400mg tablets
83041998	Darunavir 600mg tablets
83228978	Ciclosporin 50mg capsules
83229978	Ciclosporin 100mg capsules
83482998	Etravirine 100mg tablets
83483998	Etravirine 100mg tablets
83845998	Lopinavir 100mg / Ritonavir 25mg tablets
83851998	Atazanavir 300mg capsules
83852998	Atazanavir 300mg capsules
83988998	Miconazole 50mg buccal tablets
83989998	Miconazole 50mg muco-adhesive buccal tablets sugar free
84831998	Darunavir 300mg tablets
84920998	Ciclosporin 250mg/5ml solution for infusion ampoules

84921998 Ciclosporin 50mg/1ml solution for infusion ampoules
84927998 Ciclosporin 250mg/5ml solution for infusion ampoules
84928998 Ciclosporin 50mg/1ml solution for infusion ampoules
85000998 Ketoconazole oral solution
85026998 Amiodarone 25mg/5ml oral suspension
85651998 Amiodarone 150mg/3ml solution for injection ampoules
85841998 Lopinavir 200mg / Ritonavir 50mg tablets
85842998 Lopinavir 200mg / Ritonavir 50mg tablets
86212998 Posaconazole 40mg/ml oral suspension
86213998 Posaconazole 40mg/ml oral suspension
86483998 Tipranavir 250mg capsules
86776998 Saquinavir mesilate 500mg tablets
87326998 Fosamprenavir 700mg tablets
87562998 Atazanavir 150mg capsules
87564998 Atazanavir 200mg capsules
87565998 Atazanavir 150mg capsules
87566998 Atazanavir 100mg capsules
88043997 Amiodarone hydrochloride 200mg tablets
88043998 Amiodarone hydrochloride 100mg tablets
88259996 Efavirenz 200mg capsules
88259998 Efavirenz 50mg capsules
88260996 Efavirenz 200mg capsules
88260997 Efavirenz 100mg capsules
88260998 Efavirenz 50mg capsules
88917998 Miconazole 25mg/ml oral gel
89316997 Indinavir 400mg capsules
89322997 Indinavir 400mg capsules
89510998 Saquinavir mesilate 200mg capsules
89591998 Efavirenz 600mg tablets
89676997 Ritonavir 80mg/1ml oral solution sugar free
89676998 Ritonavir 100mg capsules
89677998 Ritonavir 100mg capsules
89802998 Itraconazole 50mg/5ml oral solution sugar free
89802998 Itraconazole 50mg/5ml oral solution sugar free
89804998 Itraconazole 50mg/5ml oral solution sugar free
89804998 Itraconazole 50mg/5ml oral solution sugar free
90389998 Itraconazole 100mg capsules
90389998 Itraconazole 100mg capsules
90651998 Efavirenz 600mg tablets
90656998 Amiodarone hydrochloride 100mg tablets
90845997 Lopinavir 400mg/5ml / Ritonavir 100mg/5ml oral solution
90845998 Lopinavir & ritonavir 133.3mg+33.3mg capsules
90975998 Amiodarone 300mg/10ml solution for injection pre-filled syringes
91373997 Ciclosporin 10mg capsules
91373998 Ciclosporin 100mg/ml oral solution sugar free
91413998 Lopinavir 133.3mg / ritonavir 33.3mg capsules
92244990 Ciclosporin 100mg capsules
92245990 Ciclosporin 50mg capsules
92246990 Ciclosporin 25mg capsules
92435997 Nelfinavir 50mg/g oral powder

92435998 Nelfinavir 250mg tablets
92544979 Ciclosporin 50mg capsules
92552979 Ciclosporin 100mg capsules
92555979 Ciclosporin 100mg capsules
92558979 Ciclosporin 25mg capsules
92559979 Ciclosporin 25mg capsules
92989996 Ciclosporin 100mg capsules
92989997 Ciclosporin 50mg capsules
92989998 Ciclosporin 25mg capsules
93044992 Itraconazole s/f 50 mg/5ml liq
93044992 Itraconazole s/f 50 mg/5ml liq
93053992 Itraconazole 50mg/5ml oral solution sugar free
93053992 Itraconazole 50mg/5ml oral solution sugar free
93695990 Itraconazole 100mg capsules
93695990 Itraconazole 100mg capsules
93778979 Itraconazole 100mg capsules
93778979 Itraconazole 100mg capsules
93829979 Miconazole 20mg/g oromucosal gel sugar free
94427998 Itraconazole 100mg capsules
94427998 Itraconazole 100mg capsules
94428997 Itraconazole 250mg/25ml solution for infusion ampoules and diluent
94428997 Itraconazole 250mg/25ml solution for infusion ampoules and diluent
94428998 Itraconazole 100mg capsules
94428998 Itraconazole 100mg capsules
94457998 Miconazole 250mg tablet
94458998 Miconazole 20mg/g oromucosal gel sugar free
94593997 Ciclosporin 10mg capsules
94593998 Ciclosporin 50mg capsules
94799990 Itraconazole 100mg capsules
94799990 Itraconazole 100mg capsules
96037997 Ketoconazole 100mg/5ml oral suspension
96037998 Ketoconazole 200mg tablets
96308992 Ketoconazole 50 mg tab
96580998 Ciclosporin 250mg/5ml solution for infusion ampoules
96581996 Ciclosporin 100mg capsules
96581997 Ciclosporin 25mg capsules
96581998 Ciclosporin 100mg/ml oral solution sugar free
96933989 Amiodarone 200mg tablets
96933990 Amiodarone 100mg tablets
96950996 Amiodarone 150mg/3ml solution for injection ampoules
96950997 Amiodarone 200mg tablets
96950998 Amiodarone 100mg tablets
96972989 Amiodarone 200mg tablets
97011989 Amiodarone 200mg tablets
97085989 Amiodarone 200mg tablets
97223992 Ciclosporin 250mg/5ml solution for infusion ampoules
97848989 Amiodarone 200mg tablets
97986989 Amiodarone 200mg tablets
97986990 Amiodarone 100mg tablets
98173998 Nelfinavir 250mg tablets

98238996 Ciclosporin 25mg capsules
98238998 Ciclosporin 100mg/ml oral solution sugar free
98343989 Amiodarone 200mg tablets
98343990 Amiodarone 100mg tablets
98950997 Ciclosporin 50mg capsules
98950998 Ciclosporin 100mg capsules
99350997 Ketoconazole 100mg/5ml oral solution
99350998 Ketoconazole 200mg tablets
99790998 Miconazole 250mg tablets
99791998 Miconazole 20mg/g oromucosal gel sugar free
99811996 Amiodarone 150mg/3ml solution for injection ampoules
99811997 Amiodarone 200mg tablets
99811998 Amiodarone 100mg tablets
99872989 Amiodarone 200mg tablets
99872990 Amiodarone 100mg tablets

Steroids

drugcode	genericname
52504979	Dexamethasone 2mg/5ml oral solution sugar free
53807979	HYDROCORTISONE 5mg m/r tablets
53808979	Hydrocortisone 5mg modified-release tablets
53809979	HYDROCORTISONE 20mg m/r tabs
53810979	Hydrocortisone 20mg modified-release tablets
59794979	Hydrocortisone 5mg/5ml oral suspension sugar free
63230979	Hydrocortisone 100mg suppositories
63611979	Hydrocortisone 2.5mg capsules
63726979	Hydrocortisone 2mg capsules
64761979	Hydrocortisone 100mg/2mL inj
64762979	Hydrocortisone sodium succinate 100mg powder for solution for injection vials
65924979	Prednisolone 5mg/5ml oral solution
66084979	Prednisolone 25mg/5ml oral suspension
66088979	Prednisolone 20mg/5ml oral suspension
66090979	Prednisolone 20mg/5ml oral solution
66092979	Prednisolone 2.5mg/5ml oral suspension
67795979	Hydrocortisone 3mg/5ml oral suspension
69468979	Fludrocortisone 30micrograms/5ml oral suspension
69470979	Fludrocortisone 20micrograms/5ml oral suspension
70200979	Dexamethasone 2mg/5ml oral suspension
70214979	Dexamethasone 16mg/5ml oral solution
79760979	Hydrocortisone 5mg/5ml oral suspension
80070979	Fludrocortisone 50micrograms/5ml oral suspension
80076979	Fludrocortisone 25micrograms/5ml oral suspension
80402979	Dexamethasone 1mg/5ml oral solution
81911998	PREDNISONE 5mg m/r tablets
81912998	PREDNISONE 2mg m/r tablets
81913998	PREDNISONE 1mg m/r tablets
81914998	Prednisone 5mg modified-release tablets
81915998	Prednisone 2mg modified-release tablets
81916998	Prednisone 1mg modified-release tablets
82607998	DEFLAZACORT 6mg tablets
83565978	PREDNISOLONE 5mg e/c tablets
84044998	Hydrocortisone 10mg/5ml oral suspension
85183998	Dexamethasone 6.6mg/2ml solution for injection vials
85184998	Dexamethasone 3.3mg/1ml solution for injection ampoules
85248998	HYDROCORTISONE 500mg injection
85249998	HYDROCORTISONE 100mg/1mL inj
85250998	Hydrocortisone sodium phosphate 500mg/5ml solution for injection ampoules
85251998	Hydrocortisone sodium phosphate 100mg/1ml solution for injection ampoules
85282978	Dexamethasone 6.6mg/2ml solution for injection ampoules
85348998	METHYLPREDNIS 120mg/3mL inj
85349998	METHYLPREDNISOL 80mg/2mL inj
85350998	METHYLPRED ACET 40mg/1mL inj
85351998	Methylprednisolone acetate 120mg/3ml suspension for injection vials

85352998	Methylprednisolone acetate 80mg/2ml suspension for injection vials
85353998	Methylprednisolone acetate 40mg/1ml suspension for injection vials
85744998	TRIAMCINOLONE 80mg/2mL inj
85745998	TRIAMCINOLONE ACE 40mg/1mL inj
85746998	TRIAMCINOLONE ACE 50mg/5mL inj
85747998	TRIAMCINOLONE ACE 10mg/1mL inj
85748998	Triamcinolone acetonide 80mg/2ml suspension for injection pre-filled syringes
85752998	Triamcinolone acetonide 40mg/ml suspension for injection
85754998	Triamcinolone acetonide 50mg/5ml suspension for injection vials
85755998	Triamcinolone acetonide 10mg/1ml suspension for injection ampoules
86220979	Hydrocortisone 100mg/2mL inj
86221979	Hydrocortisone sodium succinate 100mg powder and solvent for solution for injection vials
86464998	Dexamethasone 8mg/2ml solution for injection vials
86465998	Dexamethasone 4mg/1ml solution for injection ampoules
87704998	Cortisone 5mg capsules
87950998	Hydrocortisone 2.5mg muco-adhesive buccal tablets sugar free
88905979	Prednisolone 15mg/5ml oral suspension
88912979	Prednisolone 10mg/5ml oral solution
88926979	Triamcinolone acetonide 40mg/1ml suspension for injection vials
89443996	DEFLAZACORT 1mg tablets
89443997	DEFLAZACORT 30mg tablets
89443998	DEFLAZACORT 6mg tablets
89446996	Deflazacort 1mg tablets
89446997	Deflazacort 30mg tablets
89446998	Deflazacort 6mg tablets
90188979	Prednisone 50mg tablets
91055979	Hydrocortisone 2.5mg muco-adhesive buccal tablets sugar free
91646998	DEXAMETHASONE 2mg/5mL s/f soln
91648990	Prednisolone 2.5mg gastro-resistant tablets
91788990	Prednisolone 5mg soluble tablets
92064990	Hydrocortisone 2.5mg muco-adhesive buccal tablets sugar free
92412998	Hydrocortisone 5mg/5ml Oral solution
92484990	Dexamethasone 500microgram tablets
92484990	Dexamethasone 500microgram tablets
92810997	Dexamethasone 2mg/5ml oral solution
92810998	Dexamethasone 2mg/5ml oral solution sugar free
92861998	Betamethasone 500microgram soluble tablets sugar free
93075997	Prednisolone sodium phosphate 0.05% eye drops
93075998	Prednisolone 5mg soluble tablets
93098990	Dexamethasone 2mg tablets
93362979	TRIAMCINOLONE ACE 40mg/1mL inj
93363979	Triamcinolone acetonide 40mg/1ml suspension for injection pre-filled syringes
93400979	METHYLPRED ACET 40mg/1mL inj
93401979	METHYLPRED ACET 40mg/1mL inj
93402979	METHYLPRED ACET 40mg/1mL inj
93403979	METHYLPRED ACET 40mg/1mL inj
93427979	Dexamethasone 2mg tablets

93429979	Dexamethasone 2mg tablets
93431979	Dexamethasone 500microgram tablets
93435979	BETAMETHASONE 500mcg sol tabs
93493998	Methylprednisolone sodium succinate 125mg powder and solvent for solution for injection vials
93494998	METHYLPREDN 125mg inj pdr+dil
93588998	Hydrocortisone na succinate 2.5mg Lozenge
93643992	BETAMETHASONE VALERATE .1 MG TAB
93645992	BETAMETHASONE .1 MG TAB
93647992	BETAMETHASONE LOZ
93867997	Dexamethasone 100mg/5ml solution for injection vials
93867998	Dexamethasone sodium phosphate 5mg/ml injection
93912996	Prednisolone 50mg tablets
93912997	Prednisolone 5 mg gastro-resistant tablet
93912998	Prednisolone 2.5mg gastro-resistant tablets
94176998	Betamethasone 4mg/1ml solution for injection ampoules
94442992	CORTISONE ACETATE MSD 25 MG TAB
94559992	TRIAMCINOLONE 10 MG INJ
94870992	CORTISONE ACETATE MSD 5 MG TAB
95026992	FLUDROCORTISONE ACETATE 100 MCG TAB
95026992	FLUDROCORTISONE ACETATE 100 MCG TAB
95137996	Triamcinolone acetonide 40mg/ml injection
95137997	Triamcinolone acetonide 80mg/2ml intramuscular injection
95137998	Triamcinolone acetonide 40mg/ml IA/IM
95139997	Triamcinolone 4mg Tablet
95139998	Triamcinolone 2mg Tablet
95357992	DEXAMETHASONE 500mcg tablets
95417996	Prednisolone 5mg gastro-resistant tablets
95417997	Prednisolone 25mg tablets
95417998	Prednisolone 2.5mg tablet
95484992	PREDNISOLONE E/C 1 MG TAB
95487992	PREDNISOLONE 5mg sol tablets
95492992	PREDNISOLONE 10 MG TAB
95492992	PREDNISOLONE 10 MG TAB
95493992	PREDNISOLONE 2 MG TAB
95593990	Prednisolone 5mg tablets
95594990	Prednisolone 1mg tablets
95833992	HYDROCORTISONE SODIUM SUCCINATE POW
95847996	METHYLPREDNISOLONE 16mg tabs
95847997	METHYLPREDNISOLONE 4mg tablets
95847998	METHYLPREDNISOLONE 2mg tablets
95848998	Methylprednisolone acetate 40mg/ml Injection
95849997	Methylprednisolone sodium succinate 2g powder and solvent for solution for injection vials
95849998	Methylprednisolone sodium succinate 1g powder and solvent for solution for injection vials
95850996	Methylprednisolone sodium succinate 500mg powder and solvent for solution for injection vials
95850997	Methylprednisolone sodium succinate 40mg powder and solvent for solution for injection vials

95851996 Methylprednisolone 16mg tablets
95851997 Methylprednisolone 4mg tablets
95851998 Methylprednisolone 2mg tablets
95912990 Prednisolone 25mg tablets
95967998 Betamethasone 500microgram tablets
96143997 Hydrocortisone 20mg tablets
96143998 Hydrocortisone 10mg tablets
96172998 Hydrocortisone na phosphate 100mg/ml Injection
96175998 Hydrocortisone 100mg/2mL inj
96181992 DEXAMETHASONE 8 MG TAB
96182992 DEXAMETHASONE 750 MCG TAB
96182992 DEXAMETHASONE 750 MCG TAB
96245992 FLUDROCORTISONE .05 MG SUS
96245992 FLUDROCORTISONE .05 MG SUS
96269992 HYDROCORTISONE PELLETS 2.5 MG LOZ
96270992 HYDROCORTISONE I/V 100 MG INJ
96361989 Prednisolone 5mg tablets
96361990 Prednisolone 1mg tablets
96409992 PREDNISOLONE 50 MG TAB
96410992 PREDNISONE 50 MG TAB
96411992 PREDNISOLONE 15 MG TAB
96429998 Dexamethasone 20mg/ml shock pack
96430996 Dexamethasone sodium phosphate 4mg/ml intra-artic injection
96430997 Dexamethasone sodium phosphate iv 4mg/ml injection
96430998 Dexamethasone sodium phosphate 4mg/ml injection
96431996 Dexamethasone 500micrograms/5ml oral solution
96431997 Dexamethasone 2mg tablets
96431998 Dexamethasone 500microgram tablets
96526998 Fludrocortisone 100microgram tablets
96526998 Fludrocortisone 100microgram tablets
96577990 Prednisolone 5mg soluble tablets
96598989 Dexamethasone sodium phosphate 4mg/ml injection
96603997 Cortisone 25mg tablets
96603998 Cortisone acetate 5mg tablets
96658992 FLUDROCORTISONE 25 MCG TAB
96658992 FLUDROCORTISONE 25 MCG TAB
96680992 HYDROCORTISONE 100 MG INJ
96718990 Dexamethasone sodium phosphate 5mg/ml injection
96743992 PREDNISONE 2.5 MG TAB
96744992 PREDNISOLONE 4 MG TAB
97101989 Prednisolone 5mg gastro-resistant tablets
97101990 Prednisolone 2.5mg gastro-resistant tablets
97155997 Prednisolone 5mg tablets
97155998 Prednisolone 1mg tablets
97156997 Prednisone 5mg tablets
97156998 Prednisone 1mg tablets
97203992 CORTISONE ACETATE 2.5 MG TAB
97204992 CORTISONE ACETATE 25 MG INJ
97204992 CORTISONE ACETATE 25 MG INJ
97240992 DELTACORTRIL 1 MG TAB

97243992 DECADRON 2 MG TAB
97341992 EFCORTELAN 100 MG INJ
97436998 PREDNISOLONE 25mg tablets
97451992 FLUDROCORTISONE 75 MCG TAB
97451992 FLUDROCORTISONE 75 MCG TAB
97452992 FLUDROCORTISONE 20 MCG TAB
97492997 HYDROCORTISONE 20mg tablets
97492998 HYDROCORTISONE 10mg tablets
97493998 DEXAMETHASONE 100mg/5mL inj
97499997 DEXAMETHASONE 8mg/2mL inj
97499998 DEXAMETHASONE 8mg/2mL inj
97502998 DEXAMETHASONE 500mcg tablets
97511998 PREDNISOLONE 32mg/2mL inj
97583992 HYDROCORTISONE 25 MG TAB
97640997 Hydrocortisone na succinate 500mg/vial Injection
97640998 Hydrocortisone na succinate 100mg/vial Injection
97726990 Prednisolone 2.5mg gastro-resistant tablets
97739992 METHYLPREDNISOLONE 40 MG/ML INJ
97740992 METHYLPREDNISOLONE L/A 4 MG CAP
97829998 METHYLPREDNISOLONE 100mg tabs
97832998 Methylprednisolone 100mg tablets
97835996 TRIAMCINOLONE 40mg/1mL syrng
97835997 TRIAMCINOLONE 40mg/1mL syrng
97835998 TRIAMCINOLONE 40mg/1mL syrng
97902998 METHYLPREDNISOL 1g inj pdr+dil
97903996 METHYLPREDN 500mg inj pdr+dil
97903997 METHYLPREDNIS 40mg inj pdr+dil
97923998 METHYLPREDNIS 120mg/3mL inj
97929990 Prednisolone 5mg tablets
97942992 PREDNISONE 10 MG TAB
97943992 PREDNISONE 30 MG TAB
98072989 Dexamethasone 500micrograms/5ml oral solution
98072990 Dexamethasone 2mg/5ml oral solution sugar free
98107990 Prednisolone 5mg tablets
98221998 Triamcinolone acetonide 10mg/ml IA/ID
98326997 TRIAMCINOLONE 4mg tablets
98326998 TRIAMCINOLONE 2mg tablets
98376998 Hydrocortisone na succinate 100mg/vial Injection
98394998 BETAMETHASONE 500mcg sol tabs
98395998 BETAMETHASONE 4mg/1mL inj
98455990 Prednisolone 5mg gastro-resistant tablets
98456988 Prednisolone 1mg tablets
98456989 Prednisolone 5mg gastro-resistant tablets
98491998 PREDNISOLONE ACET 25mg/1mL inj
98514997 PREDNISOLONE 5mg tablets
98514998 PREDNISOLONE 1mg tablets
98562997 PREDNISOLONE 5mg e/c tablets
98562998 PREDNISOLONE 2.5mg e/c tablets
98644989 Dexamethasone 2mg tablets
98644990 Dexamethasone 500microgram tablets

98724997	DEXAMETHASONE 2mg tablets
99098990	Prednisone 5mg tablets
99099988	Prednisolone 5mg tablets
99099989	Prednisolone 5mg gastro-resistant tablets
99099990	Prednisolone 2.5mg gastro-resistant tablets
99100989	Prednisolone 5mg tablets
99100990	Prednisolone 1mg tablets
99137998	PREDNISOLONE 5mg tablets
99226998	PREDNISOLONE 5mg sol tablets
99228997	PREDNISOLONE 5mg tablets
99228998	PREDNISOLONE 1mg tablets
99305998	Triamcinolone acetonide 10mg/ml IA/ID
99423988	Prednisolone 2.5mg gastro-resistant tablets
99423989	Prednisolone 5mg tablets
99423990	Prednisolone 1mg tablets
99424989	Prednisolone 5mg tablets
99424990	Prednisolone 1mg tablets
99425988	Prednisolone 5mg gastro-resistant tablets
99425989	Prednisolone 5mg tablets
99425990	Prednisolone 1mg tablets
99512998	TRIAMCINOLONE 80mg/2mL syring
99550998	HYDROCORTISONE 20mg tablets
99699998	HYDROCORTISONE 100mg/1mL inj
99781998	PREDNISONE 5mg tablets
99802998	CORTISONE 25mg tablets
99803997	CORTISONE 25mg tablets
99803998	CORTISONE 5mg tablets
99804998	CORTISONE 25mg tablets
99806998	HYDROCORTISONE 2.5mg lozenges
99890998	BETAMETHASONE 500mcg tablets
99896992	HYDROCORTISONE SODIUM PHOSPHATE 5 MG SOL
99984997	TRIAMCINOLONE ACE 10mg/1mL inj
99984998	TRIAMCINOLONE ACE 10mg/1mL inj
