# Supplementary material for: Cohort study investigating the relationship between cholesterol, cardiovascular risk score and the prescribing of statins in UK primary care: study protocol
Source: BMJ Open. 2016 Nov 17;6(11):e013120. doi: 10.1136/bmjopen-2016-013120 (PMC5128938; doi:10.1136/bmjopen-2016-013120)
Supplement: supplementary appendix III [file bmjopen-2016-013120supp_appendixIII.pdf]

Appendix III – Explanation of terms

Townsend Quintile

The Townsend Deprivation index is a measure of population deprivation based on four variables originally described by Peter Townsend in 1988 [1]. The variables are:

1) Unemployment: Based on the percentage of adults who are economically active
2) Car ownership
3) Home ownership
4) Household overcrowding

These data are gathered via census data and areas are scored in the UK using postcodes areas. For the purposes of this research, these scores are splint into quintiles [2].

Rurality index

Areas are classified as either urban or rural depending on the percentage of their population being resident in rural areas. This classification is done but the Office for National Statistics [3]. In this data, the 6 classifications will be split into 2 groups, rural and urban [2].

1. Townsend, P., P. Phillimore, and A. Beattie, *Health and deprivation: inequality and the North*. 1988: Routledge.
2. IMS Health, *THIN Data Guide for Researchers*. 2015.
3. Peter Bibby, P.B., *Urban and Rural Classification of English Local Authority Districts and Similar Geographical Units in England: Methodology*. 2016, Office for National Statistics, Department for Environment, Food & Rural Affairs: London.
